# Machine Learning Prediction of Biomarkers from SNPs and of Disease Risk from Biomarkers in the UK Biobank

**DOI:** 10.3390/genes12070991

**Published:** 2021-06-29

**Authors:** Erik Widen, Timothy G. Raben, Louis Lello, Stephen D. H. Hsu

**Affiliations:** 1 Department of Physics and Astronomy, Michigan State University, 567 Wilson Rd, East Lansing, MI 48824, USA; rabentim@msu.edu (T.G.R.); hsu@msu.edu (S.D.H.H.); 2 Genomic Prediction, Inc., 675 US Highway One, North Brunswick, NJ 08902, USA

**Keywords:** polygenic scores, disease risk, machine learning, atherosclerotic cardiovascular disease, biomarkers

## Abstract

We use UK Biobank data to train predictors for 65 blood and urine markers such as HDL, LDL, lipoprotein A, glycated haemoglobin, etc. from SNP genotype. For example, our Polygenic Score (PGS) predictor correlates ∼0.76 with lipoprotein A level, which is highly heritable and an independent risk factor for heart disease. This may be the most accurate genomic prediction of a quantitative trait that has yet been produced (specifically, for European ancestry groups). We also train predictors of common disease risk using blood and urine biomarkers alone (no DNA information); we call these predictors biomarker risk scores, BMRS. Individuals who are at high risk (e.g., odds ratio of >5× population average) can be identified for conditions such as coronary artery disease (AUC∼0.75), diabetes (AUC∼0.95), hypertension, liver and kidney problems, and cancer using biomarkers alone. Our atherosclerotic cardiovascular disease (ASCVD) predictor uses ∼10 biomarkers and performs in UKB evaluation as well as or better than the American College of Cardiology ASCVD Risk Estimator, which uses quite different inputs (age, diagnostic history, BMI, smoking status, statin usage, etc.). We compare polygenic risk scores (risk conditional on genotype: PRS) for common diseases to the risk predictors which result from the concatenation of learned functions BMRS and PGS, i.e., applying the BMRS predictors to the PGS output.

## 1. Introduction

Modern machine learning (ML) methods have opened the door to using high dimensional inputs to predict health outcomes and risk. This paper concerns the application of *sparse* linear ML to genetic and health information in order to make predictions that could be useful in a clinical setting. Recent work has highlighted that ML, in particular polygenic predictors, have high potential impact in clinical settings [1,2,3,4,5,6,7,8,9,10,11,12,13,14,15,16,17,18,19,20,21], especially for coronary artery disease (CAD) [11,12,22]. Additionally, over the past quarter century it has been advocated (e.g., Joint Task Force of the European Society of Cardiology and Other Societies [23], American College of Cardiology(ACC)/American Heart Association (AHA) [24], and the Scottish Intercollegiate Guidelines Network [25]) that physicians should use risk scores based on statistical summaries of biomarkers. Examples of such scores include Framingham [26,27], SCORE [28], ASSIGN–SCORE [29], QRISK1 [30], QRISK2 [31], QRISK3 [32], PROCAM [33], Pooled Cohort Studies Equations [34,35,36], CUORE [37], Globorisk [38], Reynolds risk score [39,40], World Health Organization (WHO) risk chart [41,42], MyRisk_stroke calculator [43], NIPPON [44], and UKPDS risk engine [45,46].

In [47] it was emphasized that cardiovascular disease (CVD) risk scores have a long history. Although early attempts to identify key genetic risk markers had some missteps, Reference [47] argues that—thanks to new methods and larger datasets—genetic risk scores have developed enough to begin being employed in clinical practice (e.g., [22]). Additionally, [48] charts the rise of ML/AI in cardiology in general and predicts that it will play a major role in diagnostic and precision medicine involving cardiology.

Some examples of using ML to predict CVD risk include: In [49] biomarkers—(apo)lipoproteins and cholesterols—were used with a Cox-hazard-model to claim that Total Cholesterol and HDL are sufficient for good CVD risk assessment. Apolipoproteins, LDL, and other lipids in general offered very modest improvements in risk prediction with this model (some of this is medication, e.g., statin, dependent); in [50] a convolutional neural network and gradient boosting algorithms were used on a small subset of biomarkers, imaging results, and medical history (*including ASCVD risk score*) on a sample size of ∼2000 to predict myocardial infarction (heart attack) and death. Including all variables the approach reached an Area Under the Receiver operator characteristic Curve (AUC) 0.82 (95% CI: 77–87); in [51] a support vector machine learning algorithm was used and showed out-performance over the ACC/AHA pooled cohort studies equations. This was measured by recommending less drug therapy while missing fewer events of CVD; in [52] a support vector machine learning classifier algorithm was trained on pre-selected biomarkers, medical and family history, and imaging data. This approach was then compared to standard “statistical scores” for CVD such as Framingham [26,27], SCORE [28], QRISK3 [32], PROCAM [33], Pooled Cohort Studies Equations [34,36], CUORE [37], Globorisk [38], Reynolds risk score [39,40], World Health Organization (WHO) risk chart [41,42], MyRisk_stroke calculator [43], NIPPON [44], and UKPDS risk engine [45,46]. Additional examples of using ML primarily on single nucleotide polymorphism (SNP) data include: in [12], LDpred [53] was used to predict coronary artery disease (CAD) and found, using additional common covariates like age and sex, an AUC∼0.81 (95% CI: 0.80–0.81 ); using *only* SNP information, in [11] a sparse ML approach was used to predict heart atrial fibrillation (AUC∼0.64), hypertension (AUC∼0.65), and heart attack (AUC∼0.59); and in [22] a meta-analysis was done to generate a hazard ratio (HR) for CAD of 1.71 (95% CI: 1.68–1.73). AUC has become a standard metric for evaluating polygenic scores for disease risk. However, one of the most promising applications of these scores is their ability to identify risk outliers, as highlighted in [11,54]. Outlier identification often works well, even for predictors with only moderately strong AUCs.

This work applies ML to both SNP based prediction and biomarker based prediction. We focus on a type of *sparse* ML (LASSO) which does *feature selection*, as well as *relative weighting*. In other words, sparse ML selects a subset of all the possible variables and then gives them relative weights. This is in contrast to most of the statistical methods used above where the inclusion of biomarkers relied mostly on previous research on the biomarkers themselves. In addition to feature selection, this sparse approach has been chosen because of previous success with SNP based prediction [11,54,55,56,57,58,59] and because it has been shown to be among the best ML predictors for genetics and is often a good all around method [56,60].

**Figure 1:**
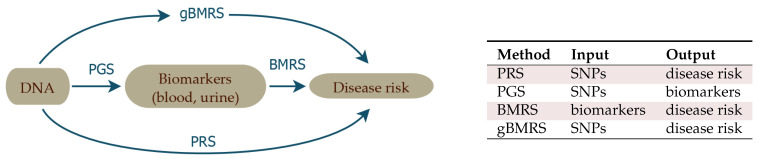
The four different types of predictors appearing in this paper.

The source of data for this work is the UK Biobank (UKB) [61], which includes SNP genotypes, medical diagnosis information, and extensive biomarker information (i.e., 65 quantitative outputs of blood and urine tests) for almost 500 k individuals. In this article, we describe *sparse* ML investigations of the correlation structure between these three categories of data. As described in Figure 1, we train:

1. **PGS** Polygenic Score (PGS) predictors of the quantitative biomarker test results from *SNPs alone*. These functions predict biomarker level conditional on genotype: PGS.
  For example, we predict measured lipoprotein A levels from SNPs, achieving a correlation of 0.76 between PGS and actual biomarker level. The goal with this training is to study how accurately biomarker levels can be predicted, to investigate the underlying genetic architecture, and to be used as input for the predictors in point 3 below.
2. **BMRS** Biomarker Risk Scores (BMRS) which predict risk of a specific disease condition *using only measured biomarkers as input*: BMRS.
  For example, our atherosclerotic cardiovascular disease (ASCVD) predictor uses ∼10 blood biomarkers to predict disease risk. We show that in UKB validation it predicts disease risk as well as, or better than, the American College of Cardiology ASCVD Risk Estimator [62,63], which uses quite different inputs, such as age, diagnostic history, body mass index (BMI), smoking status, statin usage, etc. Liver and kidney problem risk prediction from biomarkers seems quite promising, based on our results. In total, we investigate predictions for ASCVD, CAD, diabetes type I and II, hypertension, very inclusive definitions of kidney and liver problems, and obesity.
3. **gBMRS and PRS** Finally, by concatenating the predictors in 1 and 2 above, we build functions which map genotype (SNPs) to disease risk, with biomarkers as an intermediate step. We denote these concatenated predictors as: Genetic Biomarker Risk Scores (gBMRS). We emphasize that concatenation (i.e., F(G(x))) is *not* the same as training with both biomarkers and SNPs simultaneously used as features. The concatenated predictors *only* require SNPs as input, but use SNP predicted biomarker values as an *intermediate step* in calculation of the predicted disease risk. These functions can be compared to standard Polygenic Risk Scores (PRS) computed directly from SNPs, using disease case status as the training phenotype: PRS.
  For example, the concatenated function which maps SNPs → biomarkers → type 2 diabetes risk performs roughly, as well as the PRS for type 2 diabetes ( AUC ∼ 0.64).
  We study this concatenation to see whether this alternative path to disease prediction can provide additional gains relative to the more straight-forward standard PRS.

To conform to the standard journal section structure, these three related but separate predictor types are discussed in a staggered disposition throughout the *Materials and Methods* and *Results* sections.

From our investigations, we conclude that many biomarker levels are not just substantially heritable, but can be predicted with some accuracy from SNPs. This is true despite the fact that levels fluctuate from day to day for a specific individual. We also conclude that disease risk prediction from biomarkers alone, via BMRS, is potentially very powerful, and indeed complementary to existing methods for risk estimation. For example, we show below that the ASCVD Risk Estimator uses different and complementary information to the biomarkers used in our ASCVD BMRS. Our results suggest that combining this complementary information can lead to stronger prediction and perhaps new insights into heart disease. Significant analyses of the costs and benefits of additional inputs have been performed for the existing ASCVD predictor, which is in clinical use (e.g., [62,63]), including some of the features in our predictor. Our comparison is limited to risk predictor *performance* and in the UKB cohort only.

We validate all predictors using sibling data: most of the power to differentiate between siblings (either in quantitative trait values or disease risk) persists despite similarity in childhood environments. We also test the fall off in power in distant ancestries (relative to the European training population). The decline for SNP based predictors varies as expected with genetic distance, whereas biomarker prediction does not display this pattern.

Throughout this paper, we refer to the different biomarkers according to the abbreviations listed in Table 1.

## 2. Materials and Methods

We outline here common features and methods that are used throughout this project. Later, in subsections below, we will detail the specifics that are unique to each sub-analysis.

- Subject data

All research in this paper uses data exclusively from the 2018 UKB release [61,64] and updates (see Supplementary Information for more details). All statements about sex or ancestry refer to the self-reported data within this dataset [65]. There is of course a complicated genetic substructure within each one of these subgroups [66,67,68,69,70,71,72,73,74,75,76,77,78,79]. However, previous research has demonstrated that—when looking at the UK Biobank, using sparse methods and looking at heart and blood related phenotypes—self-report can provide sufficiently good data for training purposes [54,55,56,57,58]. Genetic prediction in general depends on non-trivial factors including population substructure, size of training sets, algorithms (e.g., sparse vs non-sparse methods), heritability, environmental factors, and etc. Nonetheless, in some instances self-reported identity is sufficient for training. Sparse, self-reported training in the UKB has been compared to analyses regressing on principal components (PC) [55], compared to training and testing across adjacent ancestry groups as defined by PCs [11,80], and detailed sibling tests [58]. For the purposes of this paper, we compared predictors trained on self-reported ancestry vs regressing on residuals from a principal component analysis (PCA) where we find the results differ by ∼1% (additional details in the results section). Additionally, using a principal component analysis to account for population substructure can be problematic on small sample sizes (e.g., [81]). In order to further demonstrate that population substructure has negligible effect on the presented prediction power, we also use a sibling validation method [58]: All sets of siblings are withheld from training and used for final testing. Environmental background, such as life style and diet, and indirect genetic effects have impacts on most of the biomarkers and siblings generally have more similar backgrounds than randomly chosen pairs, and are also more genetically similar than unrelated individuals. Retained predictive power among siblings is, hence, a strong indication of direct genetic effects. Moreover, the amount of lost power as compared to the general population can give some idea of the magnitude of environmental effects, e.g., from childhood environment. (There can also be genetic nurture [82,83,84,85,86] effects that are not analyzed here).

We refer to the self-reported ancestries labeled white, Asian, Chinese, and black in UKB as European, South-Asian, East-Asian, and African, in accordance with the guidelines in [87]. It has been repeatedly confirmed that the power of polygenic predictors is dependent on both the training and testing ancestries, and that generally the power of the prediction falls off as a function of genetic distance [88,89,90,91]. All individuals with self-reported admixture were excluded from this study.

- Phenotype data

The phenotypes included in the paper include self-reported UKB statuses, biomarker measurements (sometimes repeated), standard (ICD9, ICD10, OPCS3, OPCS4) codes, diagnosed conditions, thresholds, and combinations of all the previous items. Full details of how each phenotype is defined is given in the Supplementary Information.

- Genotype data

The UKB genotype data were quality controlled by excluding all SNPs with less than 3% call success rate and also those with a minor allele frequency (MAF) <0.001. All individuals with less than 3% successfully called SNPs were also excluded and, again, any individual with self-reported mixed ancestry was excluded from this study entirely. Furthermore, only autosomal genetic information was used, including SNPs located on chromosomes 1–22 only.

- Algorithms/Machine Learning

This work primarily focuses on LASSO [92], or compressed sensing [93,94,95,96]. LASSO was chosen because it has been repeatedly shown that sparse, linear methods are among the most successful in genetic prediction over a wide variety of traits [11,55,58]. Additionally, sparsity makes application and analysis of the predictors much more computationally efficient. As genetic predictors move into clinical settings, it will undoubtedly be the case that optimal prediction algorithms will vary depending on phenotype and training data, but LASSO currently serves as an excellent jack-of-all-trades.

- Statistical evaluation

**Figure 2:**
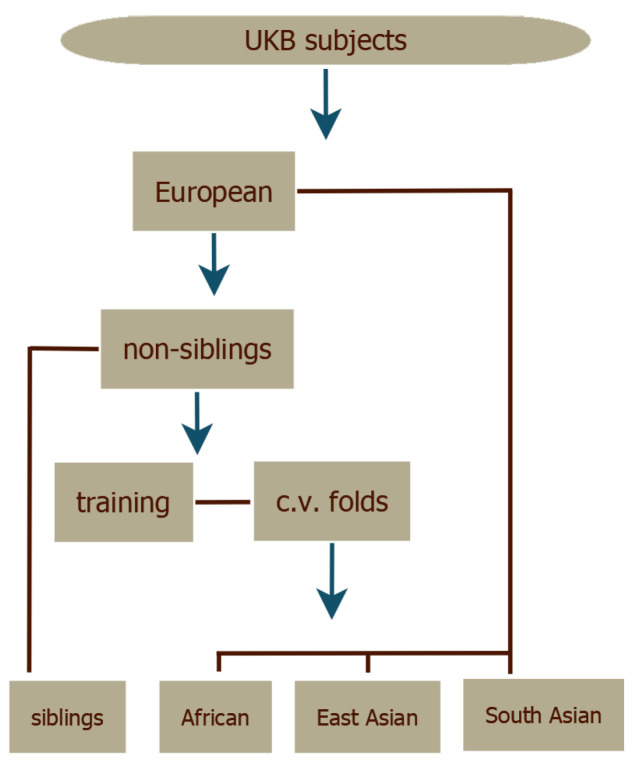
The dataset splits in the general training process. (1) European, African, East Asian, and South Asian ancestry groups are made from the UKB subject data. (2) The large European set is split into sibling and non-sibling sets. (3) The non-sibling set is further split into training sets and cross-validation (c.v.) folds. (4) Final predictors are tested on the remaining groups.

The LASSO predictors were evaluated with cross-validation. The validation set for each fold was used to choose optimal values of the regularization parameter λ (see Supplementary Information). The UKB subjects were divided according to self-reported ancestry and the European subset was then split into siblings as evaluation set and non-siblings as training set. The resulting training set was then split into five cross-validation folds, as shown in Figure 2. The top performing predictor—as measured by performance in the European corresponding validation set—from each fold was retained providing some statistics for the uncertainty estimates in the results. More details can be found in the Supplementary Information.

We now present in separate subsections the detailed methods used for PGS, BMRS and gBMRS, respectively, and we end with the methods for comparing our ASCVD predictor with the clinically employed ASCVD Risk Estimator.

### 2.1. PGS: Predicting Biomarkers from SNPs

We used LASSO to predict the 65 types of biomarkers listed in Table 1 from SNP data, and refer to these type of predictors as PGS.

*Data and pre-processing:* UKB contains data from repeated visits and for samples with more than one measurement of a certain biomarker the average value was taken in order to measure the heritable levels rather than fluctuations. These raw measurements were z-scored for men and women separately and consecutively age corrected by subtracting a linear regression on age-biomarker data obtained from averaging the biomarker value for all samples born the same year (biomarker-age plots are contained in Supplementary Information). The parameters for the pre-processing were determined from training sets with about 340 k samples of European ancestry. Evaluation sets of about 20–40 k European siblings and all non-European individuals were withheld entirely from training but pre-processed with the same parameters.

*Predictor training:* Five LASSO predictors were trained on each biomarker. Validation sets were made from randomly drawing 1000 samples and excluding these from the training set.

As a separate check for population structure, we adjusted phenotypes based on a linear regression on the 20 first principal components (as provided by UKB) of the genotypes. This was done for five of the biomarker predictors which correlated highly with the original phenotypes. We then trained LASSO predictors on the residuals. This had negligible effect (∼1%) as shown below in the result section.

*Evaluation:* Predictors were judged based on the correlation between predicted and measured biomarkers. Each predictor, for each biomarker and cross-validation fold, was evaluated on its corresponding evaluation set consisting of ∼20–40 k samples of European siblings. To test the performance dependence on ancestry we also applied the predictors to the 9 k of South-Asian, 1500 of East-Asian, and 7 k of African ancestry. In Section 3.2.2, we report the correlation between the PGS and the phenotypes as the performance metric for these continuous traits. The sibling evaluation consisted of calculating, for pair of samples, the difference in phenotype $\Delta_{\mathrm{phen}}$ and the difference in PGS $\Delta_{\mathrm{PGS}}$ and comparing the correlations between these quantities $\mathrm{corr}(\Delta_{\mathrm{phen}},\Delta_{\mathrm{PGS}})$ within random and sibling pairs, respectively.

*Genetic architecture:* One can define the variance accounted for by each SNP *i* in a predictor according to

(1) $$\mathrm{variance}\;\mathrm{accounted}\;\mathrm{for}\;\mathrm{by}\;{\mathrm{SNP}}_{i}=\beta_{i}^{2}\left(1-f_{i}\right)f_{i}\;,$$

where $f_{i}$ is the MAF of SNP *i* and $\beta_{i}$ is the corresponding coefficient size. This is described in greater detail in the Supplementary Information and in [57]. We use this alongside Manhattan-plots of the effect sizes β in the results (Section 3.1.1) to display the genetic architectures of the top 3 performing PGS predictors. Analogous plots for the rest of the PGS predictors are contained in the Supplementary Information.

### 2.2. Methods for Disease Prediction: BMRS and gBMRS

We used two approaches to investigate whether biomarkers can be used to predict disease risk, analogous to how blood tests are used clinically.

1. **BMRS** We trained predictors with LASSO to predict case/control status directly from phenotypes, i.e., using the direct biomarker measurements as features. We denote this type of predictor as biomarker risk score (BMRS).
2. **gBMRS** Second, we applied the already trained BMRS predictors to the *predicted* phenotypes, i.e., using the biomarker PGS output from the SNP-based predictors in Section 2.1 as input. As such, we obtain disease risk scores using only SNP data as input. We denote these concatenated predictors as *genetic* biomarker risk scores (gBMRS).

We evaluated this strategy on eight different condition definitions and we present the details for the two approaches separately. This is done both to display the performance dependence on the two approaches and since the predictions from biomarkers are very interesting in their own right.

#### 2.2.1. BMRS: Predicting Case Status from Biomarkers

*Condition definitions:* Based on the available UKB data, we defined conditions for CAD, cancer, diabetes type 1, diabetes type 2, hypertension, kidney problem, liver problem, and obesity. The detailed definitions for each one of these are to be found in the Supplementary Information. In general, we chose the definitions to be inclusive; kidney (liver) problem for example contains almost all kidney (liver) related problems that are reported in UKB, whereas cancer refers to any type of cancer. Obesity was defined as a BMI over 30. The effects of changing definitions are further discussed in Section 4.

*Predictor training:* We used 62 out of the 65 biomarkers as input features, dropping E2, MA, and RF due to few available measurements, and taking the first available measurement for each sample (we did not use averages for the BMRS to closer resemble a clinical setting). The raw data were pre-processed by sex specific z-scoring and then age correcting by subtracting a linear regression. Using LASSO, we then trained 5 predictors on the case/control status, choosing optimal λ by five-fold cross-validation. The training was done separately for men (*N* = 106,656) and women (*N* = 86,193) and on European ancestry only.

We again conducted a separate check for population structure, by training on the residuals after subtracting a linear regression on the 20 first principal genotype components from the phenotypes. As for the PGS, this had no significant effect (∼1%).

*Evaluation:* As was done for the PGS in Section 2.1, about 40 k siblings of European ancestry and all non-European individuals were kept separate from all training and were used as evaluation sets. We measured the predictor performance by AUC and by odds ratio plots. We conducted additional sibling tests for the BMRS predictors to test for environmental effects: we applied the predictors to pairs of siblings with precisely one case and one control and report the fraction of correctly called affected sibling, juxtaposed with the same results for random pairs of one case and one control.

It should be emphasized here that we did not take the date of onset into account in this study: disease status was considered on a “life span” (as far as UKB covers) basis such that cases could have onsets both prior to and after the time of the biomarker measurement. Prediction in this sense means what can we predict about current or future case status only knowing a set of momentary biomarker values. Temporal prediction tests (i.e., prospective prediction) are deferred to later work. The distributions of time differences between condition onsets and the first biomarker measurement can be found in the Supplementary Information.

#### 2.2.2. gBMRS: Predicting Case Status from PGS of Biomarkers

To form predictors taking SNP data as input, we concatenated the PGS predictors from Section 2.1 with the biomarker predictors BMRS to form what we call gBMRS. The BMRS disease predictors were taken as is and applied to the z-scored PGS output of the predictors in Section 2.1. No further training was done and the performance was evaluated as for and compared with the BMRS predictors.

### 2.3. Comparison with ASCVD Risk Estimator

Finally, for the *Materials and Methods* section, we describe the method of comparison between our ASCVD BMRS predictor and the ASCVD Risk Estimator [63]. The latter is a widely used tool to aid clinicians in risk estimations of and preventative care against atherosclerotic cardiovascular disease. We used this well-established resource for an exemplifying benchmark of BMRS predictors by training a predictor on this condition specifically. ASCVD aggregates several sub-diagnoses and exists in different versions. Hard ASCVD includes acute coronary syndromes, death by coronary heart disease, a history of myocardial infarction, and fatal and non-fatal stroke. A more general (extended) ASCVD definition additionally includes stable or unstable angina, coronary or other arterial revascularization, transient ischemic attack, and peripheral arterial disease presumed to be of atherosclerotic origin. We used a UKB specific extended definition, detailed in the Supplementary Information. The ASCVD Risk Estimator requires the input: age, sex, race, systolic and diastolic blood pressure, total cholesterol, HDL, LDL, history of diabetes, smoking status, time since quit smoking (if applicable), whether on hypertension treatment, whether on a statin, and whether on aspirin. It can also use previous data for follow-ups but we restricted our analysis to “first visit patients” only. All of these data fields can be found in some form in the UKB (the exact field choices are listed in the Supplementary Information).

The outputs of the ASCVD Risk Estimator are (up to) three risk estimates: 10 year risk, lifetime risk, and optimal risk, all given as a percentage. Since our UKB data only cover approximately 10 years from the first biomarker measurement, we exclusively used the 10 year risk output. We applied the underlying function of the ASCVD Risk Estimator to the corresponding data in UKB and obtained a 10 year risk estimate for 358,650 individuals for whom we also had an ASCVD case/control status. Strictly speaking, the ASCVD Risk Estimator was developed for North American cohorts and based on hard ASCVD but, as seen in Section 3.3, performed very well also in the cohorts of the UKB using the extended definition. Note, however, the current comparison is intended as an illustration of the power of BMRS and not as a rigorous test for deployment (see the *Discussion* below).

We then trained a BMRS predictor on case/control status, analogously to Section 2.2, but using ordinary linear regression on the z-scored biomarker measurements. This outputs a risk *score* which we mapped to absolute risk *estimates in percentages* as follows. The risk scores obtained from applying the predictor on the training data were binned and, within each bin, the disease prevalence was calculated from the case/control statuses as an estimated risk for samples with the corresponding risk scores. This discrete mapping was then made continuous using rolling averages and linear interpolation. For details see Supplementary Information.

#### Combination of Predictor from Biomarkers and the ASCVD Risk Estimator

In the results Section 3.3, we show that the ASCVD BMRS predictor and the ASCVD Risk Estimator are making complementary predictions. We, therefore, also tested a combination of them. We made a linear regression on all the input features from the two predictors combined (65 continuous and 8 discrete variables), z-scoring the discrete variables from the ASCVD Risk Estimator input such that everything was on the same scale. In addition, we made a second regression also including the *output* of the ASCVD Risk Estimator to capture the non-linearities within that function. These regressions were made and evaluated on the same training and evaluation sets as for the BMRS predictors.

## 3. Results

As with the section of *Materials and Methods*, we present the results for PGS, BMRS, gBMRS, and the ASCVD comparison in separate subsections.

### 3.1. Predicting Biomarkers from SNPs

**Figure 3:**
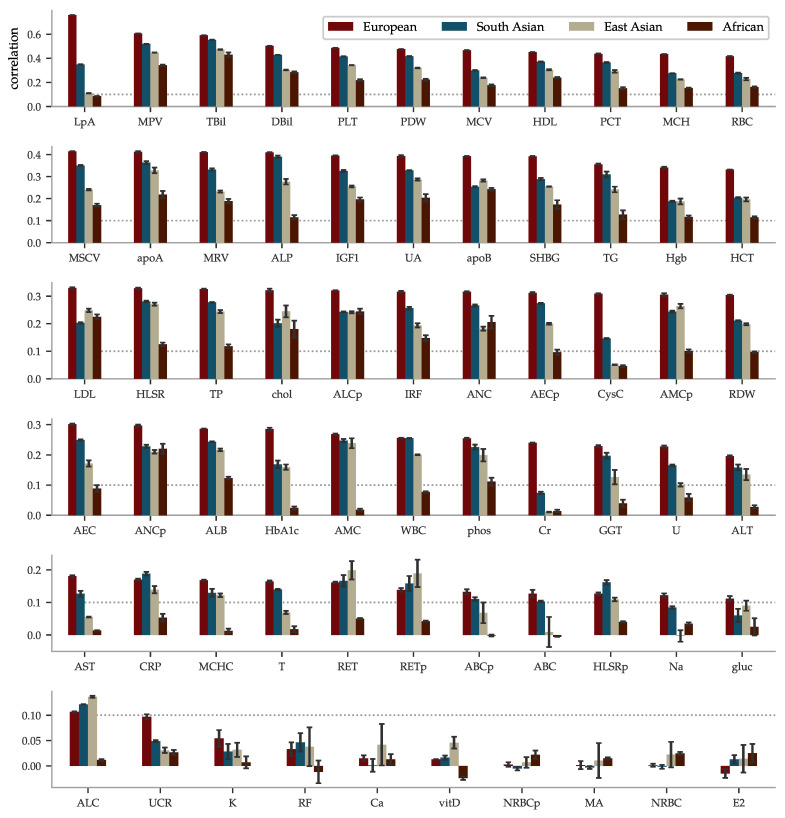
Correlations between PGS and phenotype vary from very strong to effectively zero, depending on the biomarker, and fall off with genetic distance from the training population. The mean of the PGS-phenotype correlation for evaluation sets are listed for all 65 biomarkers, ordered according to the results within Europeans—the ancestry for the training population. The error bars represent ± the standard deviation for 5 different predictors trained on slightly different training sets. The dotted line is there to aid graphical comparisons across the rows. The LASSO predictor of lipoprotein A achieves a correlation of 0.759 within European ancestry, which is the highest correlation for a polygenic trait we are aware of. The correlation fall-off for the other ancestries generally follows the order European > South Asian > East Asian > African. Note that the sample sizes for these ancestries are much smaller.

The performance of the PGS predictors ranges from the highest phenotype-PGS correlation for a polygenic predictor we are aware of to no predictive power whatsoever. We present the results in order of correlation within European ancestry in Figure 3. The best performing predictor is for lipoprotein A at a correlation of ∼0.76. This is not too surprising as lipoprotein A levels are well-known to be highly heritable [97,98,99,100], related to the LPA gene and other loci [101,102,103,104,105,106,107,108,109,110], and, thus, do not greatly vary by life style or environment. Lipoprotein A has long been studied because of its association with CAD, atherosclerotic risk, liver problems, metabolism, and even cancer. Further discussion can be found in the review [111]. Yet, it is a striking example of predictive power: previous PGS have found much lower correlations for other traits. For example, height (∼0.62 [55,112]), BMI (∼0.35 [54,112], ∼0.30 [15]), educational attainment (∼0.27 [55], ∼0.35 [113]), and heel bone density (∼0.45 [55], ∼0.42 [114]). After lipoprotein A, we find correlations almost evenly distributed within the correlation range 0.1 to 0.59 and a group of 7 almost uncorrelated biomarkers at the bottom. In the same Figure 3, we have included the performance within the non-European ancestries. Being trained on European ancestry only, the predictors suffer the now familiar [90,91] fall-off pattern according to genetic distance, with performance generally being successively worse for South-Asian, East-Asian, and African ancestries.

To account for population stratification we took a two-fold approach: comparing prediction with and without adjusting for a principal component analysis, and performing sibling analyses. We used the UKB provided principal components for each individual for this analysis. We performed a linear regression on phenotype using the top 20 principal components while excluding the testing set (siblings). To assess the impact of principal components on the PGS, we compared the correlation between (1) phenotype (*y*) and predicted phenotype ($y_{\mathrm{PCA}}$) from PCA (corr$\left(y,y_{\mathrm{PCA}}\right)$) (2) phenotype and PGS generated from training on the raw phenotype (corr(y,PGS)) (3) the correlation between phenotype and PGS generated from training on the residual phenotype (corr$\left(y,{\mathrm{PGS}}_{\mathrm{PCA}}\right)$). This was done for 5 out of the strongest performing predictors. The results are shown in Table 2 for the evaluation set of self-reported European individuals within sibling pairs.

From the results in Table 2 we can characterize the difference between training with and without PCA as the square root of the average difference squared

(2) $$\sqrt{\mathrm{Mean}\left[{\left(\mathrm{corr}\left(y,\mathrm{PGS}\right)-\mathrm{corr}\left(y,{\mathrm{PGS}}_{\mathrm{PCA}}\right)\right)}^{2}\right]}=0.00617\pm 0.00003\;,$$

or as an average percent difference

(3) $$\left|\frac{\mathrm{corr}\left(y,\mathrm{PGS}\right)-\mathrm{corr}\left(y,{\mathrm{PGS}}_{\mathrm{PCA}}\right)}{\mathrm{corr}(y,{\mathrm{PGS}}_{\mathrm{PCA}})}\right|=1.0\%\pm 0.4\%\;.$$

**Figure 4:**
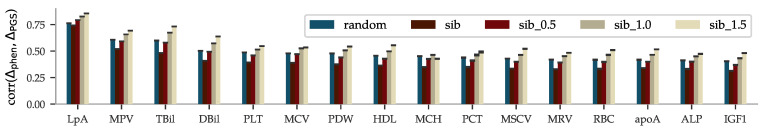
Sibling comparisons of correlation between difference in phenotype and difference in PGS, i.e., $\mathrm{corr}(\Delta_{\mathrm{phen}},\Delta_{\mathrm{PGS}})$, show that most of the correlation is also retained for pairs that share similar environmental backgrounds. UKBs∼40 k siblings of European ancestry were paired either randomly or as genetic siblings and were used as a test set. The correlations between the pairs’ differences in phenotype and their differences in PGS was then calculated for each biomarker, ordered above from strongest to weakest correlation. The error bars indicate ± the standard deviations for 5 predictors trained on slightly different training sets. The additional three bars 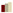 labeled sib 0.5, sib 1.0, and sib 1.5, are the results when restricting to siblings with phenotype differences larger than 0.5, 1, and 1.5 standard deviations, respectively. Two siblings are likely to have more similar environmental backgrounds than random pairs, affecting the similarity of late-life biomarker measurements independently from (direct) genetic effects. This could explain the decreased correlation for siblings as compared to random pairs. Yet, the remaining correlations are strong evidence that the predictors capture some direct genetic effects on the biomarkers. The comprehensive figure for all biomarkers can be found in the Supplementary Information.

The results from the sibling comparison can be seen in Figure 4. On average, there is a ∼26% drop in correlation when comparing differences within random pairs and differences within sibling pairs. The figure also shows that siblings that are separated by more than 0.5, 1.0, and 1.5 times the standard deviation in phenotype are predicted with increased correlation. The sibling comparisons for the other biomarkers can be found in the Supplementary Information.

#### 3.1.1. Genetic Architecture

**Figure 5:**
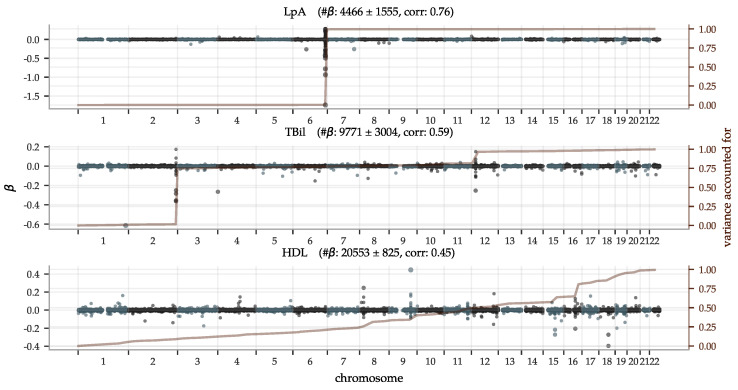
Manhattan plots of LASSO β—superimposed with the aggregate single SNP variance accounted for—show both highly localized, as well as widely polygenic architectures. The predictor for lipoprotein A is almost entirely determined by the well-known gene LPA in chromosome 6; the top 50 SNPs in this region account for ∼95% of the aggregate single SNP variance. In contrast, HDL has an almost uniform distribution of the variance accounted for across all the 22 autosomal chromosomes, despite some loci with high magnitude β-coefficients. (The difference being due to the MAF in Equation (1)). The most significant genetic loci are discussed further in the main text. The plot titles include the achieved PGS-phenotype correlation and mean number of non-zero β ± the standard deviation for the 5 predictors trained on each trait. Similar plots for all 65 biomarkers can be found in the Supplementary Information.

Polygenic predictors have shown to usually use information spread over the entire genome, even when enforcing sparsity [11,55,56,57]. In Figure 5, we illustrate the genetic architectures behind three of the top performing PGS predictors with Manhattan plots of the effect sizes β and the variance accounted for in Equation (1), accumulated across chromosomes 1–22 (the Supplementary Information contains figures for all biomarkers). It shows that both biomarkers with a few very strong loci and biomarkers with an evenly distributed dependence can be predicted well. Let us make a few remarks on the top 5 performing predictors (see Supplementary Information for the direct bilirubin and platelet count plots):

- The lipoprotein A predictor is as expected heavily dominated by the single locus on chromosome 6, the gene carrying its name LPA;
- The total bilirubin predictor is very similar to the one for direct bilirubin. GWASes have implicated many variants on all but chromosome 15 (according to a GWAS Catalog [115] trait search) but most have a very minor impact on our predictor. For example, Reference [116] reported a locus on chromosome 19 but although there are groups of moderately large β in this region, the entire chromosome 19 does not account for more than ∼1% of total variance in our predictors;
- GWASes for direct bilirubin in the literature [116,117] are generally dominated by variants in gene UGT1A on chromosome 2. The LASSO predictors pick these up too. In addition, there is another ∼17% variance accounted for by the locus at chromosome 12, also known [117]. Chromosomes 6 and 19 account for ∼1% variance each and have no generally listed loci. The $\beta_{i}$ with the largest magnitude corresponds to SNP rs908327 on chromosome 1. It has SNPs in linkage disequilibrium (LD) that have been linked to triglycerides [118] but not directly to bilirubin, to our knowledge. It has a very small MAF, however, and does not account for much variance;
- The predictor for platelet count is very polygenic with the variance accounted for almost evenly distributed across all 22 chromosomes. Chromosome 12 provides a small deviation from this pattern, accounting for ∼14% of the variance, partly due to a locus near one end;
- The predictor for HDL is also highly polygenic. Previous GWASes have recorded loci at all but chromosome 13, which has no large magnitude $\beta_{i}$ but still accounts for ∼1% of the total variance.

### 3.2. Predicting Disease Risk

The results for the disease risk predictors are divided into sections corresponding to the BMRS and gBMRS, respectively.

#### 3.2.1. BMRS: Predicting Case Status from Biomarkers

**Figure 6:**
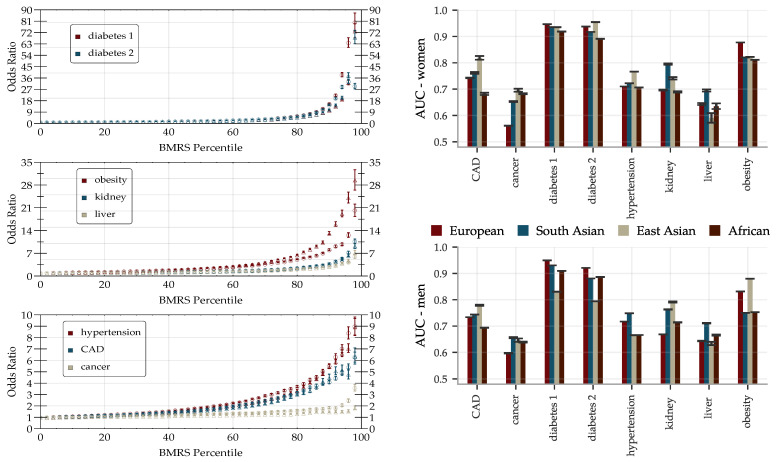
The predictive power of BMRS can single out high risk individuals with over 10× odds ratio for many traits, and AUCs > 0.7 for most traits including tests across ancestry. (**Left**): inclusive odds ratio (OR) plots for diabetes type 1/2, obesity, kidney problem, liver problem, hypertension, CAD, and any cancer trained and validated on the European population. Horizontal axis indicates individuals at that percentile *and above* in PRS. Marker ◯ is for predictors trained and validated on men and marker △ for predictors trained and validated on women. Error bars represent the standard error of the mean value with a contribution coming from computing the OR and a contribution from including 5 predictors. (**Right**): AUCs for BMRS predictors separately trained on men and women. All predictors are trained on the European population and then validated on European, South Asian, East Asian, and African populations. The error bars indicate the standard deviations for 5 different predictors and do not reflect the significant uncertainties arising from limited available statistics (sample sizes are listed in Supplementary Information).

The performance of the BMRS predictors was evaluated and are reported as AUCs and odds ratio plots in Figure 6. With training optimized for European ancestry on the evaluation sets, we regard the results for this ancestry as the main results and provide the performance in other ancestries for reference. The results vary with the condition. Within European ancestry, they range from an AUC of 0.53 (0.60) for cancer for women (men) up to ∼0.95 for diabetes type 1 (both sexes). As a comparison, we report below on an ASCVD predictor with an AUC of ∼0.76 which performs risk prediction, as well as or better than the American College of Cardiology ASCVD Risk Estimator. We discuss this in detail below in Section 4. The odds ratio plots show a wide range of results that also vary with condition. Figure 6 separates conditions into groups based on the odds ratios of the high risk outliers. The strength of the diabetes predictors is probably due to their use of blood biomarkers (e.g., HbA1c) which are standard diagnostic indicators for diabetes. That this standard diagnostic indicator is so highly ranked lends confidence to the results of the general methodology.

There are some differences in performance for men and women, most notably in cancer (possibly due to sex specific cancer variants). The differences are condition specific and viewed across all conditions the performance is similar. We delay a more detailed analysis of these differences to future study. The reported performance variations across the different ancestries are notably smaller and show less of a consistent pattern than what is the usual case for prediction from genetic information; this is expected since predicting from biomarkers stays on a higher biological level and does not involve issues such as LD patterns and tag SNPs, etc. Note, however, that these results are limited by the available statistics, see Supplementary Information for the case/control numbers for each ancestry.

**Figure 7:**
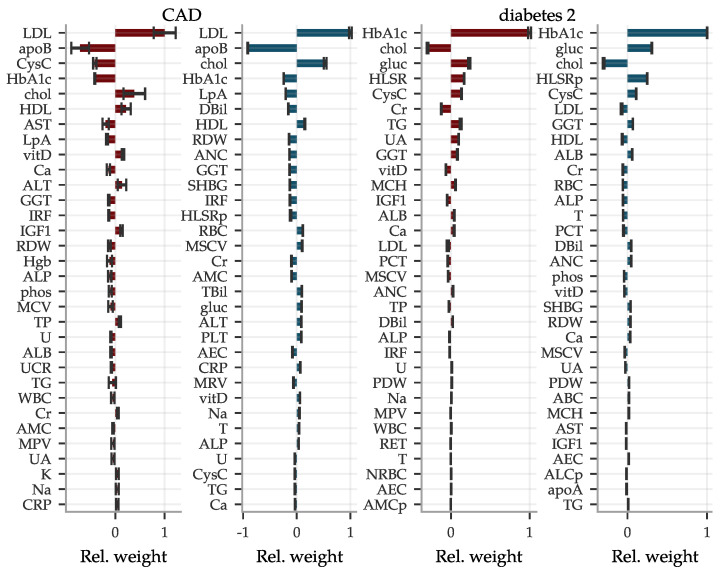
Predictors for phenotypes like CAD and type 2 diabetes from biomarkers are dominated by a top few inputs. Shown are relative weights (normalized with the largest magnitude) for the most important biomarkers within predictors for CAD and type 2 diabetes. 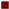 women and 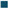 men while error bars indicate ± standard deviations from the mean of five predictors. The most impactful biomarkers are very well-known but we highlight cystatin C as surprisingly frequent among the moderately strong coefficients. Corresponding plots for all condition predictors are shown in the Supplementary Information.

In Figure 7, we also include two examples of the LASSO coefficients for CAD and type 2 diabetes. For CAD, we find mostly well-known biomarkers with the highest weight, such as LDL, apolipoprotein B, total cholesterol and HDL. However, for women cystatin C appears at fourth place, which to our knowledge is not often used in this context. Cystatin C also is the fifth most influential biomarker in the diabetes type 2 predictor for both sexes, while these predictors are dominated by the standard biomarker glycated haemoglobin. In fact, cystatin C is among the more important biomarkers for most of our predictors. Coefficients for all conditions are listed in the Supplementary Information.

We investigated the presence of non-linear effects for BMRS by extending the input features with all possible quadratic interactions among the seven most influential biomarkers for each condition. We saw no effect on the performance in either direction and conclude that the effects of the biomarkers on all the listed conditions appear to be linear to very good approximation.

Finally, to control for population stratification and confounding variables, we conducted a PCA comparison also for the predictors trained on biomarkers. We used the UK Biobank provided principal components for each individual for this analysis.

We performed a linear regression on each input biomarker phenotype, i.e., the age corrected and sex specifically z-scored phenotypes, using 20 principal components. The phenotypes were then further corrected by subtracting the predictions of the linear regressions and were again z-scored. These (age, sex, and) PCA corrected phenotypes were used as covariates when training a new set of LASSO predictors. The resulting relative AUC difference,

(4) $$\frac{\mathrm{AUC}-{\mathrm{AUC}}_{\mathrm{PCA}}}{{\mathrm{AUC}}_{\mathrm{PCA}}}\;,$$

is shown in Table 3. The largest effect was for liver disease at just over 1% whereas the rest of the phenotypes displayed a sub-percent change.

#### 3.2.2. gBMRS: Predicting Case Status from PGS of Biomarkers

**Figure 8:**
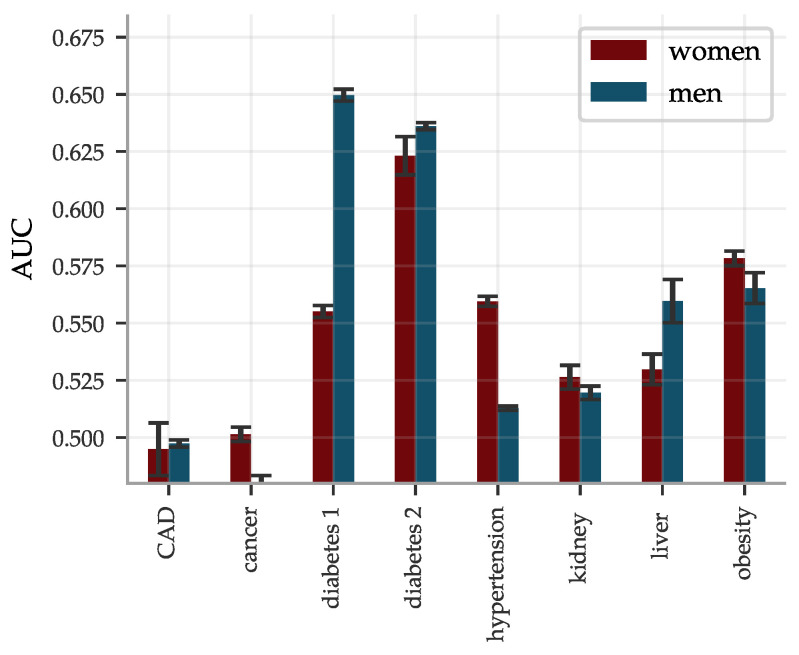
AUCs for gBMRS predictors drop significantly as compared to BMRS in Figure 6 and only the diabetes predictors reach par with other methods. The predictors were evaluated on 9016 (9606) white women (men) and the error bars indicate ± the standard deviation for 5 different predictors.

**Figure 9:**
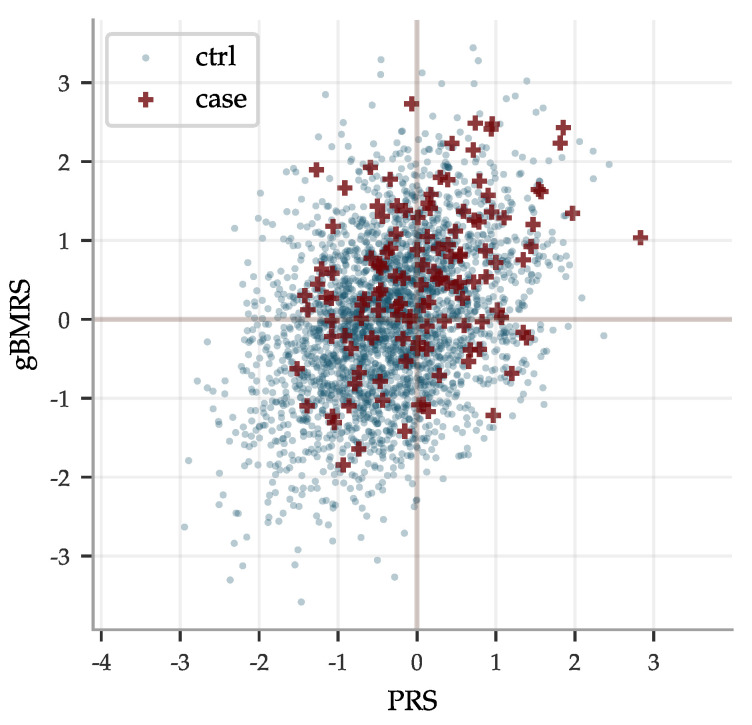
Risk scores predicted from SNPs PRS and from PGS of biomarkers gBMRS do not always agree, here exemplified by type 2 diabetes data for men. Both predictors predict case status directly from SNPs alone. Their outputs correlate ∼0.37 with a linear regression coefficient of ∼0.39. In the noise, they capture some complementary information: the sum of the risk scores achieves an AUC of ∼0.67 while the gBMRS and PRS predictors individually achieve AUCs of ∼0.63 and ∼0.64, respectively.

The concatenated predictors gBMRS suffer a significant drop in performance, as can be seen in Figure 8. The imprecise PGS-predictors introduce a lot of noise and, exacerbated further by the uncertainty in the BMRS predictors, the concatenation does in general not lead to meaningful predictions. A notable exception are the diabetes predictors. The combination of reasonably correlated PGS for the most important biomarkers and the exceptionally high AUCs for these predictors lead to an average AUC of ∼0.63 for the type 2 diabetes gBMRS predictor. This is comparable to what we have achieved in the past by training SNP-based LASSO directly on type 2 diabetes status (AUC ∼0.64) [11]. Furthermore, the two different types of predictors gBMRS and PRS capture somewhat complementary information, as shown in Figure 9. The sum of the two types of risk scores reaches an AUC of ∼0.67. It is unclear why the use of biomarkers as an intermediate step adds additional information relative to training directly with SNPs as features and case status as the phenotype. We leave this as an interesting topic for future research.

**Figure 10:**
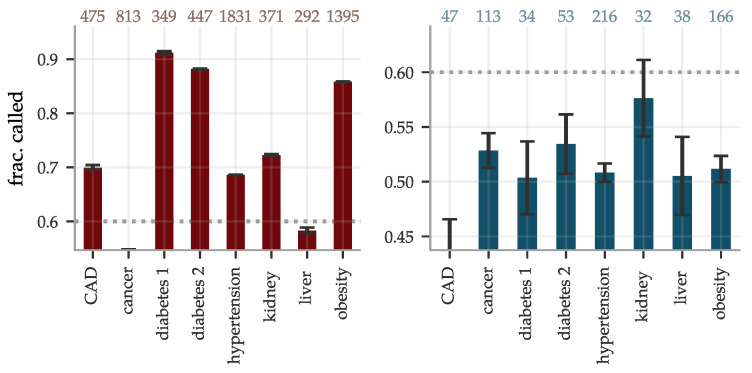
The fractions of sibling pairs with precisely one case and one control called correctly are generally high for 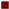 BMRS but not much better than chance when predicting from genotypes using 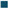 gBMRS. The pairs were considered correctly called if the PRS was higher for the affected sibling, without any restriction on the size of the separation. Number of included sibling pairs differed for the two types of predictors and are listed at the top. The error bars indicate ± the standard deviation for five different predictors for BMRS and for 5×5 concatenation combinations of predictors in the gBMRS.

The sibling evaluation of the disease risk predictors, as described in Section 2.2, is reported in Figure 10. The fraction of sibling pairs with one case and one control called correctly ranged from pure chance for cancer and liver problems, while reaching ∼0.9 for diabetes type 1 and 2, using the BMRS predictors. The accuracy dropped significantly for the gBMRS predictors, as expected; no predictor of this type reached a correctly called fraction above 0.6.

### 3.3. Comparison with ASCVD Risk Estimator

**Figure 11:**
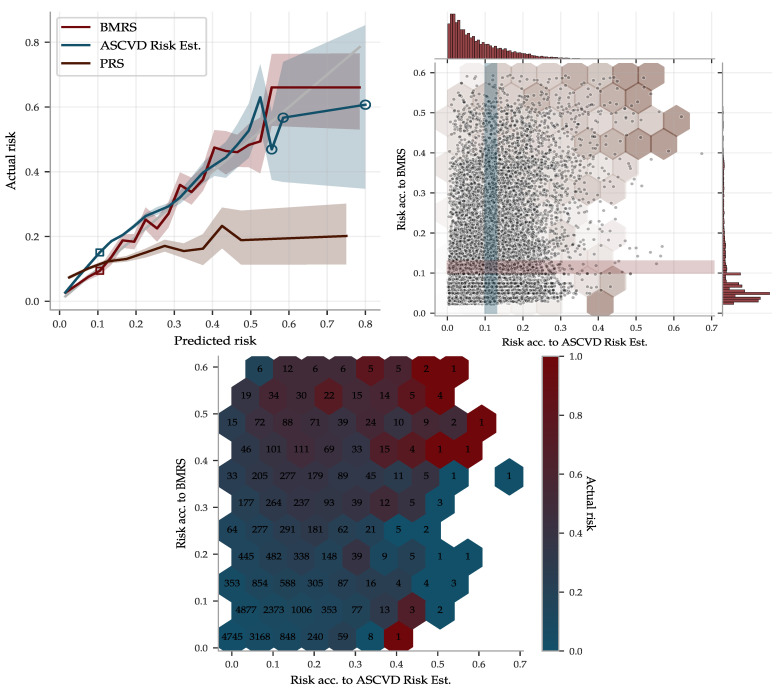
The ASCVD BMRS and the ASCVD Risk Estimator both make accurate risk predictions but with partially complementary information. (**Upper left**): Predicted risk by BMRS, the ASCVD Risk Estimator and a PRS predictor were binned and compared to the actual disease prevalence within each bin. The gray 1:1 line indicates perfect prediction. Shaded regions are 95% confidence intervals obtained from 100 fold bootstrap estimates of the prevalence in each bin (hollow circles indicate bin size <50 samples). The ASCVD Risk Estimator was applied to 340k UKB samples while the others were applied to an evaluation set of 28k samples, all of European ancestry. (**Upper right**) shows a scatter plot and distributions of the risk predicted by BMRS versus the risk predicted by the ASCVD Risk Estimator for the 28k Europeans in the evaluation set. The BMRS distribution has a longer tail of high predicted risk, providing the tighter confidence interval in this region. The left plot y-axis is the actual prevalence within the horizontal and vertical cross-sections, as illustrated with the shaded bands corresponding to the hollow squares to the left. Notably, both predictors perform well despite the differences in assigned stratification. The hexagons are an overlay of the (**lower center**) heat map of actual risk within each bin (numbers are bin sizes). Both high risk edges have varying actual prevalence but with a very strong enrichment when the two predictors agree.

To illustrate the performance of the BMRS predictor for ASCVD and to compare it with the ASCVD Risk Estimator, we used the risk percentage output, as described in Section 2.3. The ASCVD Risk Estimator was built using American cohorts of separately European and African ancestry. Due to the similarities with the UKB population, we deemed it could be applied somewhat fairly to the entire UKB, whereas we used the withheld evaluation set of ∼40 k of European ancestry for the BMRS predictor. The result is shown in Figure 11, in which the predicted risks were binned and the actual disease prevalence within each bin was calculated, labeled “Actual risk”. Both predictors give very accurate risk estimates, with increasing uncertainty for individuals with high predicted risk. However, although they do assign correct risk estimates for bins taken as a whole, they do not always agree on who is at low versus high risk. The scatter plot in Figure 11 shows their individual distributions and occasional disagreements. Their partially complementary predictions are further highlighted in the risk heat map in Figure 11 and utilized below in a combined predictor.

#### Combination of Predictor from Biomarkers and the ASCVD Risk Estimator

**Figure 12:**
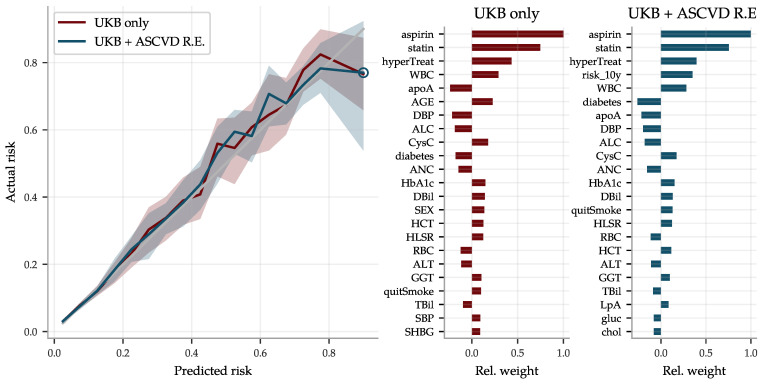
The risk prediction using both 62 biomarkers and all the ASCVD Risk Estimator input improves performance as compared to Figure 11, in particular for high risk individuals, and is very good all the way up to risk levels of 80%. The figure compares two predictors: 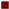 a combined ASCVD predictor using all 62 biomarkers plus all the input fields (age, sex, etc.) used by the ASCVD Risk Estimator, using UKB data only, and 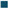 a predictor using the same input plus the ASCVD Risk Estimator *output*, labeled UKB + ASCVD R.E. The latter does not perform notably better, although the ASCVD Risk Estimator output “risk_10y” corresponds to the fourth strongest coefficient. Both perform better than both the BMRS and ASCVD Risk Estimator individually, confirming their complementary nature shown in the heat map in Figure 11. The shaded areas in the left panel again indicate 95% confidence intervals obtained by 100 fold bootstrap calculations of the actual prevalence in each risk bin. Figures with all coefficients can be found in the Supplementary Information.

Since the ASCVD Risk Estimator and the BMRS predictor use different input and give complementary predictions, we combined them into a a very reliable risk predictor, superseding both the former. The risk estimates are compared with actual disease prevalence in Figure 12 for two versions of the combined predictor: (1) a linear regression on the biomarkers and all of the input going into the ASCVD Risk Estimator, and (2) a similar regression but also including the *output* of the ASCVD Risk Estimator. Their top coefficients are listed in the same figure.

## 4. Discussion

UK Biobank data include about 500 k individuals, for each of whom the following are recorded: SNP genotype, biomarker (blood, urine) test results, and case status for most common disease conditions. We have explored the pattern of correlations between these three distinct data types using machine learning.

We have shown that SNPs can be used to predict quantitative values of biomarkers by training new polygenic scores (PGS) for biomarker prediction. We note that the day to day fluctuation of these biomarker levels suppresses the quality of prediction. A more stable phenotype (e.g., average value of biomarker measured on multiple occasions) would probably be even better predicted from SNPs alone.

As is typical for current genomic predictors, we find that predictive power falls off significantly with genetic distance from the (European) training population. This highlights the importance of increasing ancestry diversity in genetic data collection. As genetic predictors begin to find clinical applications, lack of diversity can exacerbate healthcare inequalities [89,119] (a larger list of associated ethical issues is highlighted in [54]).

We showed that biomarkers can be used as input to predict common disease risk. Some of these BMRS predictors (e.g., ASCVD, diabetes) are very strong and may even surpass risk predictors in widespread clinical use. The combined predictor trained using both biomarkers and ASCVD Risk Estimator inputs clearly outperforms the latter in our comparison, at least for individuals at very high risk. It should be emphasized that we did not have access to sufficient clinical data necessary for an analysis equivalent to the careful evidence review or statistical analysis that underlie the ASCVD Risk Estimator [62]. For example, the ASCVD Risk Estimator takes into account cost–benefit of the number of inputs, whereas we used all available UKB data irrespective of practical costs and clinical availability. Additionally, our comparison did not take into account the time of diagnosis relative to biomarker measurement. Nevertheless we consider our exploratory analysis to be indicative of the power of BMRS predictors, justifying further work making use of additional datasets.

In the case of kidney disease, there are various predictive scores that are used by clinicians that rely on biomarkers. (Current “Kidney Disease: Improving Global Outcomes” (KDIGO) guidelines can be found in [120].) The traditional score was the Cockroft-Gault equation [121] which takes into account serum creatinine, age, sex, and weight, and uses them to estimate creatinine clearance. More modern approaches use similar inputs, but are designed to estimate glomerular filtration rate (eGFR). The first to do this was the Modification of Diet in Renal Disease (MDRD) study equation [122]. The most recent approach is the Chronic Kidney Disease Epidemiology Collaboration (CKD-EPI) equations [123] which has been shown to be a better indicator of eGFR than MDRD (e.g., [124]). Both of these used serum creatinine as an input biomarker. The most recent version of the CKD-EPI equation also uses cystatin C as an additional input biomarker [125]. This work interestingly finds cystatin C to be *the* most important biomarker in relation to kidney disease, with creatinine the second most important.

For liver disease, the classic biomarker based score is the Child-Pugh score [126]. This uses a combination of serum bilirubin, albumin, prothrombin time, international normalized ratio (INR), and other diagnostic information to judge liver function. A more modern biomarker based approach is the model for end stage liver disease (MELD) [127] and many enhanced MELD scores that have followed. MELD based scores involve bilirubin, creatinine, INR, and other diagnostic information. Additionally, aminotransferases (ALT and AST) are routinely used to diagnose liver function. The most surprising results in this work are the high importance of cystatin C, gamma glutamyltransferase, and SHBG in predicting liver disease—as these are not part of the American Association for the Study of Liver Diseases (AASLD) [128] guidelines for diagnosing acute liver disease [129].

In future work it would be interesting to compare the existing liver and kidney risk scores to the new predictors we trained here. This analysis would be analogous to the ASCVD Risk Estimator comparison to the BMRS predictor discussed above.

We note that BMRS prediction quality does not exhibit the pattern of fall-off with genetic distance as previously found with genomic predictors. (Previous GWAS and PGS studies generally see a fall off behavior, but there are occasional exceptions, e.g., [130].) For example, CAD and ASCVD predictors work well in all major ancestry groups despite using a European training sample. Further investigation is needed.

We studied concatenated predictor functions, which map SNPs to biomarkers to risk. In general, there were significant declines in performance. The magnitudes of these declines were perhaps expected for correlation chains of generic, high dimensional, vectors with similar pairwise correlations. Of the gBMRS predictors, only the type 2 diabetes predictor performs well: AUC of ∼0.63. This is in fact comparable to what we have achieved in the past by training SNP-based LASSO directly on type 2 diabetes status. Furthermore, the two different types of predictors gBMRS and PRS capture somewhat complementary information, as shown in Figure 9. The sum of the two types of risk scores reaches an AUC of ∼0.67. It is unclear why the use of biomarkers as an intermediate step adds additional information relative to training directly with SNPs as features and case status as the phenotype. We leave this as an interesting topic for future research. References [131,132,133,134,135,136] are cited in the Supplementary Materials.

## Figures and Tables

**Table 1:** List of all studied blood and urine markers with abbreviations.

Abbr.	Full Name	Abbr.	Full Name	Abbr.	Full Name
ABC	Basophill count	E2	Oestradiol	NRBC	Nucleated red blood cell count
ABCp	Basophill percentage	GGT	Gamma glutamyltransferase	NRBCp	Nucleated red blood cell percentage
AEC	Eosinophill count	gluc	Glucose	PCT	Platelet crit
AECp	Eosinophill percentage	HbA1c	Glycated haemoglobin (HbA1c)	PDW	Platelet distribution width
ALB	Albumin	HCT	Haematocrit percentage	phos	Phosphate
ALC	Lymphocyte count	HDL	HDL cholesterol	PLT	Platelet count
ALCp	Lymphocyte percentage	Hgb	Haemoglobin concentration	RBC	Red blood cell (erythrocyte) count
ALP	Alkaline phosphatase	HLSR	High light scatter reticulocyte count	RDW	Red blood cell (erythrocyte) distribution width
ALT	Alanine aminotransferase	HLSRp	High light scatter reticulocyte percentage	RET	Reticulocyte count
AMC	Monocyte count	IGF1	IGF-1	RETp	Reticulocyte percentage
AMCp	Monocyte percentage	IRF	Immature reticulocyte fraction	RF	Rheumatoid factor
ANC	Neutrophill count	K	Potassium in urine	SHBG	SHBG
ANCp	Neutrophill percentage	LDL	LDL direct	T	Testosterone
apoA	Apolipoprotein A	LpA	Lipoprotein A	TBil	Total bilirubin
apoB	Apolipoprotein B	MA	Microalbumin in urine	TG	Triglycerides
AST	Aspartate aminotransferase	MCH	Mean corpuscular haemoglobin	TP	Total protein
Ca	Calcium	MCHC	Mean corpuscular haemoglobin concentration	U	Urea
chol	Cholesterol	MCV	Mean corpuscular volume	UA	Urate
Cr	Creatinine	MPV	Mean platelet (thrombocyte) volume	UCR	Creatinine (enzymatic) in urine
CRP	C-reactive protein	MRV	Mean reticulocyte volume	vitD	Vitamin D
CysC	Cystatin C	MSCV	Mean sphered cell volume	WBC	White blood cell (leukocyte) count
DBil	Direct bilirubin	Na	Sodium in urine		

**Table 2:** Including Principal Component effect on the PGS predictors has negligible effect. Results are for self-reported Europeans within the sibling set.

Abbr.	corr$\left(y,y_{\mathrm{PCA}}\right)$	corr(y,PGS) ± std	corr$\left(y,{\mathrm{PGS}}_{\mathrm{PCA}}\right)$ ± std
HDL	0.0128	0.4514 ± 0.0005	0.4574 ± 0.0002
LpA	0.0193	0.7591 ± 0.0003	0.7517 ± 0.0001
TBil	0.0338	0.5906 ± 0.0003	0.5838 ± 0.0001
MCV	0.0824	0.4673 ± 0.0004	0.4600 ± 0.0079
MPV	0.0290	0.6064 ± 0.0005	0.6061 ± 0.0008

**Table 3:** Including Principal Component effect on the BMRS predictors has negligible impact on the AUCs. Shown is the relative AUC as computed in Equation (4) for self-reported Europeans within the sibling set, using the mean relative difference for 5 numbered predictors ± the standard deviation.

	rel. AUC (women)	rel. AUC (men)
CAD	0.0027 ± 0.0160	−0.0088 ± 0.0063
cancer	−0.0060 ± 0.0089	0.0049 ± 0.0051
diabetes 1	−0.0068 ± 0.0026	−0.0006 ± 0.0042
diabetes 2	−0.0002 ± 0.0037	−0.0014 ± 0.0025
hypertension	−0.0026 ± 0.0029	0.0005 ± 0.0024
kidney	−0.0033 ± 0.0124	−0.0074 ± 0.0161
liver	−0.0108 ± 0.0113	−0.0370 ± 0.0072
obesity	−0.0004 ± 0.0011	0.0030 ± 0.0017

## Data Availability

The predictors described in the paper are available to other researchers upon request. A Jupyter notebook with most of the code (but no data) used for the analysis in this paper can be found on the group’s GitHub account: www.github.com/MSU-Hsu-Lab (accessed on 23 June 2021).

## Supplementary Materials

The following are available online at https://www.mdpi.com/article/10.3390/genes12070991/s1, Supplementary Information, Figures S1–S3: Phenotype distributions for Europeans (women/men), Figure S4: Phenotype dependence on age, Figure S5: Calibration of PGS predictors, Figure S6–S16: Scatter and calibration plots for PGS, Figure S17: Sibling comparison of PGS correlations, Figures S18–S26: PGS predictor Manhattan plots and variance accounted for, Figure S27: Distributions of time differences between condition onset and time of first biomarker measurement, Figure S28: Distributions of time differences between ASCVD onset and time of first biomarker measurement, Figures S29–S31: Coefficient sizes for BMRS predictors, Figure S32: Coefficient sizes for the different ASCVD predictors, Figures S33–S34: QQ-plots and distribution histograms for the BMRS predictors, Table S1: Calibration of PGS predictors, Table S2: PGS correlations and sample sizes, Table S3: Numbers of pairs in PGS sibling analysis, Table S4: Sample sizes and AUCs for the BMRS predictors, Table S5: Sample sizes and AUCs for the gBMRS predictors.
