# Supplementary material for: Machine Learning Prediction of Biomarkers from SNPs and of Disease Risk from Biomarkers in the UK Biobank
Source: Genes (Basel). 2021 Jun 29;12(7):991. doi: 10.3390/genes12070991 (PMC8308062; doi:10.3390/genes12070991)
Supplement: Supplementary file 1 [file genes-12-00991-s001.zip › genes-1185109-supplementary.pdf]

Supplementary Information — Machine Learning Prediction of Blood and Urine Biomarkers from Genotype and of Disease Risk from Biomarkers in the UK Biobank

Erik Widen Timothy G. Raben Louis Lello Stephen D.H. Hsu

June 27, 2021

S0 Contents

S1 Methods **S1**
S1.1 LASSO S1

S2 Phenotype Descriptions **S2**
S2.1 Phenotype distribution by sex for European ancestry S2
S2.2 Phenotype dependence on age S6

S3 Phenotype Prediction **S8**
S3.1 Calibration S8
S3.2 PGS correlations S8
S3.3 Sibling evaluation S22
S3.4 Genetic Architectures S23

S4 Disease Prediction **S33**
S4.1 Disease condition definitions S33
S4.2 Time between condition onset and biomarker measurement S35
S4.3 Input data to the ASCVD Risk Estimator S35
S4.4 Risk conversion for ASCVD predictors S35
S4.5 Coefficient sizes S37
S4.6 AUCs and sample sizes S42
S4.7 Risk score distributions S44

S1 Methods

Here we expand upon the sparse prediction methods described in the main text. Things like how error bars were calculated, how we used python and cross checked things with julia, what type of computer cluster we ran on, etc.

S1.1 LASSO

Implementing LASSO amounts to finding the set of p coefficients β that minimizes the objective function

$$\mathcal{O} = \frac{1}{2}\|\hat{y} - X\beta\|^2_{L_2} + \lambda|\beta|_{L_1}\,, \tag{1}$$

heavily X is the $(N \times p)$ genotype matrix with the number of minor alleles at each SNP position for each sample and λ is a hyperparameter controlling the amount of regularization imposed on β. $\hat{y}$ is the N-vector of phenotype values for each sample which is either a raw phenotype, y, or adjusted via linear regression for age, sex, other covariates, or environmental factors (E), i.e. $\hat{y} = y - \mu - EW$, for regression coefficients μ and W. Applying the predictor to a genotype x produces the Polygenetic Score (PGS) as output,

$$\text{PGS} = x \cdot \beta \,. \tag{2}$$

The LASSO objective function (1) favors sparsity, as controlled by λ, making it suitable for genetic prediction [131]. A well-chosen λ allows the predictor to focus on the comparably few relevant SNPs among the $\sim$ 50k used as input as selected by GWAS p-value.

For this work, LASSO was implemented using a standard package of Python3 [132]: Scikit-Learn [133]. Additionally, we used a custom implementation of LASSO, developed in Julia, as a cross check. Further details about this custom implementation can be found in [11,57,58]. All code was run on the Michigan State University High Performance Computing Cluster (HPCC). With parallelization, predictors can be trained on $\sim$ 300k-500k samples in $\sim$ 12-36 hours. Genotype input, X, was prepared using PLINK[134,135] and read with PySNPTools [136].

Within a population we can classify how much predicted variance there is as

$$\begin{aligned} \text{var}\,(x \cdot \beta) &= \sum_i \left(\text{var}\,(x_i\beta_i) + 2\sum_{j<i} \text{cov}\,(x_i\beta_i, x_j\beta_j) \right) \\ &= \sum_i \left(2\beta_i^2(1-f_i)f_i + 2\sum_{j<i} \text{cov}\,(x_i\beta_i, x_j\beta_j) \right) \,. \end{aligned} \tag{3}$$

The variance accounted for *from single SNPs alone* can be described as

$$\sum_i 2\beta_i^2(1-f_i)f_i \,. \tag{4}$$

This is equivalent to the *total* variance of the predictor when correlations among the SNPs are small. Sparse prediction methods generally suppress activating SNPs which are highly correlated, reducing the impact of the neglected covariance. Further details can be found in [57].

S2 Phenotype Descriptions

The research underlying this paper was conducted over the course of two data updates to a UKB application. The first dataset was acquired from UKB in April 2019 and was used for all the PGS research. The second, mostly overlapping, dataset was acquired in November 2020 and was used for the disease prediction research. The phenotype availability differed for these datasets why the sample sizes are not the same for the PGS-related results as for the BMRS results.

S2.1 Phenotype distribution by sex for European ancestry

Figure S1 to **Figure S3** show phenotype distributions for both females and males of European ancestry with the number of samples being used for the PGS training shown in the upper corner of each plot.

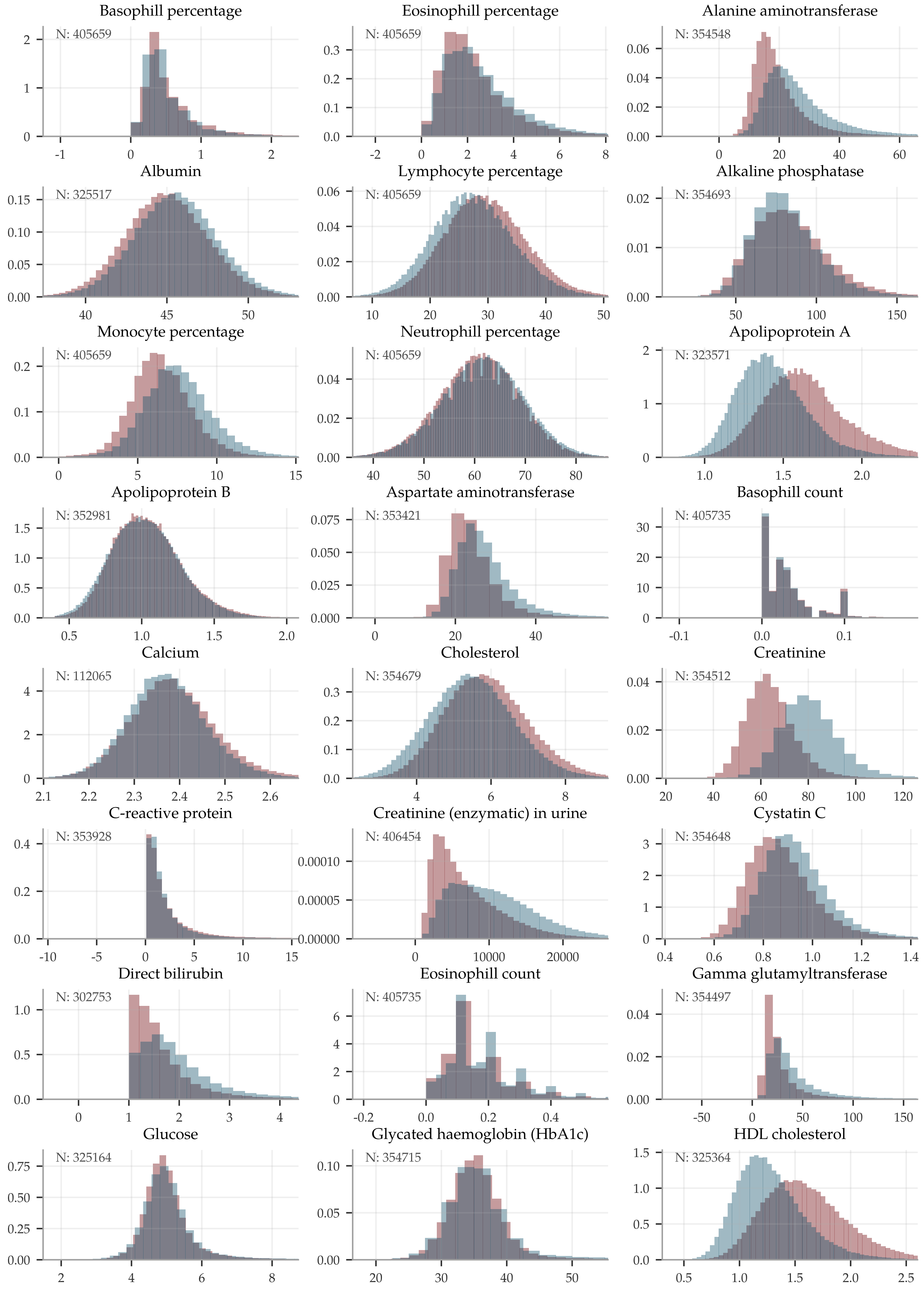

Figure S1: Distribution plots (1 of 3) for European ■ men and ■ women of the raw biomarker measurements. The x-axis are cropping some of the longer tails.

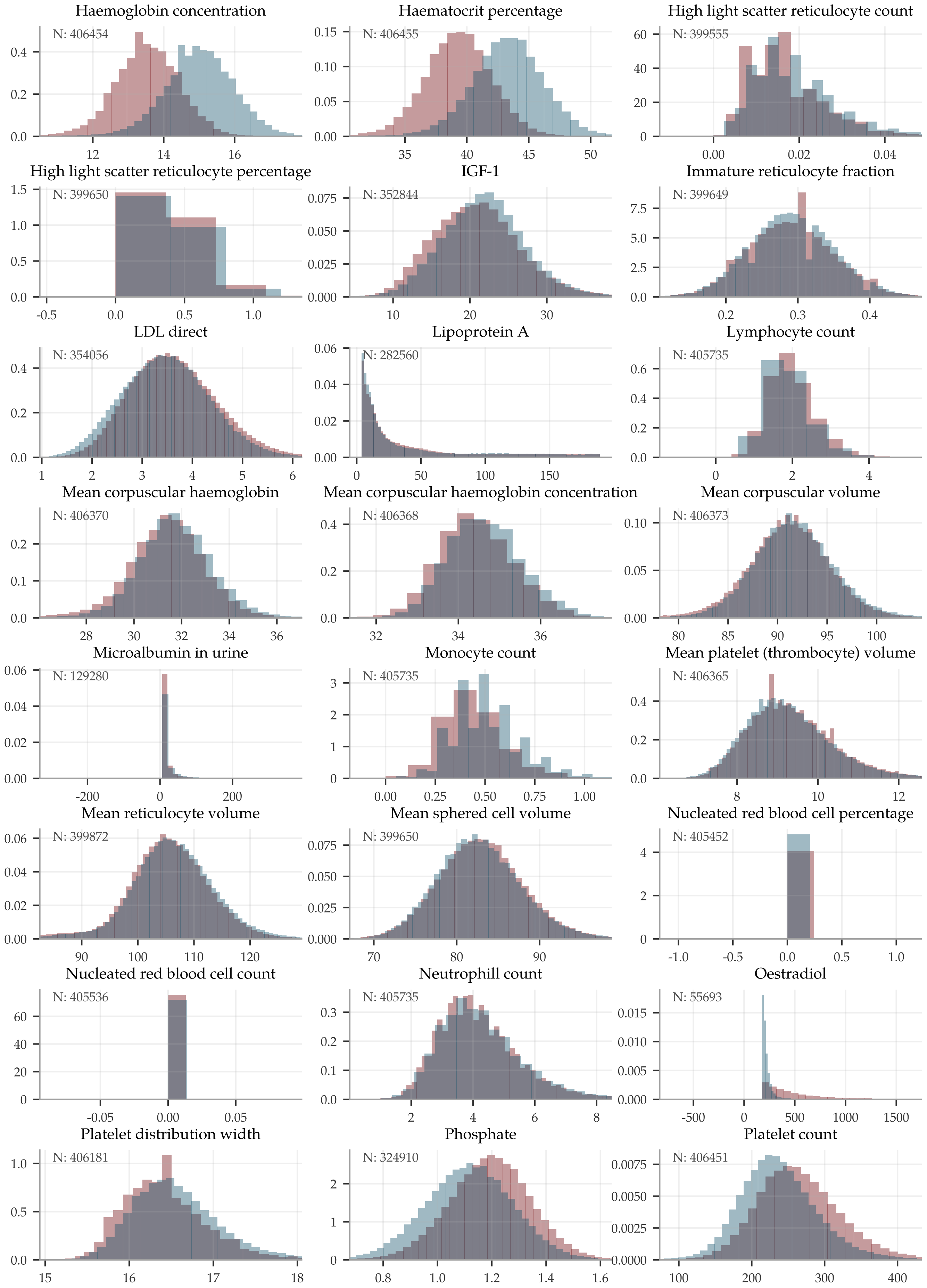

Figure S2: Distribution plots (2 of 3) for European ■ men and ■ women of the raw biomarker measurements. The x-axis are cropping some of the longer tails.

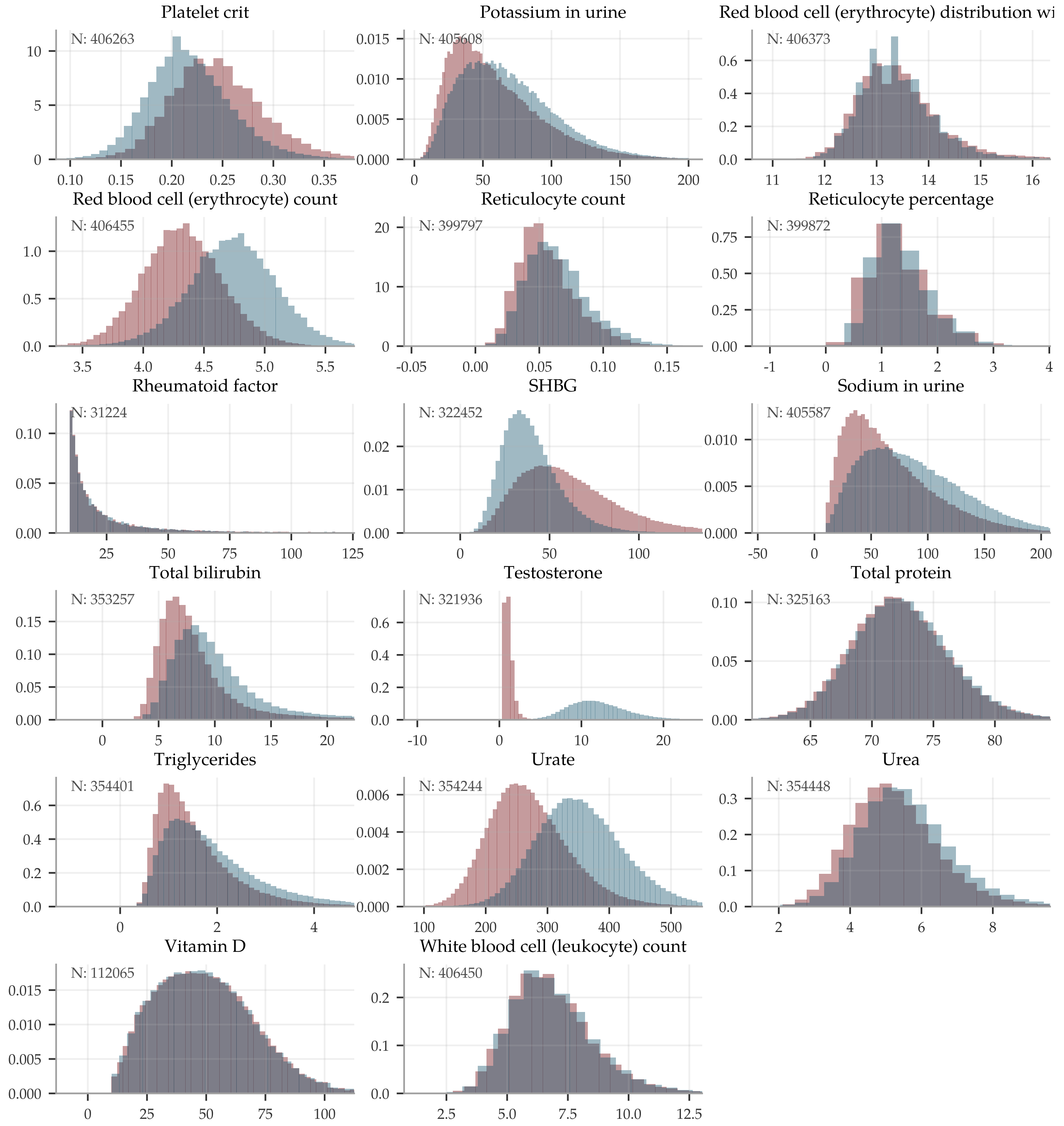

Figure S3: Distribution plots (3 of 3) for European men and women of the raw biomarker measurements. The x-axis are cropping some of the longer tails.

S2.2 Phenotype dependence on age

All individuals of European ancestry where binned by age at biomarker measurement and the normalized means in each bin are showed in **Figure S4**.

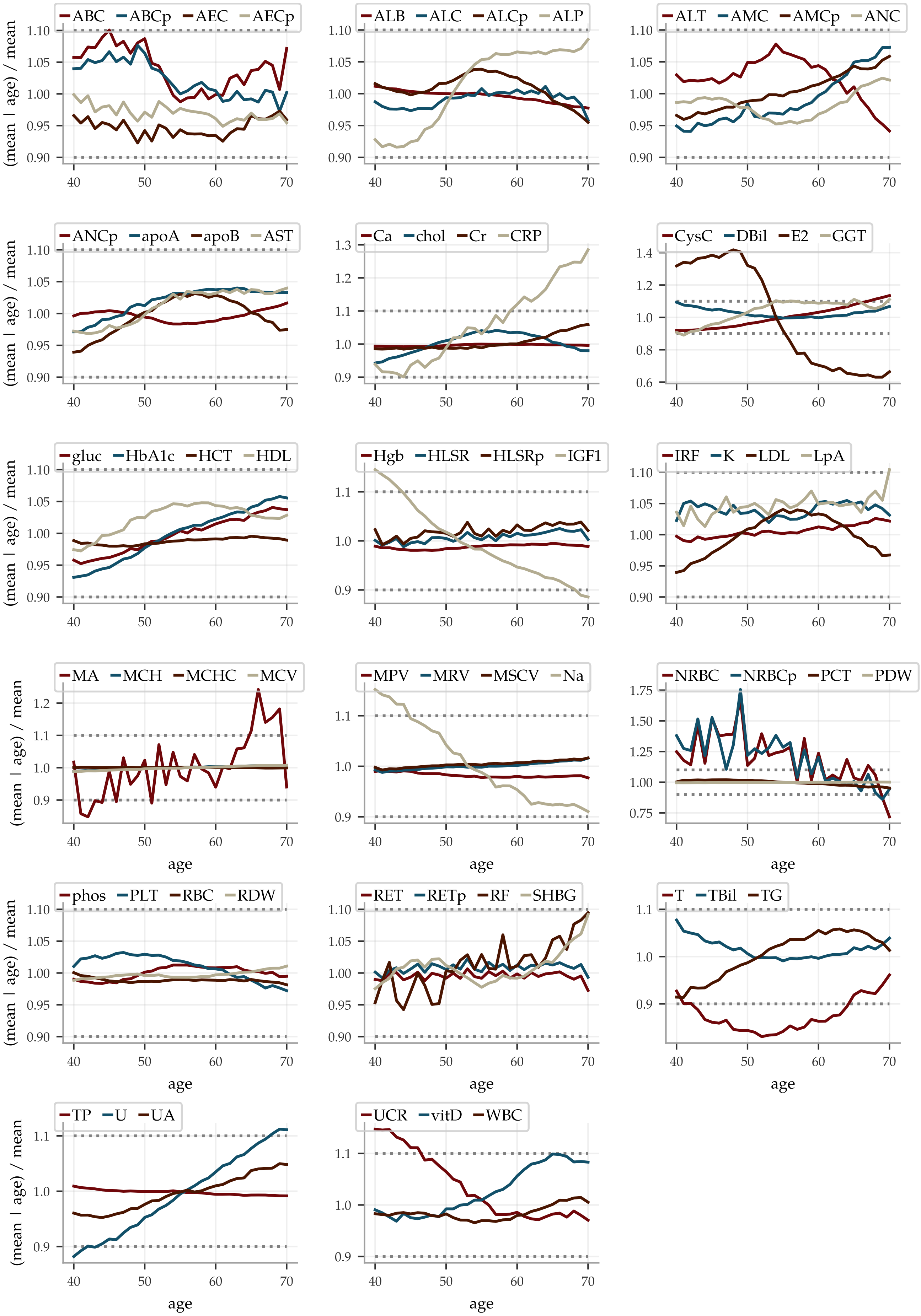

Figure S4: Age dependence of each of the biomarkers. The binned means of all Europeans of the same age are shown, normalized with the overall mean.

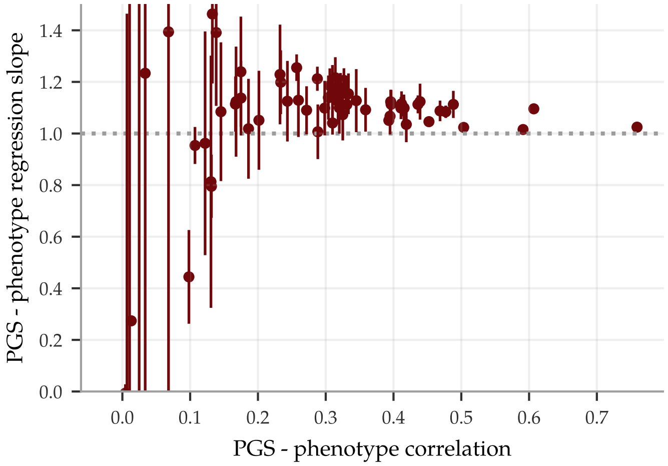

Figure S5: Calibration of PGS predictors is poor for predictors with low correlation and stabilizes somewhat for those predictors with correlations over 0.3. The error bars indicate the standard deviation for the five predictor for each trait. The numbers are shown in **Table S1**.

Abbr.	Slope ± std	Abbr.	Slope ± std	Abbr.	Slope ± std	Abbr.	Slope ± std	Abbr.	Slope ± std
ABC	1.46 ± 0.27	apoA	1.10 ± 0.05	HCT	1.15 ± 0.08	MCV	1.09 ± 0.04	RET	1.12 ± 0.21
ABCp	1.39 ± 0.28	apoB	1.05 ± 0.02	HDL	1.05 ± 0.02	MPV	1.10 ± 0.01	RETp	1.08 ± 0.27
AEC	1.14 ± 0.09	AST	1.02 ± 0.19	Hgb	1.13 ± 0.12	MRV	1.12 ± 0.05	RF	1.23 ± 2.30
AECp	1.22 ± 0.08	Ca	5.68 ± 10.35	HLSR	1.16 ± 0.05	MSCV	1.10 ± 0.04	SHBG	1.07 ± 0.07
ALB	1.21 ± 0.05	chol	1.07 ± 0.10	HLSRp	0.80 ± 0.12	Na	0.81 ± 0.49	T	1.11 ± 0.11
ALC	0.95 ± 0.07	Cr	1.13 ± 0.16	IGF1	1.11 ± 0.06	NRBC	-0.01 ± 0.03	TBil	1.02 ± 0.01
ALCp	1.18 ± 0.05	CRP	1.14 ± 0.14	IRF	1.10 ± 0.10	NRBCp	-1.87 ± 3.34	TG	1.09 ± 0.08
ALP	1.10 ± 0.04	CysC	1.04 ± 0.04	K	1.39 ± 1.56	PCT	1.12 ± 0.07	TP	1.21 ± 0.05
ALT	1.05 ± 0.19	DBil	1.02 ± 0.00	LDL	1.11 ± 0.03	PDW	1.08 ± 0.02	U	1.23 ± 0.19
AMC	1.09 ± 0.09	E2	-0.05 ± 0.03	LpA	1.02 ± 0.00	phos	1.13 ± 0.14	UA	1.12 ± 0.05
AMCp	1.16 ± 0.11	GGT	1.20 ± 0.12	MA	N/A	PLT	1.11 ± 0.05	UCR	0.44 ± 0.18
ANC	1.12 ± 0.09	gluc	0.96 ± 0.43	MCH	1.11 ± 0.03	RBC	1.04 ± 0.07	vitD	0.27 ± 0.01
ANCp	1.10 ± 0.11	HbA1c	1.01 ± 0.11	MCHC	1.24 ± 0.21	RDW	1.19 ± 0.07	WBC	1.26 ± 0.05

Table S1: Calibration of the PGS predictors.

S3 Phenotype Prediction

S3.1 Calibration

The calibration, i.e. the slope of a linear regression of the PGS predictors, varied: the poorly performing predictors clearly failed the calibration tests while all PGS with a correlation above 0.3 fared better. The calibration for each biomarker is plotted in **Figure S5** against the PGS-phenotype correlation and the numerical values are listed in **Table S1**.

S3.2 PGS correlations

The numerical values for the PGS-phenotype correlations are listed in **Table S2** as means ± the standard deviations for 5 predictors trained on each biomarker.

Table S2: PGS correlations for both sexes broken down according to ancestry. N denotes evaluation set sizes and training/evaluation set sizes in the case of European ancestry.

	European		South Asian		East Asian		African	
Abbr.	N	Corr	N	Corr	N	Corr	N	Corr
ABC	405735/38885	0.127 ± 0.011	9070	0.103 ± 0.003	1453	0.009 ± 0.046	7221	-0.006 ± 0.000
ABCp	405659/38885	0.132 ± 0.008	9070	0.110 ± 0.006	1453	0.068 ± 0.032	7221	-0.002 ± 0.003
AEC	405735/38885	0.302 ± 0.002	9070	0.250 ± 0.002	1453	0.172 ± 0.010	7221	0.089 ± 0.011
AECp	405659/38885	0.312 ± 0.003	9070	0.274 ± 0.002	1453	0.200 ± 0.003	7221	0.097 ± 0.009
ALB	325517/35240	0.287 ± 0.001	8217	0.244 ± 0.001	1296	0.217 ± 0.004	6632	0.123 ± 0.004

ALC	405735/38885	0.107 ± 0.001	9070	0.121 ± 0.000	1453	0.136 ± 0.002	7221	0.012 ± 0.002
ALCp	405659/38885	0.321 ± 0.001	9070	0.243 ± 0.002	1453	0.242 ± 0.004	7221	0.245 ± 0.010
ALP	354693/38326	0.409 ± 0.001	8989	0.390 ± 0.005	1422	0.277 ± 0.013	7170	0.115 ± 0.010
ALT	354548/38320	0.196 ± 0.003	8973	0.159 ± 0.010	1421	0.135 ± 0.019	7168	0.027 ± 0.006
AMC	405735/38885	0.269 ± 0.002	9070	0.248 ± 0.005	1453	0.239 ± 0.016	7221	0.018 ± 0.004
AMCp	405659/38885	0.305 ± 0.005	9070	0.244 ± 0.004	1453	0.264 ± 0.008	7221	0.101 ± 0.006
ANC	405735/38885	0.316 ± 0.001	9070	0.266 ± 0.004	1453	0.182 ± 0.007	7221	0.206 ± 0.023
ANCp	405659/38885	0.297 ± 0.003	9070	0.229 ± 0.005	1453	0.211 ± 0.006	7221	0.221 ± 0.016
apoA	323571/35052	0.413 ± 0.003	8201	0.364 ± 0.006	1284	0.328 ± 0.012	6603	0.218 ± 0.016
apoB	352981/38157	0.393 ± 0.001	8906	0.254 ± 0.003	1413	0.282 ± 0.005	7107	0.243 ± 0.006
AST	353421/38193	0.181 ± 0.003	8956	0.127 ± 0.009	1410	0.055 ± 0.001	7131	0.014 ± 0.001
Ca	112065/11643	0.015 ± 0.006	2188	0.001 ± 0.012	413	0.042 ± 0.041	1892	0.013 ± 0.010
chol	354679/38321	0.321 ± 0.006	8988	0.202 ± 0.013	1422	0.245 ± 0.021	7166	0.181 ± 0.030
Cr	354512/38298	0.240 ± 0.001	8987	0.074 ± 0.004	1421	0.011 ± 0.001	7164	0.014 ± 0.004
CRP	353928/38258	0.170 ± 0.003	8957	0.189 ± 0.005	1418	0.139 ± 0.011	7156	0.053 ± 0.011
CysC	354648/38326	0.309 ± 0.001	8989	0.146 ± 0.002	1423	0.051 ± 0.001	7166	0.046 ± 0.003
DBil	302753/32472	0.503 ± 0.000	7464	0.428 ± 0.000	1213	0.303 ± 0.003	5937	0.284 ± 0.008
E2	55693/5897	-0.015 ± 0.009	1629	0.013 ± 0.008	317	0.014 ± 0.027	1880	0.025 ± 0.018
GGT	354497/38309	0.229 ± 0.004	8987	0.198 ± 0.010	1421	0.126 ± 0.024	7164	0.040 ± 0.012
gluc	325164/35200	0.112 ± 0.008	8216	0.060 ± 0.020	1298	0.090 ± 0.016	6623	0.025 ± 0.026
HbA1c	354715/38306	0.286 ± 0.004	8885	0.169 ± 0.013	1428	0.160 ± 0.009	6219	0.024 ± 0.005
HCT	406455/38943	0.332 ± 0.001	9102	0.204 ± 0.003	1453	0.196 ± 0.009	7243	0.115 ± 0.005
HDL	325364/35234	0.451 ± 0.001	8212	0.372 ± 0.002	1298	0.305 ± 0.004	6630	0.240 ± 0.005
Hgb	406454/38943	0.341 ± 0.003	9102	0.188 ± 0.003	1453	0.187 ± 0.013	7243	0.117 ± 0.006
HLSR	399555/38356	0.328 ± 0.003	8867	0.281 ± 0.002	1422	0.271 ± 0.005	7051	0.125 ± 0.006
HLSRp	399650/38381	0.127 ± 0.004	8867	0.162 ± 0.007	1423	0.109 ± 0.006	7052	0.040 ± 0.002
IGF1	352844/38105	0.395 ± 0.001	8931	0.325 ± 0.005	1415	0.255 ± 0.004	7118	0.196 ± 0.009
IRF	399649/38381	0.316 ± 0.003	8867	0.256 ± 0.005	1423	0.194 ± 0.007	7051	0.148 ± 0.010
K	405608/38906	0.054 ± 0.016	9036	0.029 ± 0.015	1443	0.032 ± 0.014	7332	0.007 ± 0.012
LDL	354056/38261	0.330 ± 0.002	8969	0.203 ± 0.002	1420	0.249 ± 0.006	7152	0.225 ± 0.009
LpA	282560/30694	0.759 ± 0.000	7889	0.349 ± 0.001	1282	0.110 ± 0.001	5995	0.087 ± 0.001
MA	129280/11918	0.002 ± 0.008	3284	-0.003 ± 0.003	427	0.011 ± 0.034	3372	0.015 ± 0.002
MCH	406370/38943	0.435 ± 0.001	9102	0.275 ± 0.001	1453	0.224 ± 0.002	7243	0.154 ± 0.003
MCHC	406368/38942	0.168 ± 0.001	9102	0.130 ± 0.012	1453	0.122 ± 0.006	7243	0.013 ± 0.007
MCV	406373/38943	0.467 ± 0.000	9102	0.300 ± 0.003	1453	0.239 ± 0.002	7243	0.176 ± 0.006
MPV	406365/38943	0.606 ± 0.001	9102	0.520 ± 0.001	1453	0.447 ± 0.001	7243	0.342 ± 0.005
MRV	399872/38409	0.410 ± 0.001	8869	0.332 ± 0.005	1423	0.232 ± 0.005	7051	0.189 ± 0.010
MSCV	399650/38381	0.414 ± 0.000	8867	0.350 ± 0.002	1423	0.241 ± 0.003	7052	0.171 ± 0.007
Na	405587/38912	0.122 ± 0.006	9035	0.084 ± 0.004	1444	-0.003 ± 0.018	7328	0.035 ± 0.004
NRBC	405536/38864	0.001 ± 0.003	9070	-0.002 ± 0.004	1453	0.022 ± 0.025	7221	0.025 ± 0.003
NRBCp	405452/38863	0.003 ± 0.004	9070	-0.005 ± 0.004	1453	0.007 ± 0.010	7221	0.022 ± 0.008
PCT	406263/38923	0.437 ± 0.003	9101	0.365 ± 0.004	1453	0.292 ± 0.011	7243	0.152 ± 0.009
PDW	406181/38923	0.476 ± 0.001	9101	0.417 ± 0.001	1453	0.320 ± 0.003	7243	0.223 ± 0.007
phos	324910/35172	0.255 ± 0.002	8207	0.226 ± 0.009	1296	0.199 ± 0.020	6621	0.112 ± 0.013
PLT	406451/38943	0.486 ± 0.001	9102	0.416 ± 0.002	1453	0.343 ± 0.000	7243	0.219 ± 0.007
RBC	406455/38943	0.417 ± 0.002	9102	0.277 ± 0.004	1453	0.227 ± 0.010	7243	0.161 ± 0.006
RDW	406373/38943	0.304 ± 0.002	9102	0.211 ± 0.001	1453	0.198 ± 0.004	7243	0.097 ± 0.002
RET	399797/38388	0.162 ± 0.002	8869	0.167 ± 0.018	1423	0.199 ± 0.028	7051	0.050 ± 0.001
RETp	399872/38409	0.139 ± 0.006	8869	0.158 ± 0.023	1423	0.189 ± 0.042	7052	0.042 ± 0.002
RF	31224/3336	0.033 ± 0.013	737	0.046 ± 0.018	112	0.038 ± 0.038	534	-0.012 ± 0.022
SHBG	322452/34921	0.392 ± 0.002	8162	0.289 ± 0.005	1288	0.255 ± 0.001	6567	0.173 ± 0.019
T	321936/34635	0.164 ± 0.003	8217	0.140 ± 0.002	1329	0.069 ± 0.005	6495	0.017 ± 0.009
TBil	353257/38169	0.591 ± 0.000	8951	0.554 ± 0.001	1409	0.473 ± 0.003	7126	0.431 ± 0.018
TG	354401/38292	0.356 ± 0.004	8977	0.310 ± 0.013	1421	0.242 ± 0.013	7164	0.127 ± 0.019
TP	325163/35189	0.326 ± 0.001	8218	0.278 ± 0.001	1297	0.244 ± 0.006	6631	0.118 ± 0.007
U	354448/38292	0.228 ± 0.004	8987	0.165 ± 0.003	1420	0.101 ± 0.006	7164	0.059 ± 0.012
UA	354244/38288	0.393 ± 0.003	8977	0.328 ± 0.001	1420	0.287 ± 0.005	7160	0.204 ± 0.017
UCR	406454/38985	0.097 ± 0.005	9051	0.049 ± 0.002	1445	0.031 ± 0.005	7335	0.027 ± 0.005
vitD	112065/11643	0.013 ± 0.000	2188	0.016 ± 0.004	413	0.046 ± 0.012	1892	-0.024 ± 0.003
WBC	406450/38943	0.256 ± 0.001	9102	0.256 ± 0.001	1453	0.201 ± 0.002	7243	0.077 ± 0.002

Scatter plots for one out of the five PGS predictors for each phenotype is displayed in **Figure S6** to **Figure S16**. A linear fit is also shown together with the identity line in each subplot.

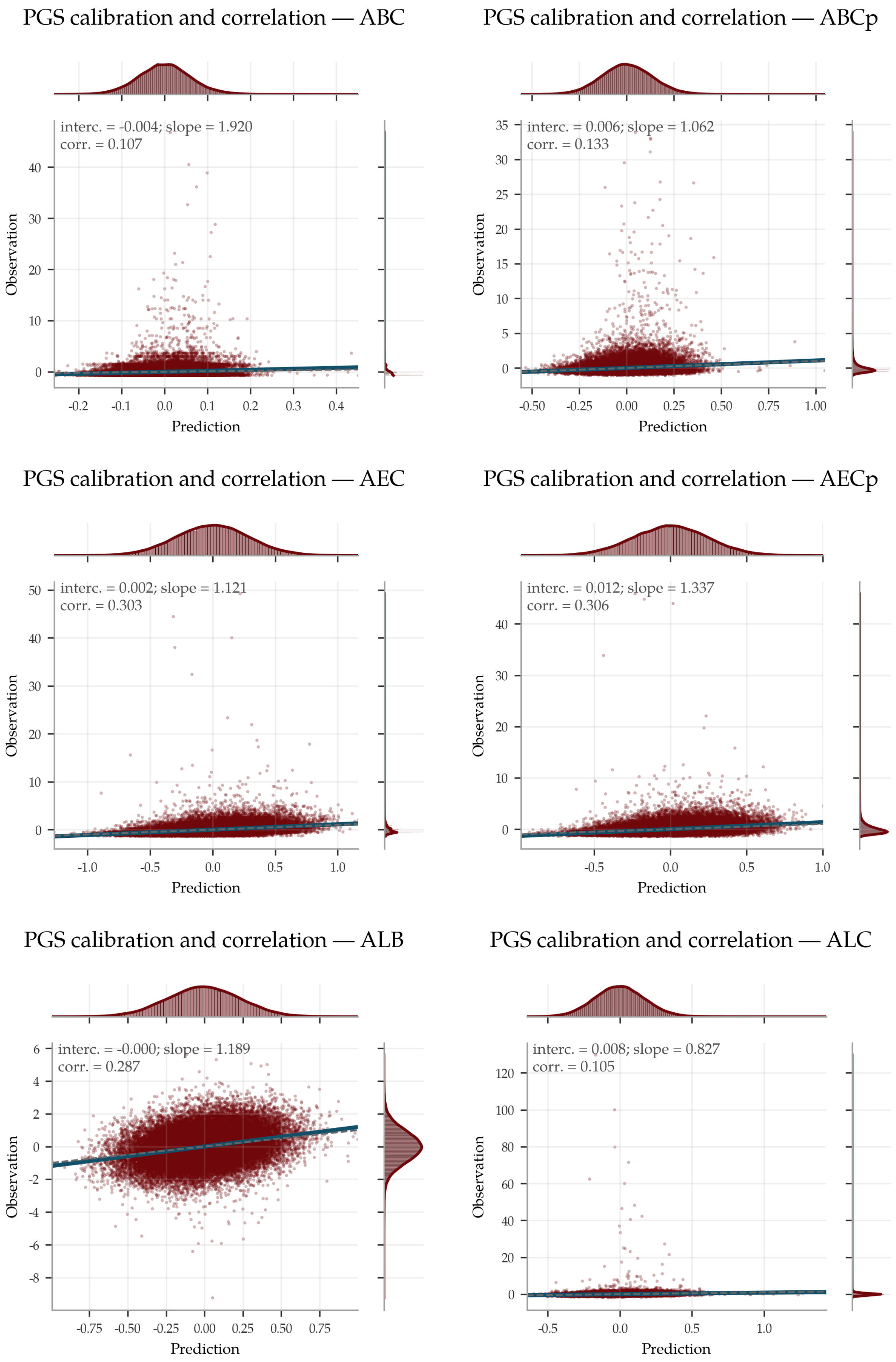

Figure S6: PGS scatter plots with linear fit and identity line (1 of 11). The predictors were evaluated on their corresponding evaluation sets of $\sim$ 40k European siblings.

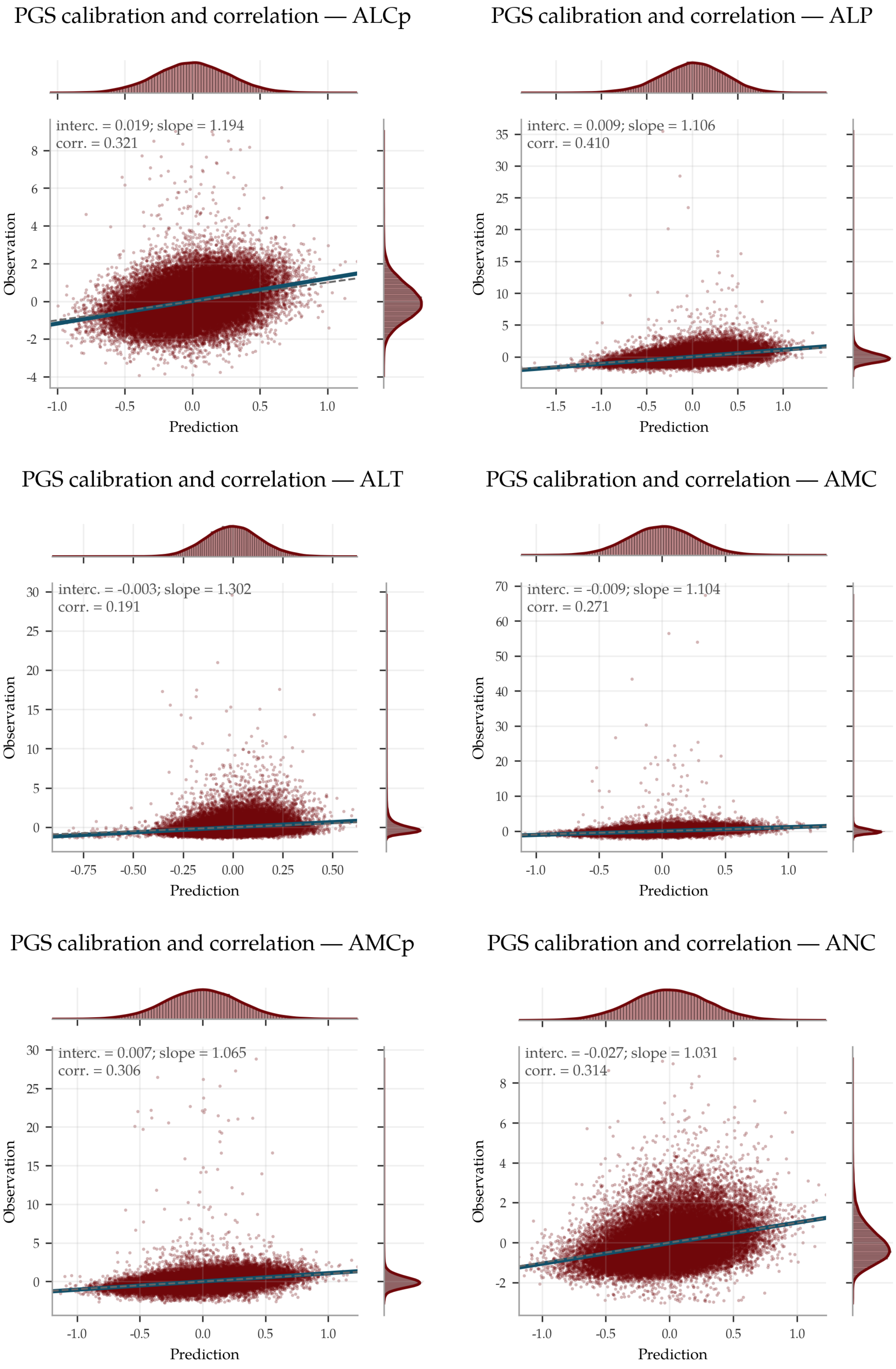

Figure S7: PGS scatter plots with linear fit and identity line (2 of 11). The predictors were evaluated on their corresponding evaluation sets of ~ 40k European siblings.

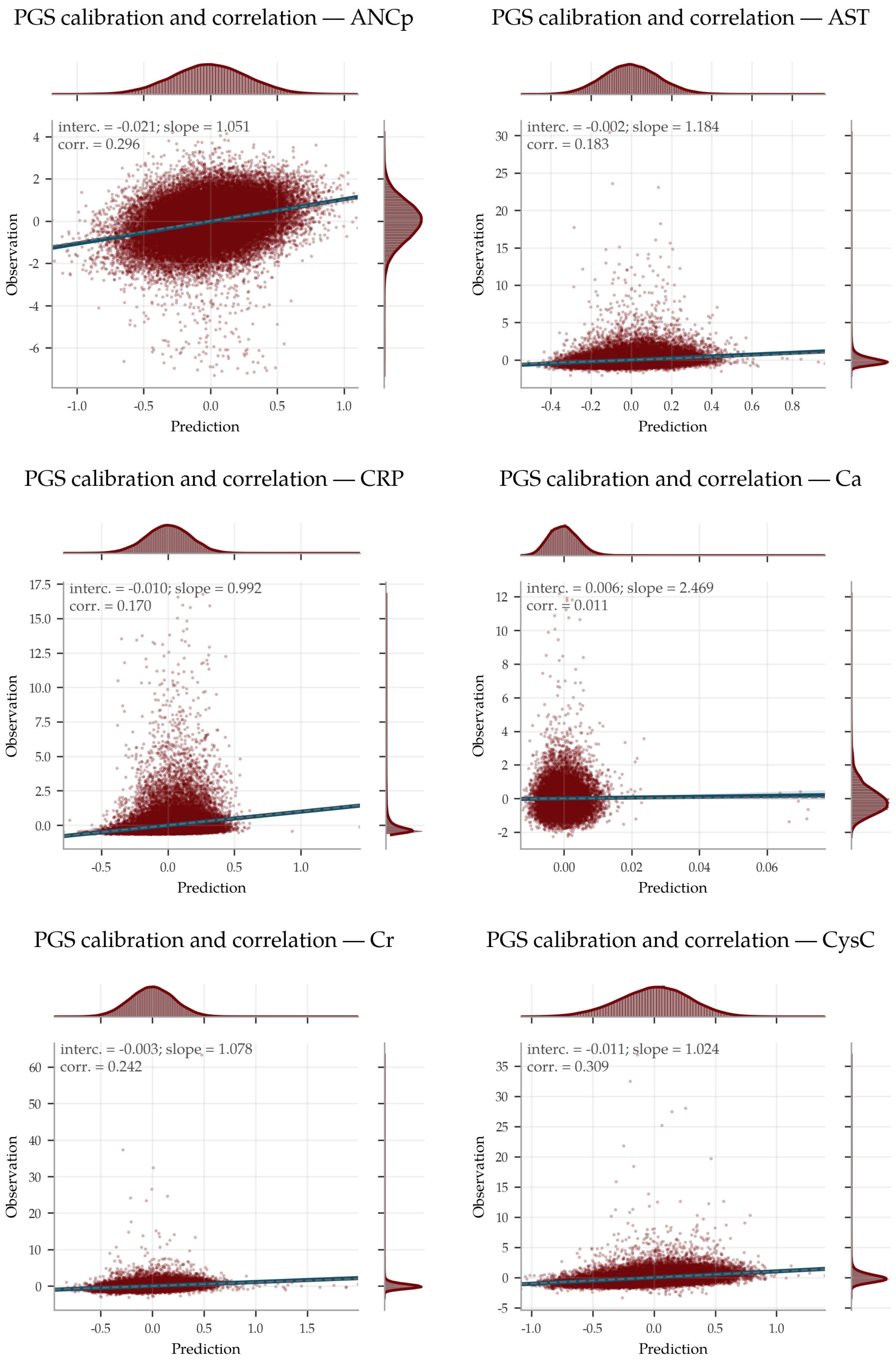

Figure S8: PGS scatter plots with linear fit and identity line (3 of 11). The predictors were evaluated on their corresponding evaluation sets of ~ 40k European siblings.

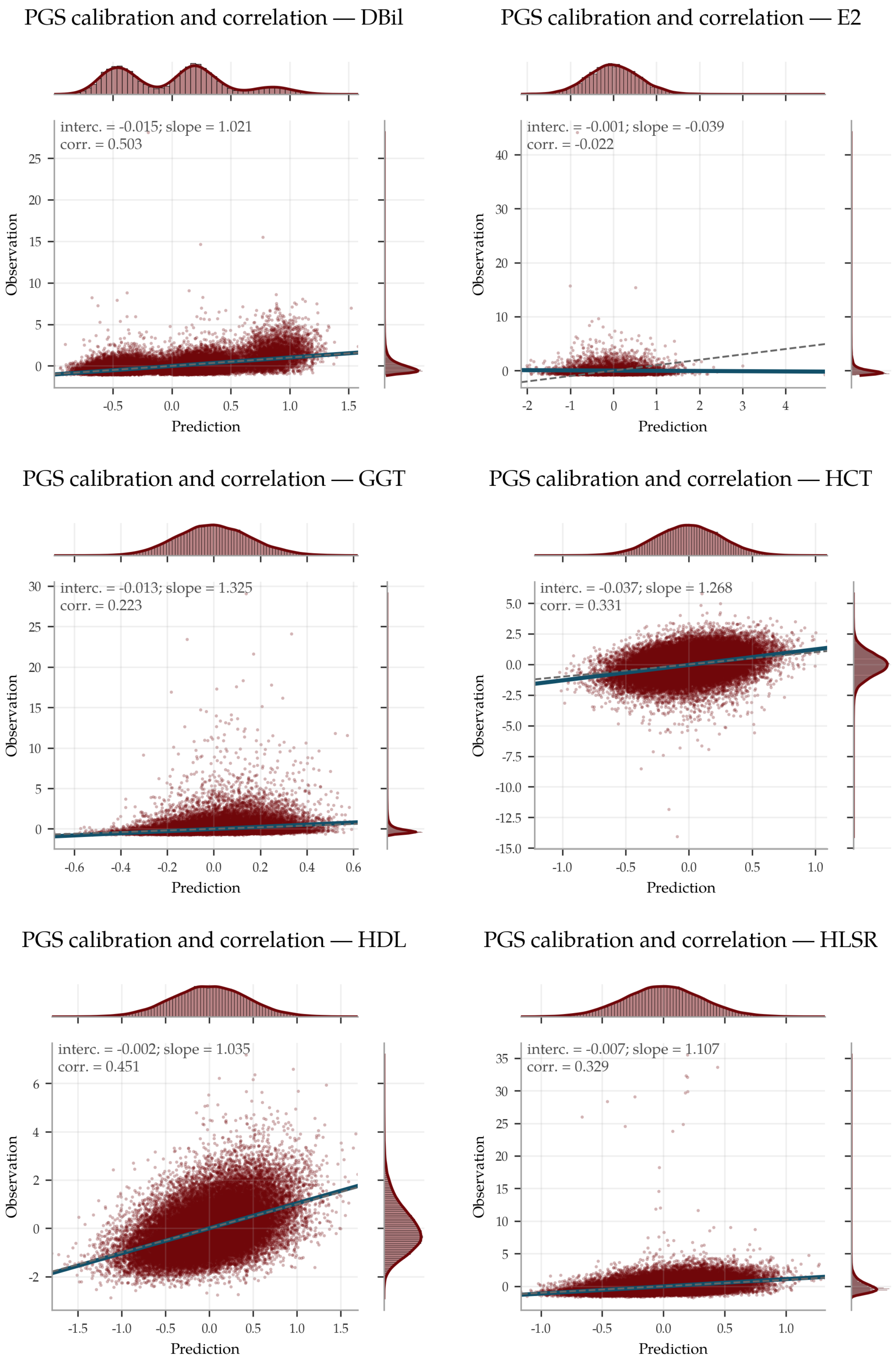

Figure S9: PGS scatter plots with linear fit and identity line (4 of 11). The predictors were evaluated on their corresponding evaluation sets of $\sim$ 40k European siblings.

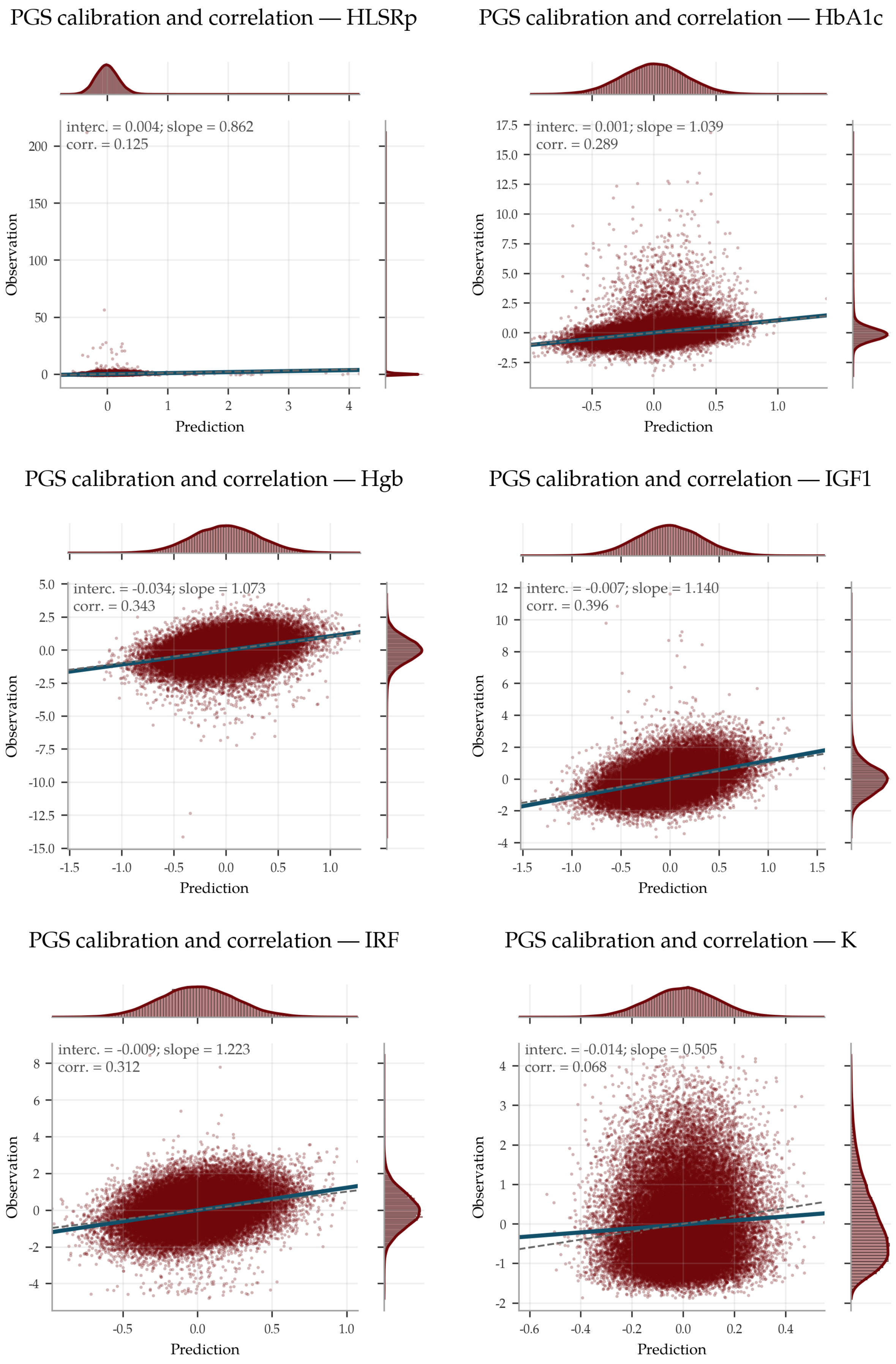

Figure S10: PGS scatter plots with linear fit and identity line (5 of 11). The predictors were evaluated on their corresponding evaluation sets of $\sim$ 40k European siblings.

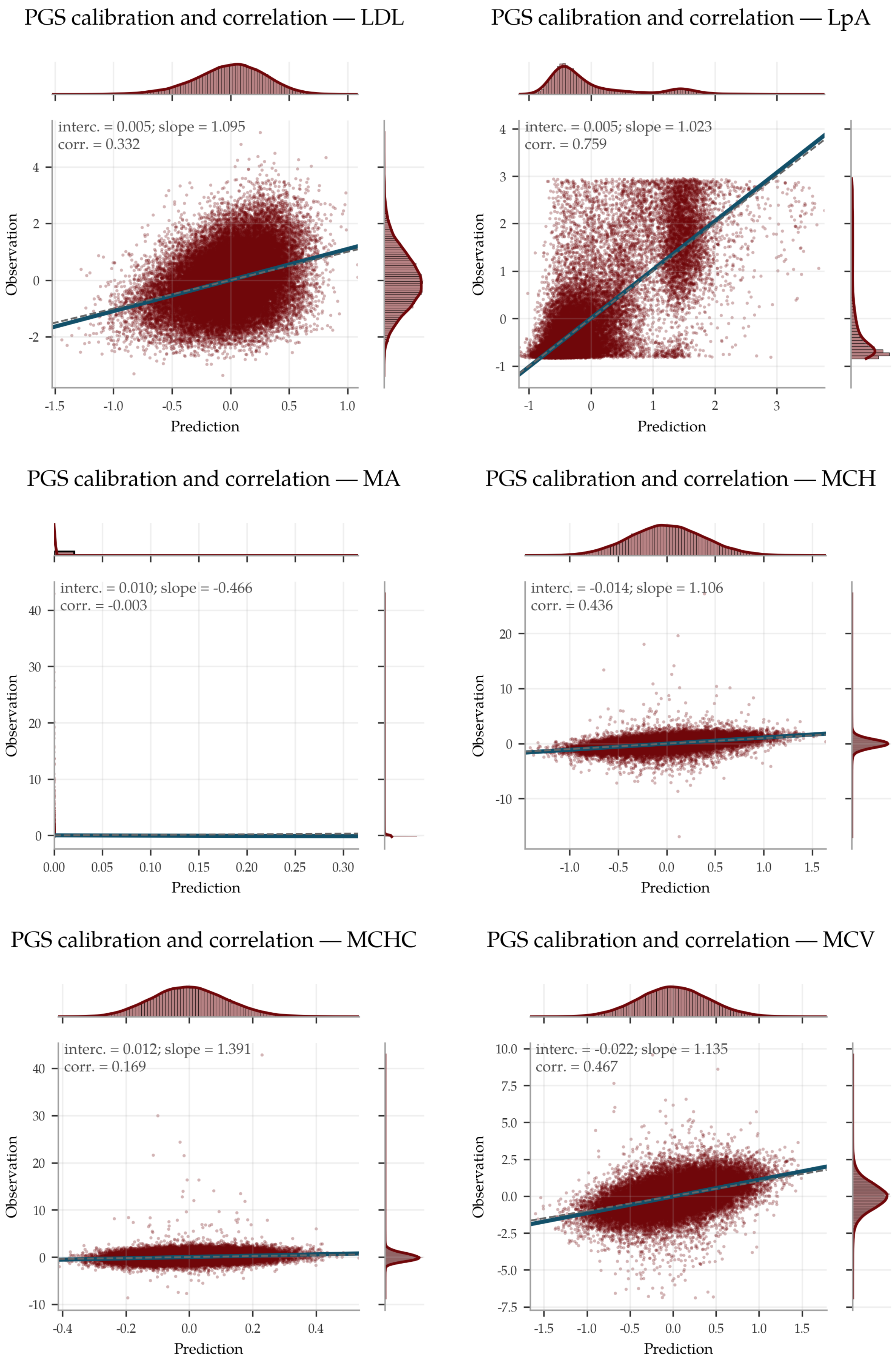

Figure S11: PGS scatter plots with linear fit and identity line (6 of 11). The predictors were evaluated on their corresponding evaluation sets of $\sim$ 40k European siblings.

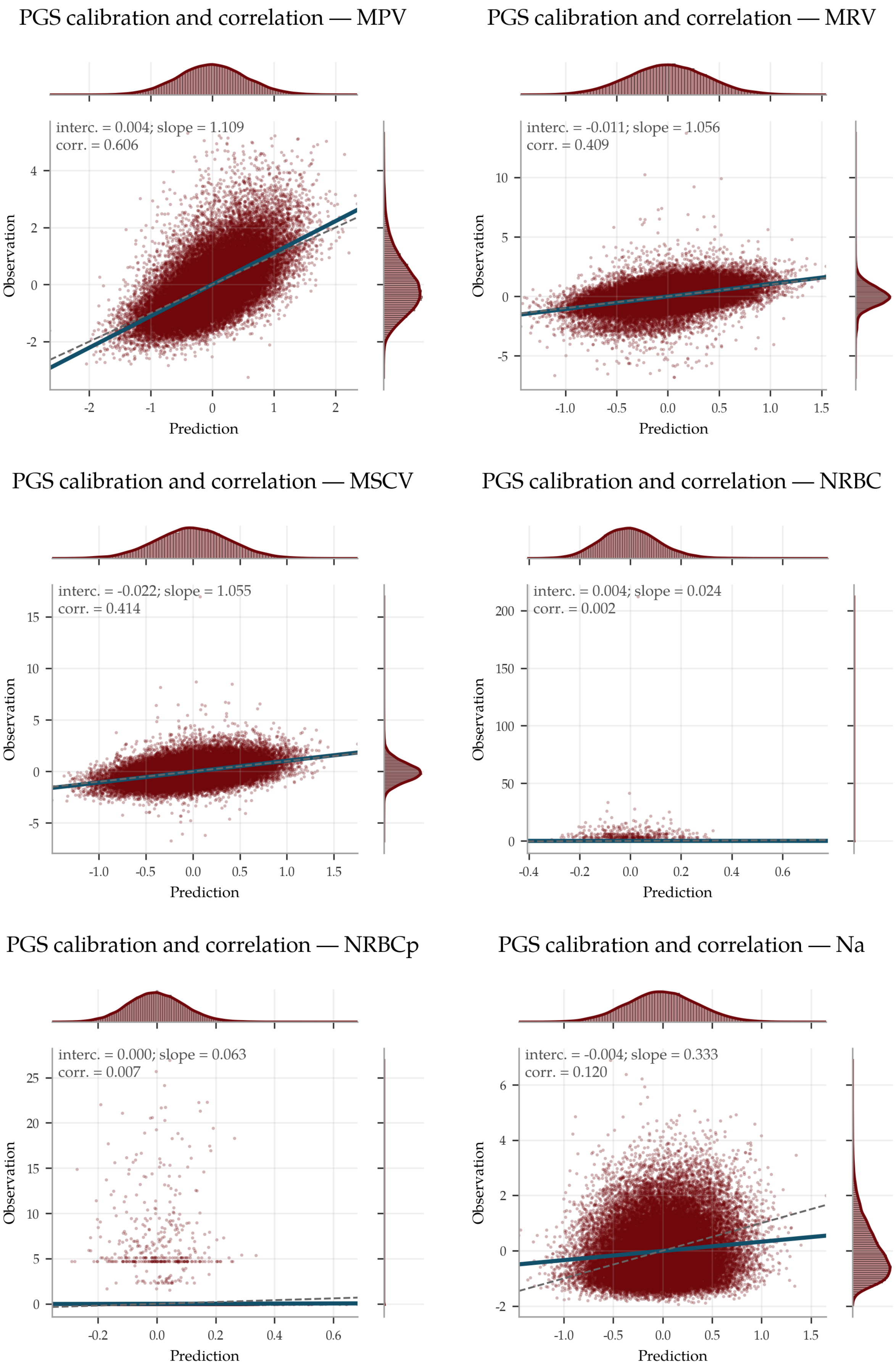

Figure S12: PGS scatter plots with linear fit and identity line (7 of 11). The predictors were evaluated on their corresponding evaluation sets of ~ 40k European siblings.

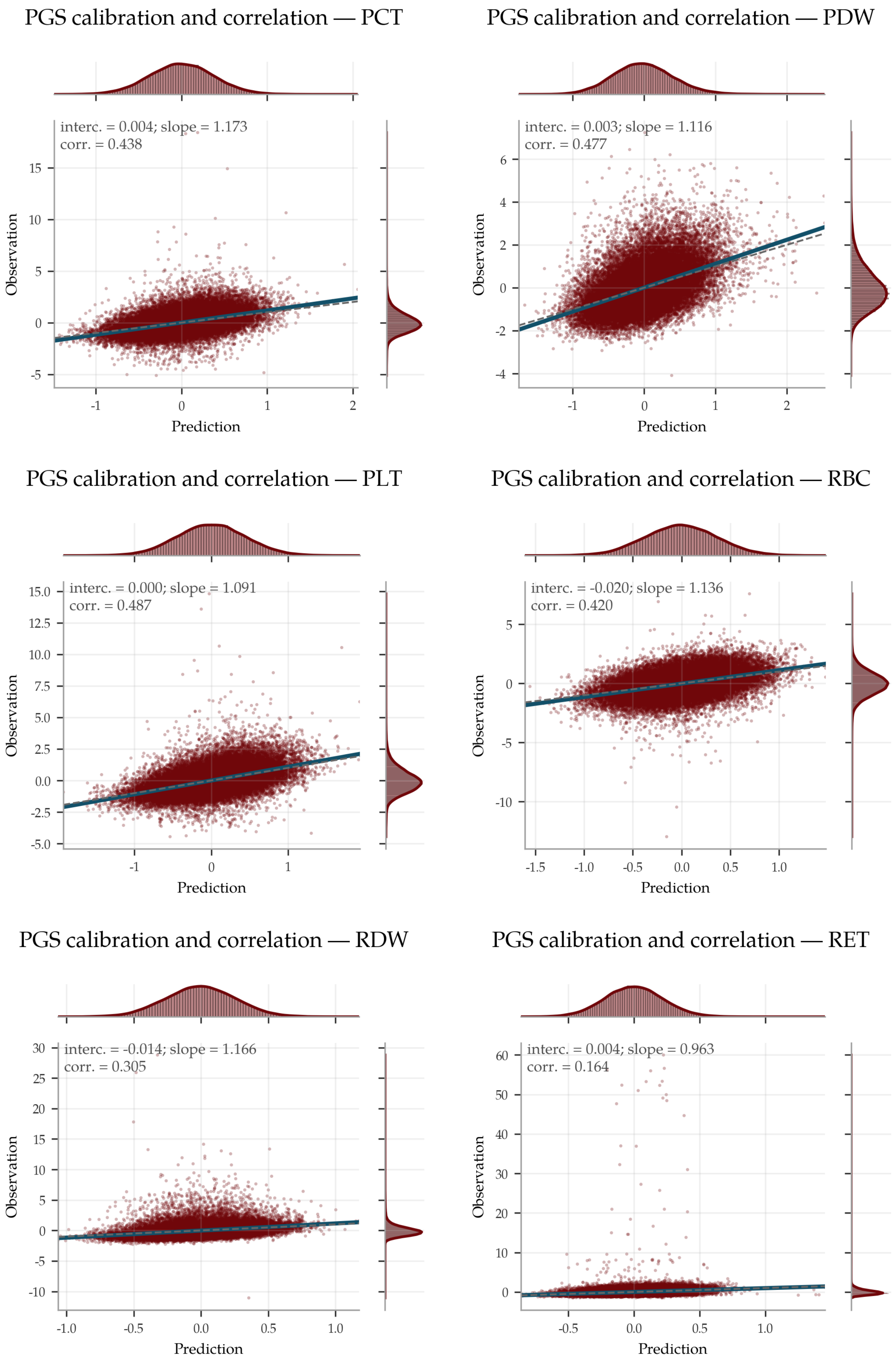

Figure S13: PGS scatter plots with linear fit and identity line (8 of 11). The predictors were evaluated on their corresponding evaluation sets of $\sim$ 40k European siblings.

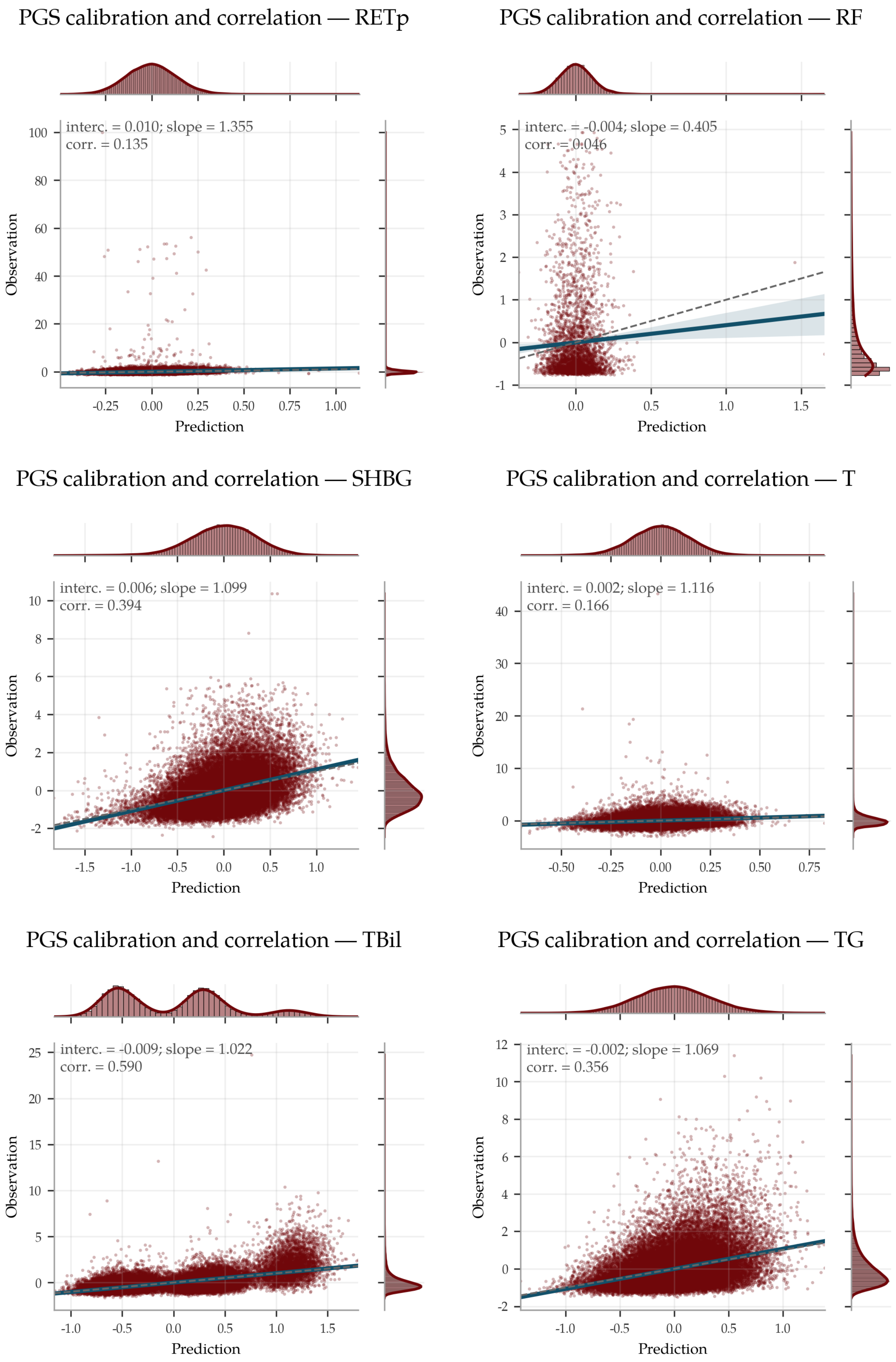

Figure S14: PGS scatter plots with linear fit and identity line (9 of 11). The predictors were evaluated on their corresponding evaluation sets of $\sim$ 40k European siblings.

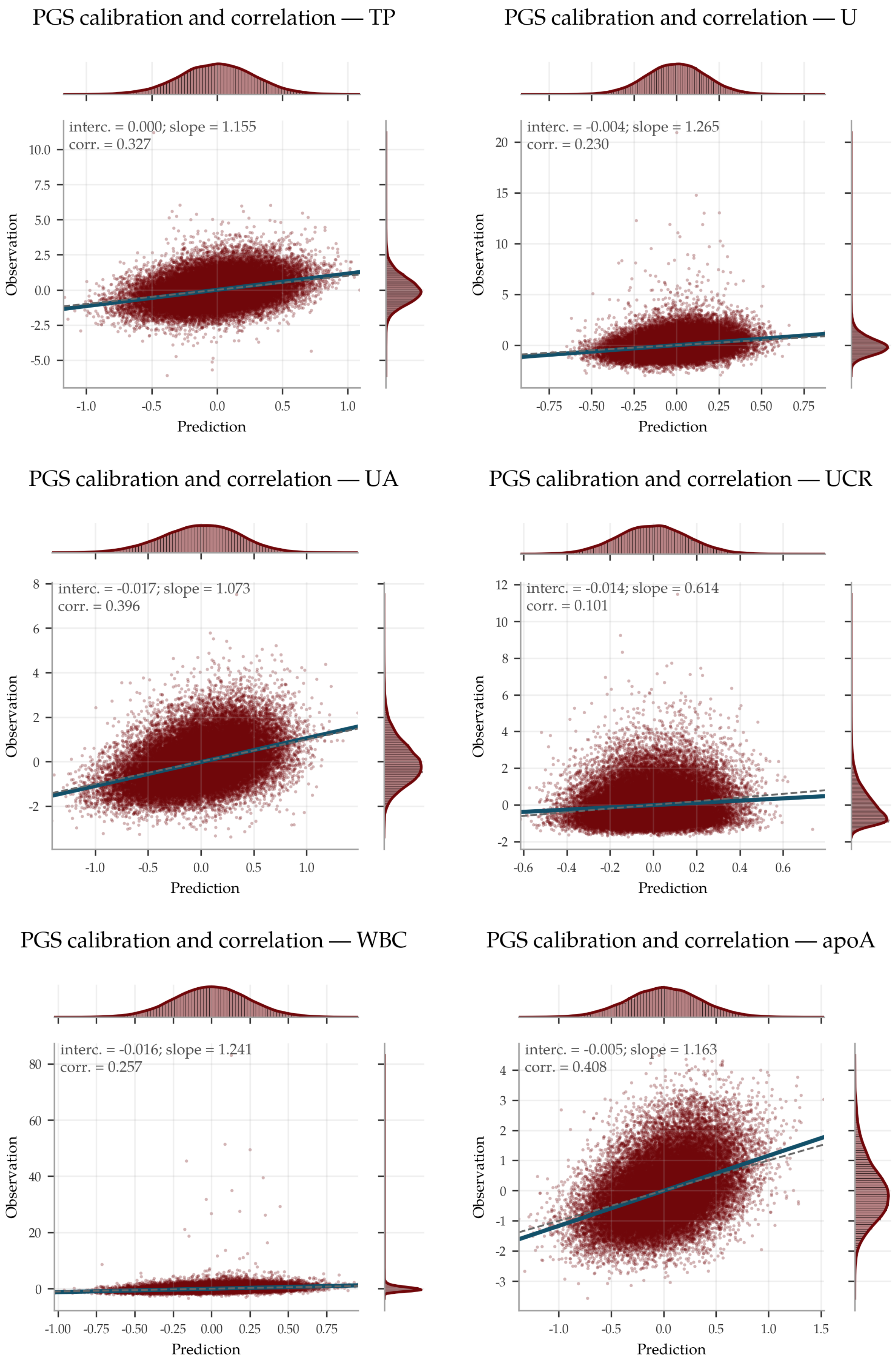

Figure S15: PGS scatter plots with linear fit and identity line (10 of 11). The predictors were evaluated on their corresponding evaluation sets of ~ 40k European siblings.

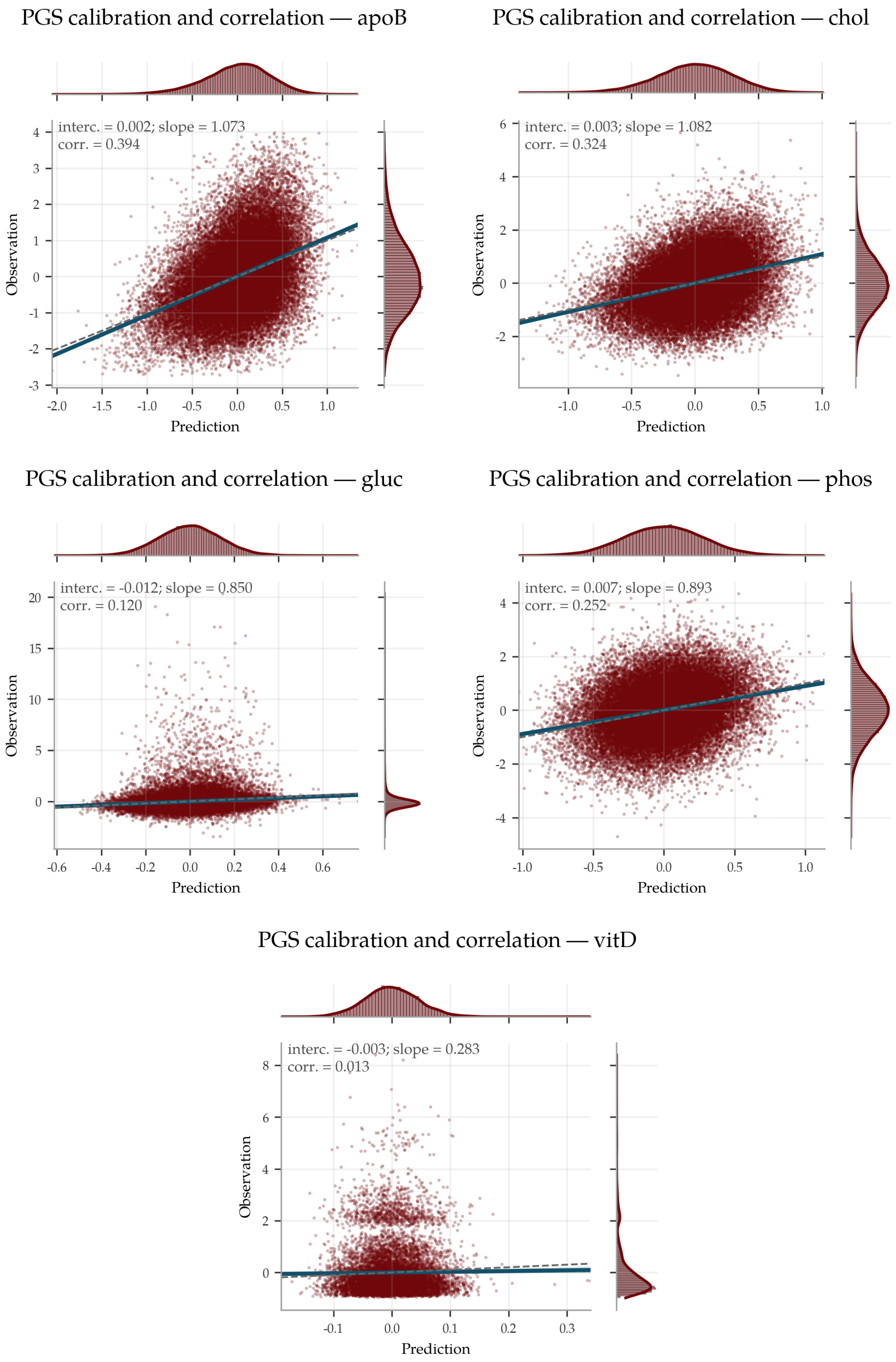

Figure S16: PGS scatter plots with linear fit and identity line (11 of 11). The predictors were evaluated on their corresponding evaluation sets of ~ 40k European siblings.

S3.3 Sibling evaluation

The main document presented the sibling evaluation only for the PGS predictors with the highest correlations. The full figure with all biomarkers is shown in **Figure S17**, while the sample sizes used are listed in **Table S3** (vitamin D was left out of this analysis due to too few samples). Again, sib 0.5, sib 1.0 and sib 1.5, are the results when restricting to siblings with phenotype differences larger than 0.5, 1 and 1.5 standard deviations, respectively.

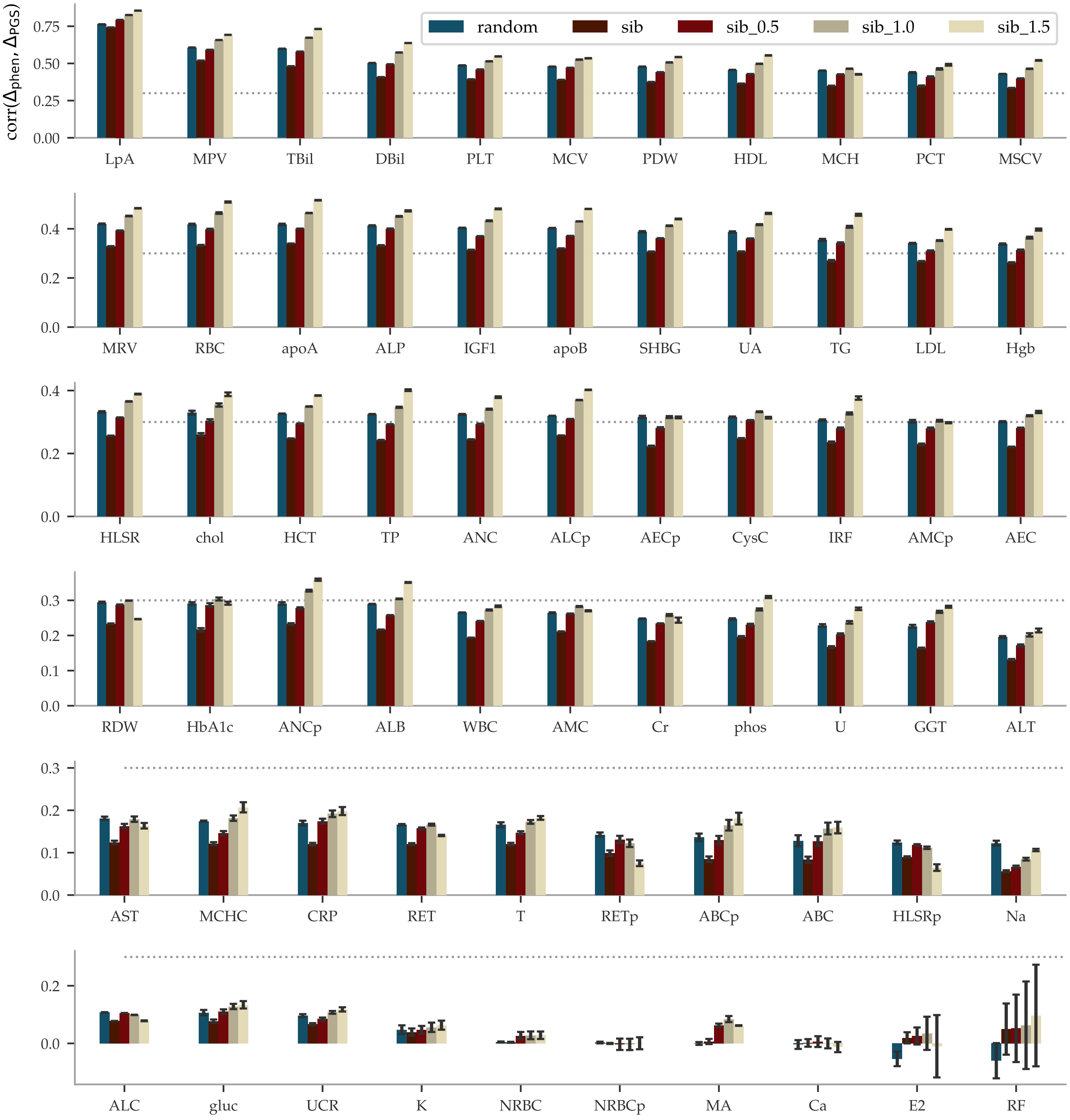

Figure S17: Sibling comparisons of correlation between difference in phenotype and difference in PGS, i.e., corr($\Delta_{\text{phen}}, \Delta_{\text{PGS}}$). UKBs ∼ 40k European siblings were paired either randomly or as genetic siblings. The error bars indicate ± the standard deviations for 5 predictors trained on slightly different training sets. The additional three bars labeled sib 0.5, sib 1.0 and sib 1.5, are the results when restricting to siblings with phenotype differences larger than 0.5, 1 and 1.5 standard deviations, respectively. Vitamin D was left out because of too few samples.

Abbr.	random	sib	sib_0.5	sib_1.0	sib_1.5	Abbr.	random	sib	sib_0.5	sib_1.0	sib_1.5
ABC	19645	20469	8442	4631	2992	IRF	19146	19974	13388	8011	4163
ABCp	19645	20469	7932	3398	1666	K	19622	20465	14023	8984	5416
AEC	19645	20469	10512	4714	2189	LDL	19016	19829	13478	8319	4589
AECp	19645	20469	10598	5097	2550	LpA	12265	13386	4967	2921	2116
ALB	16129	16861	11401	6889	3668	MA	1863	2173	187	99	60
ALC	19645	20469	6964	1623	403	MCH	19701	20527	10702	4508	1778
ALCp	19645	20469	13359	7490	3637	MCHC	19700	20526	13214	7407	3648
ALP	19089	19897	11675	5830	2737	MCV	19701	20527	11484	5250	2056
ALT	19081	19890	10125	4904	2617	MPV	19701	20527	12933	6901	3340
AMC	19645	20469	10748	4660	1843	MRV	19180	20006	12542	6897	3404
AMCp	19645	20469	11720	5712	2483	MSCV	19146	19974	12735	7044	3370
ANC	19645	20469	13186	7498	3938	Na	19635	20474	13260	7799	4321
ANCp	19645	20469	13449	7608	3809	NRBC	19620	20445	427	416	383
apoA	15958	16679	11047	6502	3440	NRBCp	19619	20444	427	426	403
apoB	18920	19723	13301	8078	4421	PCT	19677	20504	12949	7130	3518
AST	18959	19767	9259	3804	1715	PDW	19677	20504	13257	7459	3813
Ca	1699	2473	1493	763	396	phos	16060	16792	11540	7164	3984
chol	19087	19893	13501	8184	4490	PLT	19701	20527	12907	7035	3426
Cr	19056	19865	9613	3531	1266	RBC	19701	20527	12783	6899	3174
CRP	19018	19825	6367	3174	1964	RDW	19701	20527	10446	4397	1922
CysC	19091	19897	10476	4506	1852	RET	19159	19983	8492	2666	715
DBil	13672	14459	7912	4107	2208	RETp	19180	20006	7607	2040	481
E2	460	774	383	205	122	RF	149	223	120	73	48
GGT	19069	19877	6538	3148	1832	SHBG	15840	16552	10487	5881	3155
gluc	16092	16819	6466	2435	1256	T	15599	16326	10503	6015	3180
HbA1c	19058	19886	8339	2901	1334	TBil	18928	19738	10334	4891	2550
HCT	19701	20527	13477	7831	3944	TG	19050	19860	11203	6170	3431
HDL	16122	16853	11004	6384	3318	TP	16089	16810	10919	6198	3203
Hgb	19701	20527	13103	7259	3609	U	19048	19853	12931	7440	3797
HLSR	19121	19947	11704	6032	2954	UA	19053	19858	13164	7703	4034
HLSRp	19146	19974	7876	2452	726	UCR	19711	20555	12872	7594	4194
IGF1	18871	19672	12913	7297	3673	WBC	19701	20527	11937	5693	2424

Table S3: Number of pairs in each evaluation set used for Figure S17.

S3.4 Genetic Architectures

The β coefficient sizes of one predictor for every biomarker are plotted in **Figure S18** to **Figure S26**. The same figures also feature the cumulative variance accounted for from single SNPs, as described in eq. (4). Number of non-zero coefficients ($\pm$ standard deviation over 5 predictors) and PGS-phenotype correlations are listed within each plot title.

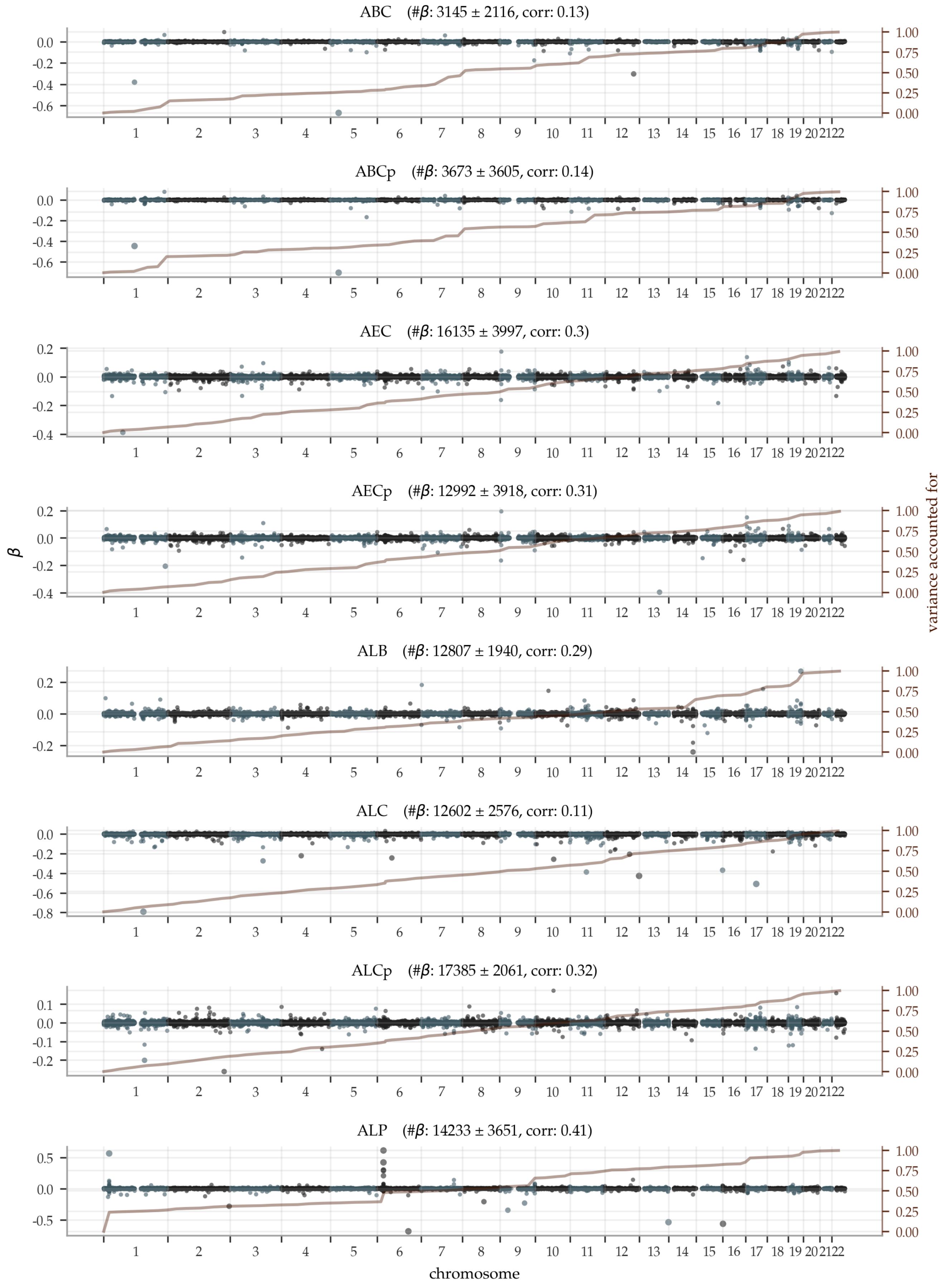

Figure S18: Manhattan plots (1 of 9) of PGS predictor β with superimposed variance accounted for.

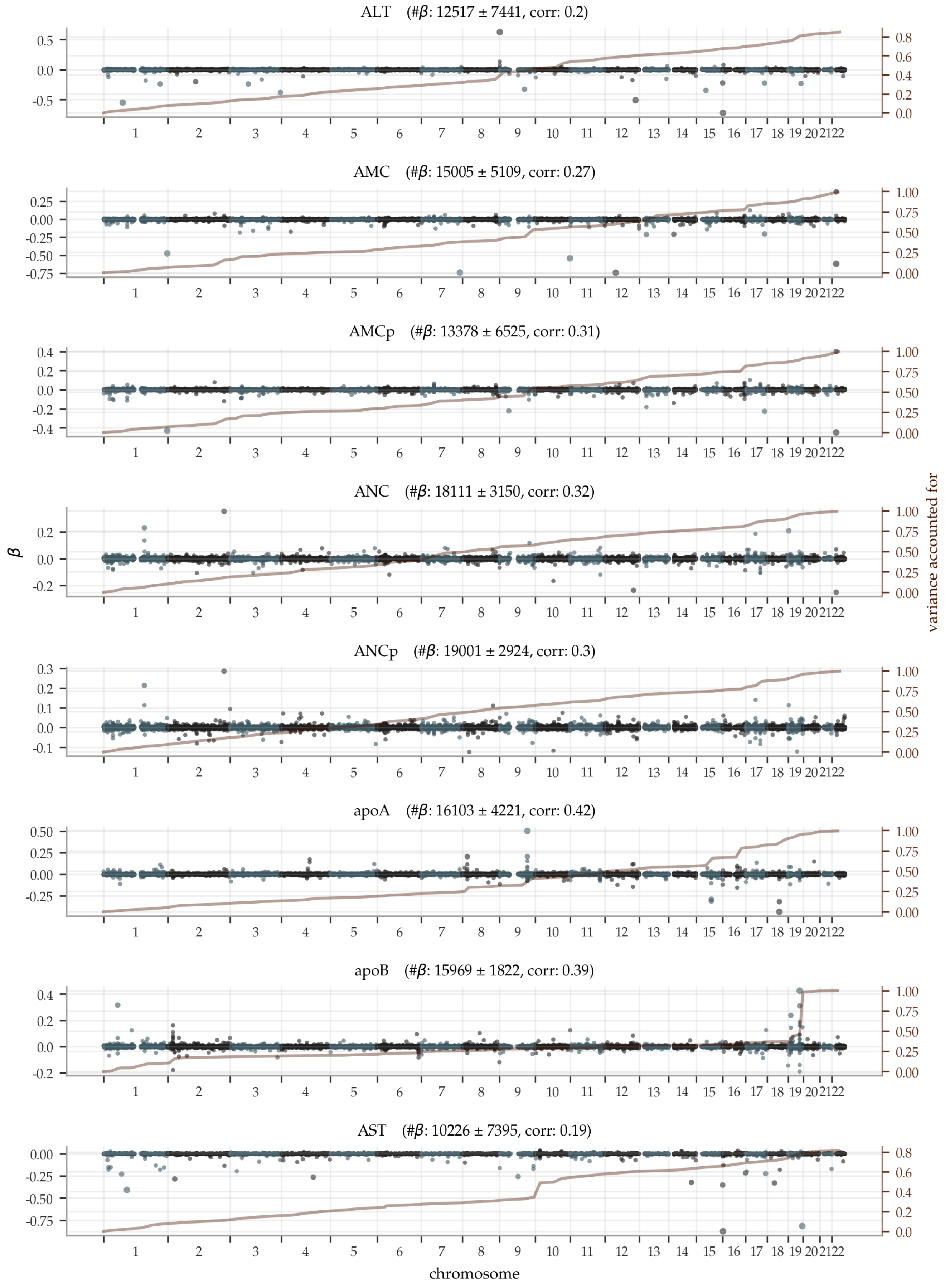

Figure S19: **Manhattan plots (2 of 9) of PGS predictor β with superimposed variance accounted for.**

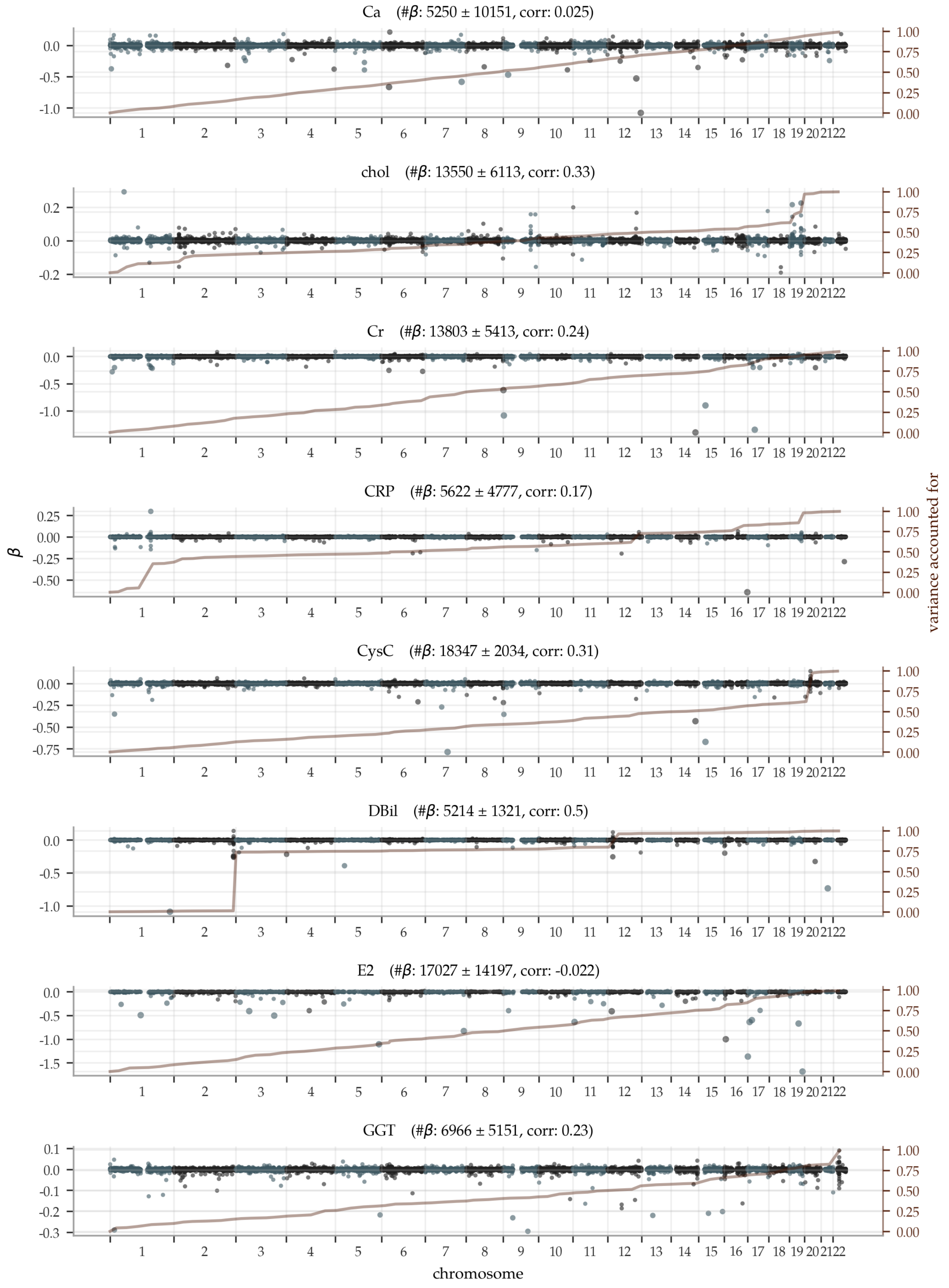

Figure S20: **Manhattan plots (3 of 9) of PGS predictor β with superimposed variance accounted for.**

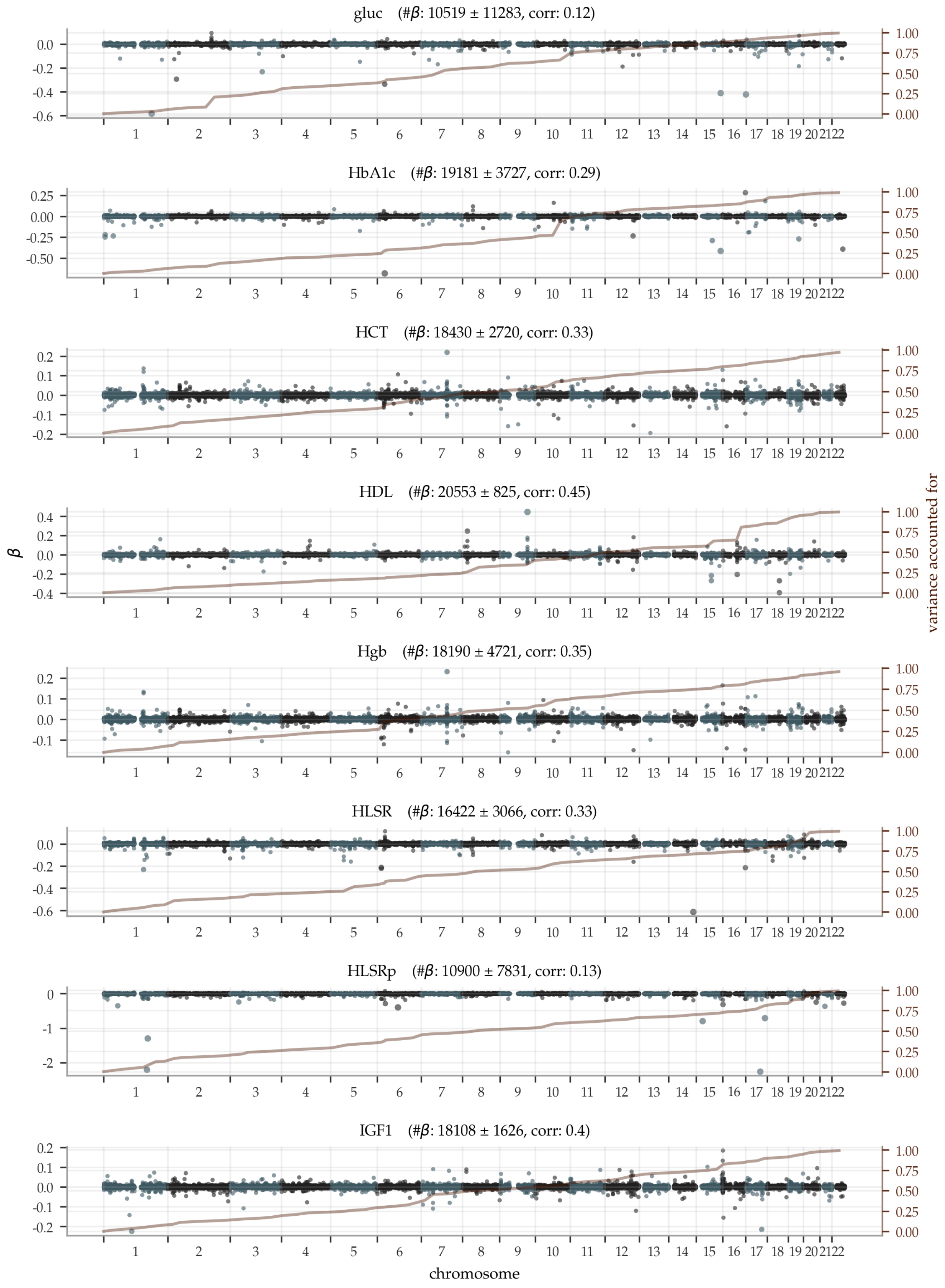

Figure S21: Manhattan plots (4 of 9) of PGS predictor β with superimposed variance accounted for.

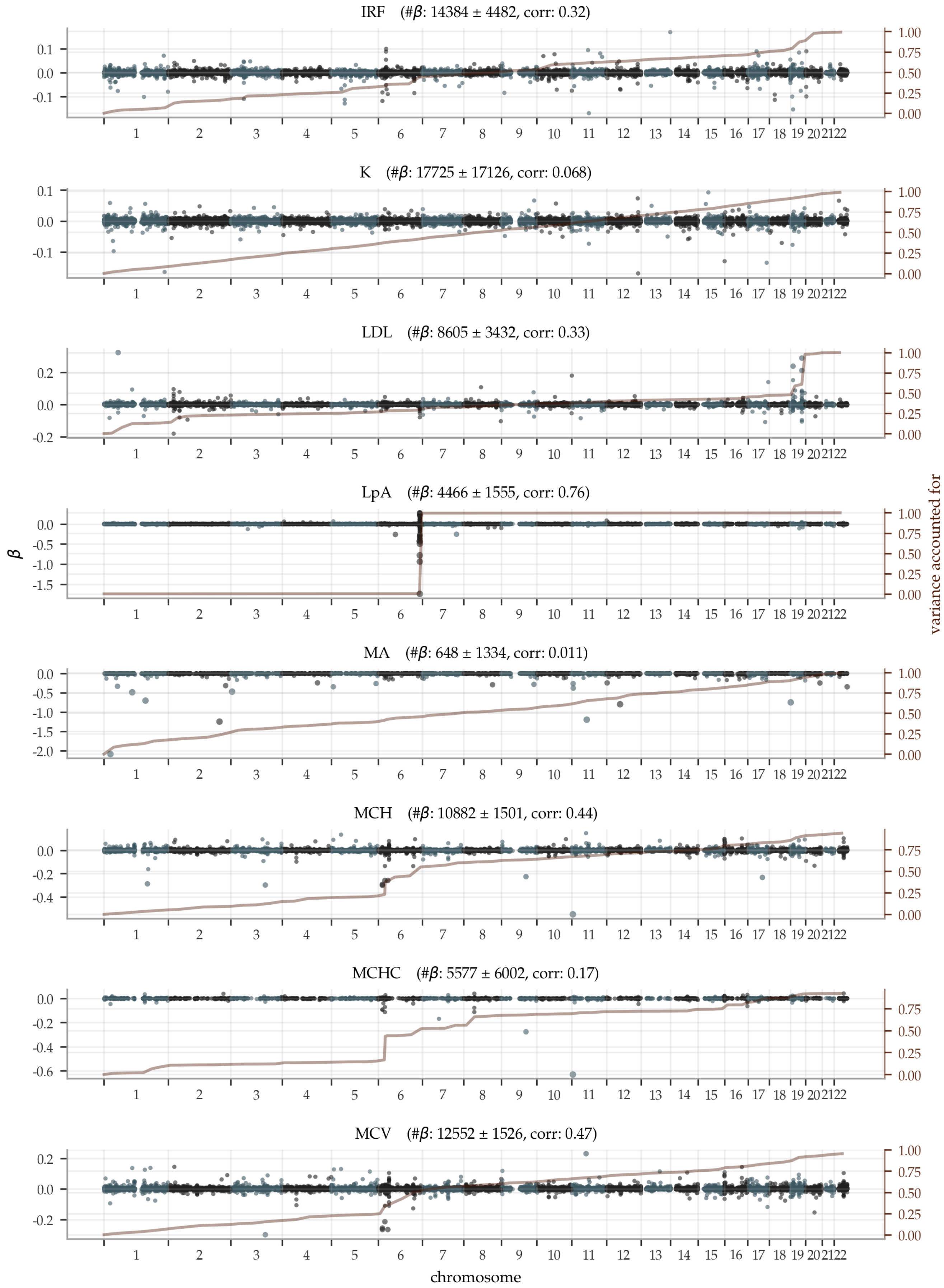

Figure S22: Manhattan plots (5 of 9) of PGS predictor β with superimposed variance accounted for.

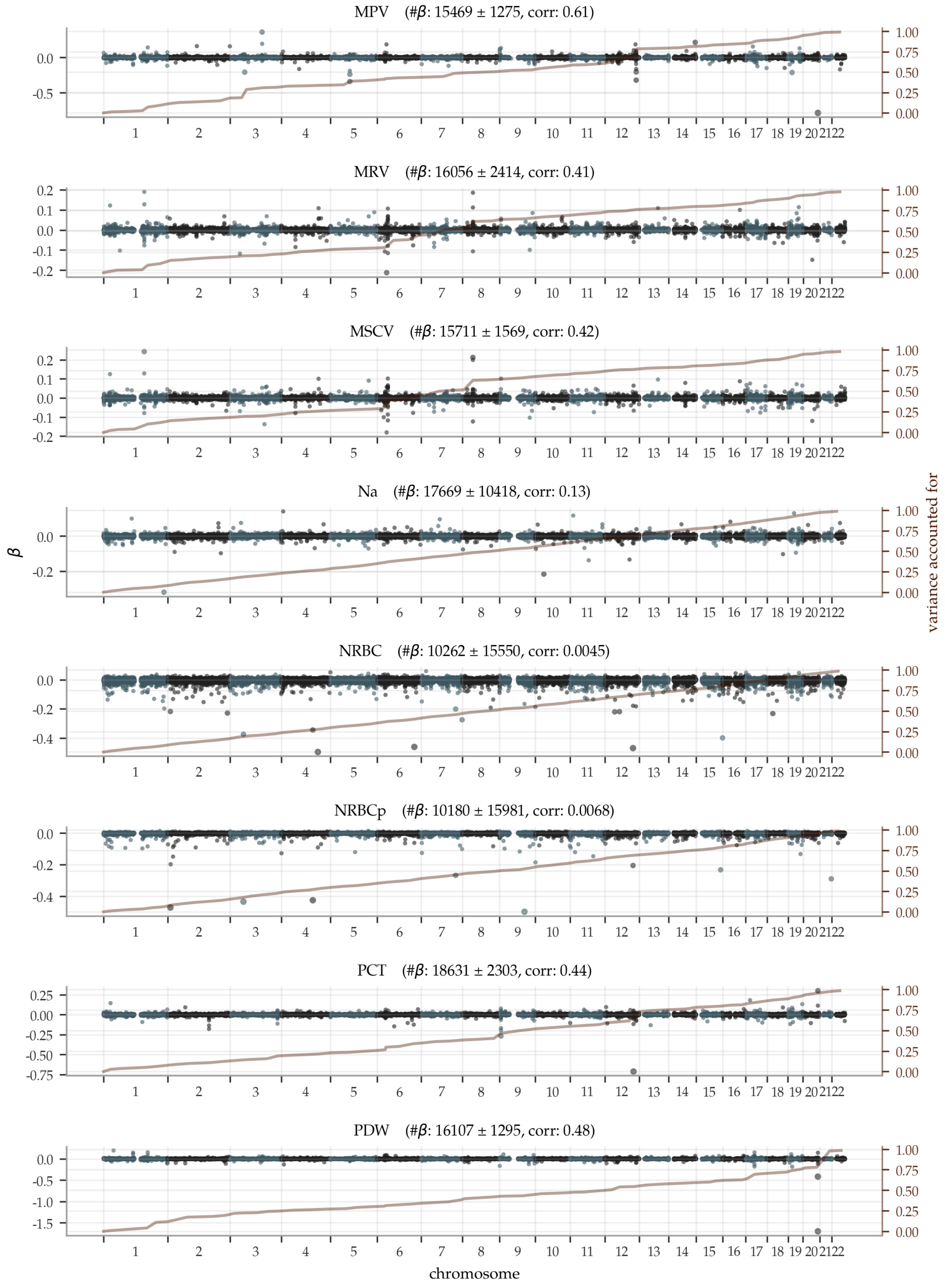

Figure S23: **Manhattan plots (6 of 9) of PGS predictor β with superimposed variance accounted for.**

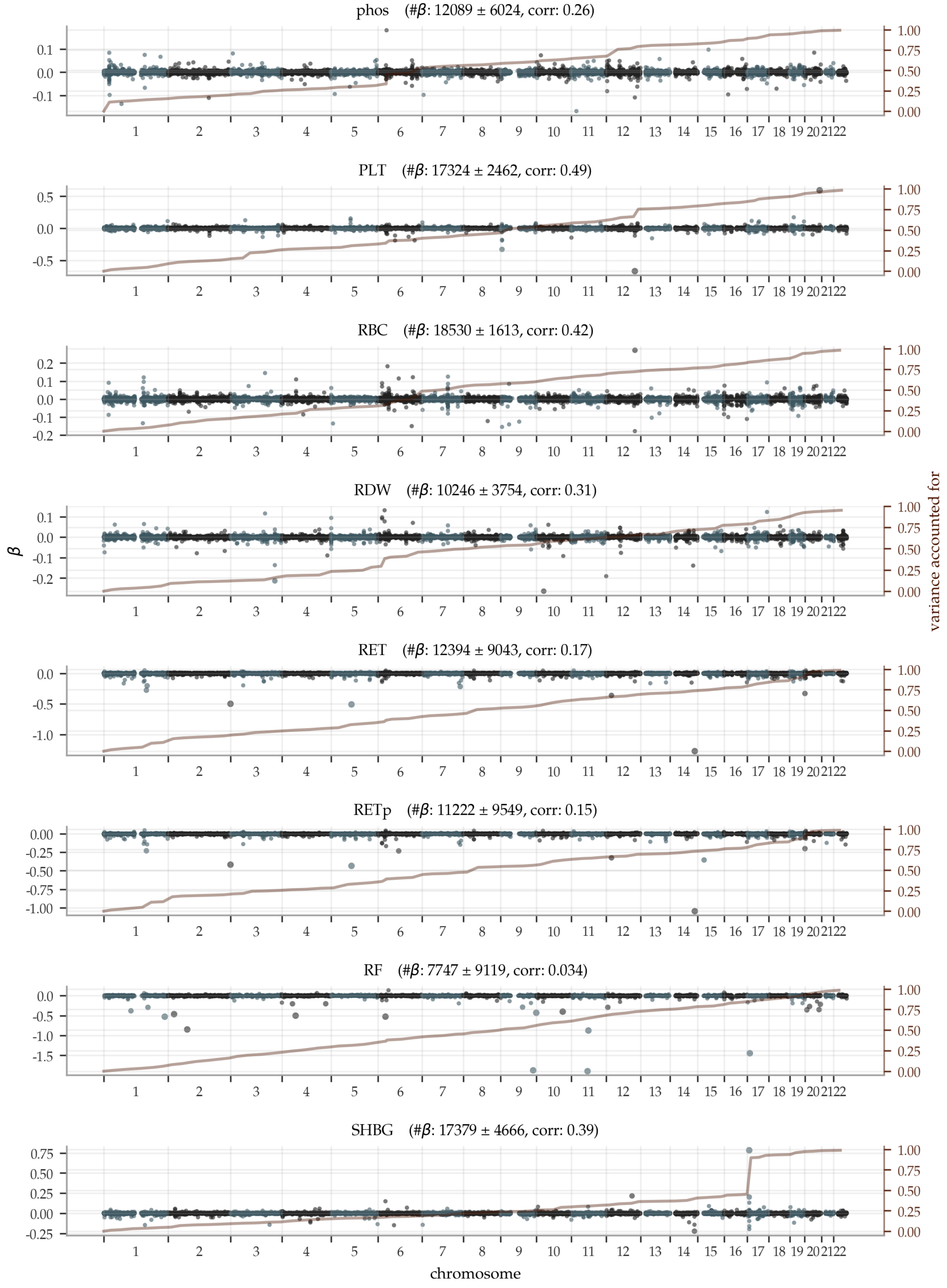

Figure S24: Manhattan plots (7 of 9) of PGS predictor β with superimposed variance accounted for.

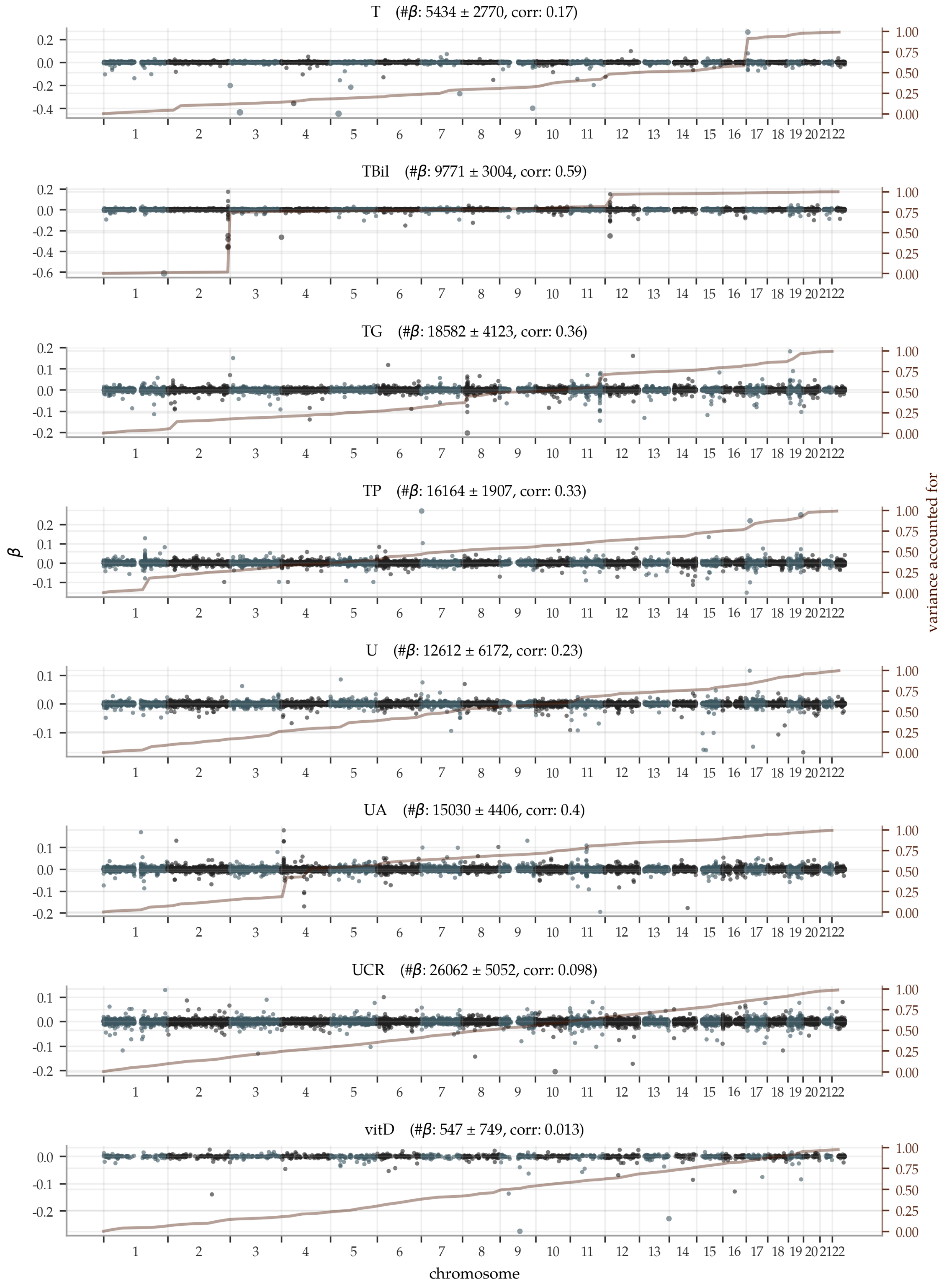

Figure S25: **Manhattan plots (8 of 9) of PGS predictor β with superimposed variance accounted for.**

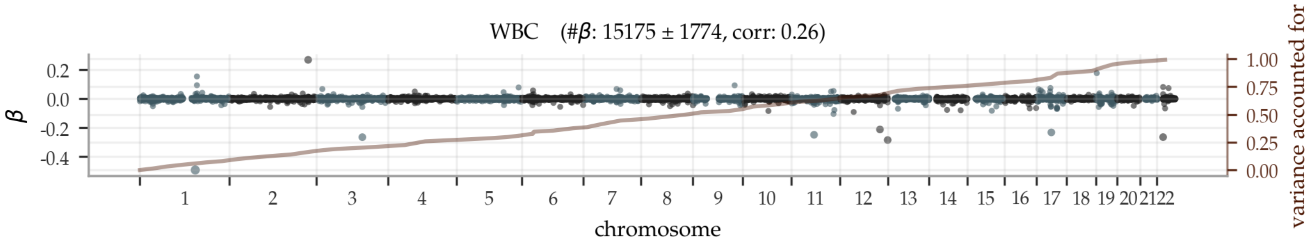

Figure S26: Manhattan plots (9 of 9) of PGS predictor β with superimposed variance accounted for.

S4 Disease Prediction

S4.1 Disease condition definitions

The disease conditions were defined in terms of matching codes in a number of fields in the UKB. Each condition is listed below with all its codes for all UKB fields involved. An individual was classified as a case if any of the disease codes was present.

CAD

Non-cancer illness code, self-reported, UKB field 20002 1075

Diagnoses - ICD9, UKB field 41271 410, 4109, 412, 4129

Diagnoses - ICD10, UKB field 41270 I210, I211, I212, I213, I214, I219, I21X, I22, I220, I221, I228, I229, I23, I230, I231, I232, I233, I234, I235, I236, I238, I241, I252

Operative procedures - OPCS4, UKB field 41272 K401, K402, K403, K404, K411, K412, K413, K414, K451, K452, K453, K454, K455, K491, K492, K498, K499, K502, K751, K752, K753, K754, K758, K759

Cancer

Cancer code, self-reported, UKB field 20001 -1, 1001, 1002, 1003, 1004, 1005, 1006, 1007, 1008, 1009, 1010, 1011, 1012, 1015, 1016, 1017, 1018, 1019, 1020, 1021, 1022, 1023, 1024, 1025, 1026, 1027, 1028, 1029, 1030, 1031, 1032, 1033, 1034, 1035, 1036, 1037, 1038, 1039, 1040, 1041, 1042, 1043, 1044, 1045, 1046, 1047, 1048, 1050, 1051, 1052, 1053, 1055, 1056, 1058, 1059, 1060, 1061, 1062, 1063, 1064, 1065, 1066, 1067, 1068, 1070, 1071, 1072, 1073, 1074, 1075, 1076, 1077, 1078, 1079, 1080, 1081, 1082, 1084, 1085, 1086, 1087, 1088, 99999

Diabetes (irrespective of type, used in ASCVD Risk Estimator input)

Non-cancer illness code, self-reported, UKB field 20002 1220, 1222, 1223

Diagnoses - ICD9, UKB field 41271 250, 2500, 25000, 25001, 25009, 2501, 25010, 25011, 25019, 2502, 25020, 25021, 25029, 2503, 2504, 2505, 2506, 2507, 2509, 25090, 25091, 25099, 3572, 3620, 7751

Diagnoses - ICD10, UKB field 41270 E10, E100, E101, E102, E103, E104, E105, E106, E107, E108, E109, E11, E110, E111, E112, E113, E114, E115, E116, E117, E118, E119, E12, E120, E121, E122, E123, E124, E125, E126, E127, E128, E129, E13, E130, E131, E132, E133, E134, E135, E136, E137, E138, E139, E14, E140, E141, E142, E143, E144, E145, E146, E147, E148, E149, G590, G632, H280, H360, M142, N083, O240, O241, O242, O243, P702

Diabetes type 1

Non-cancer illness code, self-reported, UKB field 20002 1220

Diagnoses - ICD9, UKB field 41271 25001, 25003, 25011, 25013, 25021, 25023, 25031, 25033, 25041, 25043, 25051, 25053, 25061, 25063, 25071, 25073, 25081, 25083, 25091, 25093

Diagnoses - ICD10, UKB field 41270 E10, E100, E101, E102, E103, E104, E105, E106, E107, E108, E109

Diabetes type 2

Non-cancer illness code, self-reported, UKB field 20002 1223

Diagnoses - ICD9, UKB field 41271 25000, 25002, 25010, 25012, 25020, 25022, 25030, 25032, 25040, 25042, 25050, 25052, 25060, 25062, 25070, 25072, 25080, 25082, 25090, 25092

Diagnoses - ICD10, UKB field 41270 E11, E110, E111, E112, E113, E114, E115, E116, E117, E118, E119

Hypertension

Non-cancer illness code, self-reported, UKB field 20002 1065, 1072

Diagnoses - ICD9, UKB field 41271 401, 4010, 4011, 4019, 402, 4020, 4021, 4029, 403, 4030, 4031, 4039, 404, 4040, 4041, 4049, 405, 4050, 4051, 4059, 4160, 4372, 5723

Diagnoses - ICD10, UKB field 41270 I10, I11, I110, I119, I12, I120, I129, I13, I130, I131, I132, I139, I15, I150, I151, I152, I158, I159, I270, I272, I674, K766

Kidney problem

Cancer code, self-reported, UKB field 20001 1034

Non-cancer illness code, self-reported, UKB field 20002 1192, 1197, 1405, 1427, 1519

Diagnoses - ICD9, UKB field 41271 189, 1890, 1898, 1980, 223, 2230, 2238, 2239, 5808, 5818, 5828, 5838, 5839, 5848, 589, 5890, 5891, 5899, 590, 5909, 592, 5920, 593, 5931, 5932, 5938, 59389, 5939, 75300, 75301, 7531, 75310, 75311, 75312, 75313, 75318, 7533, 75330, 75331, 75332, 75333, 75334, 75338, 75390, 7944, 866, 8660, 8661, E8702, E8712, E8722, E8742, E8791, V420, V594

Diagnoses - ICD10, UKB field 41270 C64, C790, N181, N182, N183, N184, N185, N20, N200, N202, N26, N27, N270, N271, N279, N28, N280, N281, N288, N289, N29, N290, N291, N298, Q60, Q61, Q611, Q612, Q613, Q615, Q618, Q619, Q63, Q630, Q631, Q632, Q633, Q638, Q639, R944, S370, S3700, S3701, T861, Y602, Y612, Y622, Y841, Z524, Z905, Z940

Operative procedures - OPCS3, UKB field 41273 5611, 564, 5641, 5642, 5643, 565, 566, 5661, 5662, 567, 5671, 5672, 568, 579, 5792, 5794, 5795

Operative procedures - OPCS4, UKB field 41272 M01, M011, M012, M013, M014, M015, M018, M019, M02, M024, M026, M027, M028, M029, M03, M031, M032, M038, M039, M04, M041, M042, M043, M048, M049, M05, M054, M055, M058, M059, M06, M061, M062, M068, M069, M07, M071, M072, M078, M079, M08, M081, M082, M083, M084, M088, M089, M09, M091, M092, M093, M094, M098, M099, M10, M101, M103, M104, M105, M108, M109, M11, M111, M112, M113, M118, M119, M13, M131, M132, M133, M134, M135, M137, M138, M139, M14, M141, M148, M149, M15, M158, M159, M16, M161, M162, M168, M169, M17, M171, M172, M173, M174, M175, M178, M179, M196, X451

Liver problem

Non-cancer illness code, self-reported, UKB field 20002 1136, 1158

Diagnoses - ICD9, UKB field 41271 070, 0700, 0701, 0702, 0703, 0704, 0705, 0706, 0709, 1220, 1225, 1228, 1530, 155, 1550, 1551, 1552, 156, 1561, 1568, 1977, 2115, 2308, 2353, 27102, 27103, 27761, 4562, 570, 5709, 571, 5714, 5715, 57150, 57151, 57152, 57158, 57159, 5718, 5719, 572, 5720, 5722, 5728, 573, 5730, 5734, 5738, 5739, 74745, 7516, 75160, 75161, 75162, 75165, 75167, 76782, 7742, 77440, 77441, 77448, 7948, 864, 8640, 8641, V427

Diagnoses - ICD10, UKB field 41270 A064, B15, B150, B159, B16, B160, B161, B162, B169, B17, B170, B171, B172, B178, B179, B18, B180, B181, B182, B188, B189, B19, B190, B199, B251, B581, B670, B675, B678, B942, C183, C22, C220, C221, C223, C224, C227, C229, C240, C787, D015, D134, D135, D376, P150, P353, Q266, Q44, Q446, Q447, R932, R945, S361, S3610, S3611, T864, Z225, Z526, Z944

Operative procedures - OPCS3, UKB field 41273 5005, 502, 5021, 509, 5091, 5092, 5093, 5094, 5097

Operative procedures - OPCS4, UKB field 41272 J01, J011, J012, J013, J014, J015, J018, J019, J02, J023, J024, J025, J028, J029, J03, J031, J032, J033, J034, J035, J038, J039, J04, J041, J042, J043, J048, J049, J05, J051, J052, J053, J058, J059, J06, J061, J062, J068, J069, J07, J071, J072, J073, J074, J078, J079, J08, J081, J083, J088, J089, J10, J101, J103, J104, J105, J106, J107, J108, J109, J11, J111, J112, J113, J114, J115, J116, J117, J118, J119, J12, J121, J122, J123, J124, J125, J126, J127, J128, J129, J13, J131, J132, J138, J139, J14, J141, J142, J148, J149, J15, J151, J152, J153, J158, J159, J16, J161, J162, J168, J169, J275, J29, J291, J292, J293, J294, J298, J299, J311, J312, J401, J471, J472, J473, J474, J485, J486, J505, J507, J761, J77, J771, J778, J779, O301, O341, O342, O343, O344, O345, T876, X43, X438, X439, X863, X864, Z301, Z376, Z396

Obesity

Any individual with BMI, UKB field 21001, larger or equal to 30.

ASCVD

Non-cancer illness code, self-reported, UKB field 20002 1081, 1074, 1082, 1583

Diagnoses - ICD9, UKB field 41271 4139, 4401, 413, 4402, 4140, 4119, 4370, 411, 4148, 4408, 4400, 4149, 4371, 4359, 440, 414, 4409, 435

Diagnoses - ICD10, UKB field 41270 I7011, I20, I700, I7091, M6228, M6224, M6227, I258, I259, M6222, I7021, P910, I708, P294, I250, I7080, M6226, I256, M6223, G458, I70, I24, I672, I64, I209, I7010, I7001, M6221, I7081, I702, I251, I709, I25, I208, I249, M6220, G459, N280, I7000, I7020, I248, G45, I201, M6229, I255, G463, G464, I200, M6225, I701, I7090, I694, M622

Operative procedures - OPCS4, UKB field 41272 U543

In addition, ASCVD cases also included any sample that indicated angina, heart attack or stroke in the self-reported UKB field 6150.

S4.2 Time between condition onset and biomarker measurement

In the current analysis, we did not take into account the time of disease onset as compared to the time of the biomarker measurement(s). As we emphasized in the main paper, *prediction* thus refers to predicting the current or future case status given a set of biomarker measurements. A sample was defined a case regardless if the condition onset was prior to the measurement or after. Similarly, all samples with no current records of the condition were defined as controls, with no regard to age and the likelihood of future onsets.

The distribution of time difference between the condition onset and the first biomarker measurement is depicted in Figure S27. Note that the UKB follow-up data after the first biomarker measurement only contains a fraction of all samples that attended the first assessment.

The distribution of the time difference from first biomarker measurement to the onset of ASCVD and the time histograms for all the biomarker measurements are shown in Figure S28.

S4.3 Input data to the ASCVD Risk Estimator

The ASCVD Risk Estimator [63] use 13-14 input data fields. For our evaluation, we used the following UKB data for each of the required inputs. The UKB field IDs are noted within parentheses.

Age: Time difference from year(34) and month(52) of birth to date of attending assessment center(21003), rounded to full years.

Sex: Self-reported sex(31), only including samples with XX or XY chromosome pairs.

Race: Self-reported ethnic background(21000).

Systolic Blood Pressure: Systolic blood pressure, automated reading(4080)

Diastolic blood pressure: Diastolic blood pressure, automated reading(4079)

Total Cholesterol: Cholesterol(30690), converted to mg/dL.

HDL Cholesterol: HDL cholesterol(30760), converted to mg/dL.

LDL Cholesterol: LDL direct(30780), converted to mg/dL.

History of Diabetes: Using the combination of the (general) diabetes definition in section S4.1, only assigning case if onset was prior to assessment center visit, and the self-reported use of insulin(6177 coding 3).

Smoker: Smoking status(20116).

Time since stopped smoking: Time difference between the age field above and age stopped smoking(2897).

On Hypertension Treatment: Self-reported use of blood pressure medication(6177 coding 2) or any of the following medications listed in treatment/medication code(20003): chlorthalidone, chlorothiazide, hydrochlorothiazide, indapamide, metolazone, amiloride, spironolactone, triamterene, bumetanide, furosemide, torsemide, amiloride hydrochloride/hydrochlorothiazide, spironolactone/hydrochlorothiazide, triamterene/hydrochlorothiazide, acebutolol, atenolol, betaxolol, bisoprolol, bisoprolol/hydrochlorothiazide, metoprolol tartrate, metoprolol succinate, nadolol, pindolol, propranolol, solotol, timolol, benazepril, captopril, enalapril, fosinopril, lisinopril, moexipril, perindopril, quinapril, ramipril, trandolapril, candesartan, eprosartan, irbesartan, losartan, telmisartan, valsartan, amlodipine, diltiazem, felodipine, isradipine, nicardipine, nifedipine, nisoldipine, verapamil, doxazosin, prazosin, terazosin, carvedilol, labetalol, methyldopa, clonidine, guanfacine, hydralazine, minoxidil, eplerenone, spironolactone, and aliskiren.

On a Statin: Self-reported use of cholesterol lowering medication(6177 coding 1) or any medication containing "statin" in its name listed in treatment/medication code(20003).

On Aspirin Treatment: Any medication containing "aspirin" in its name listed in treatment/medication code(20003).

S4.4 Risk conversion for ASCVD predictors

The risk score output from the linear regression was converted into a risk estimate in percentage by the following steps.

1. The risk scores were calculated for the 185,106 samples used in training and binned into .05% quantiles. The actual disease prevalence was then calculated in each bin.

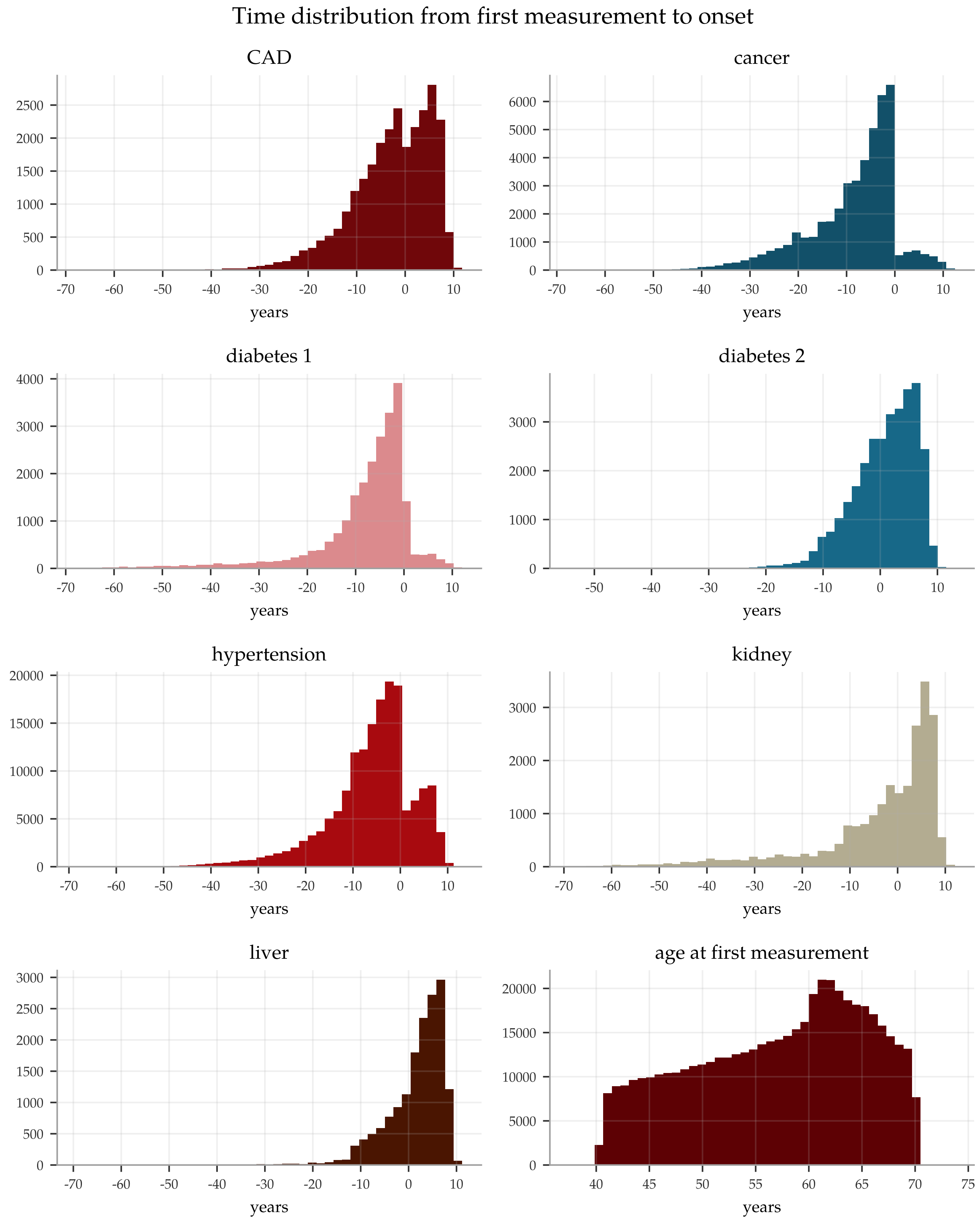

Figure S27: Distributions of time differences between condition onset and the time of the first biomarker measurement. Positive numbers indicate that the onset was after the first measurement. Also, note that the amount of data available after the first measurement is much smaller why there are discontinuous drops at 0 in each distribution. These data use the UKB field 53 "Date of visiting assessment centre" and has not filtered out individuals for which no biomarker measurements were recorded.

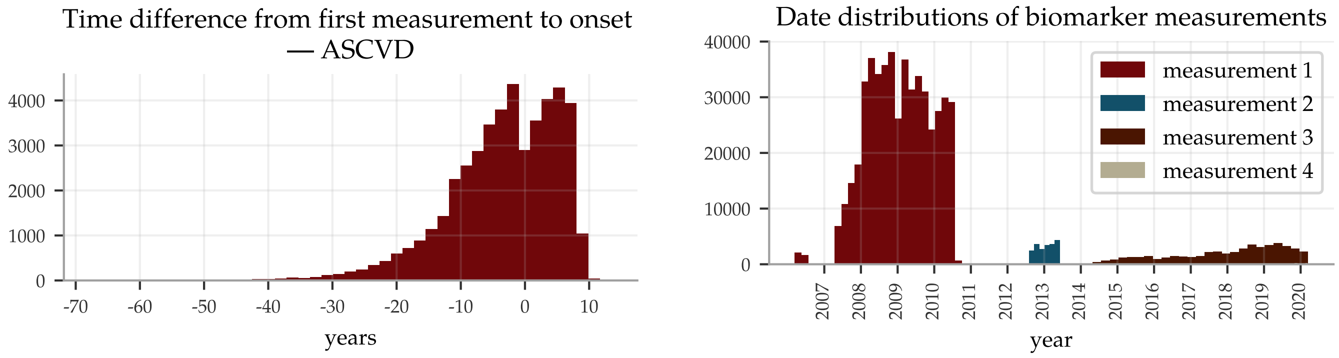

Figure S28: **Left:** Time difference from first biomarker measurement to the onset of ASCVD. A positive number indicates an onset after the first measurement. **Right:** The time distribution of the four instances of biomarker measurements. These data use the UKB field 53 ”Date of visiting assessment centre” and has not filtered out individuals for which no biomarker measurements were recorded.

2. Two maps from risk scores to risk estimates were then calculated by pairing each risk score with its corresponding bin prevalence, sorting all these pairs according to risk score and then calculating the rolling average of the prevalence values using a triangular window. The two maps used window size 2000 and 9000, respectively. The map with the larger window was applied up to prevalence 0.53; above that limit the smaller window was required to capture the extremes.
3. The last step smoothed the conversion function by linear interpolation between each sample among the 100 lowest risk scores, then between each 2000th sample up to the $\sim 20,000$ highest risk scores where a step size of 2 was used.

S4.5 Coefficient sizes

The coefficient sizes for each BMRS predictor can be found in figures **Figure S29** and **Figure S30**.

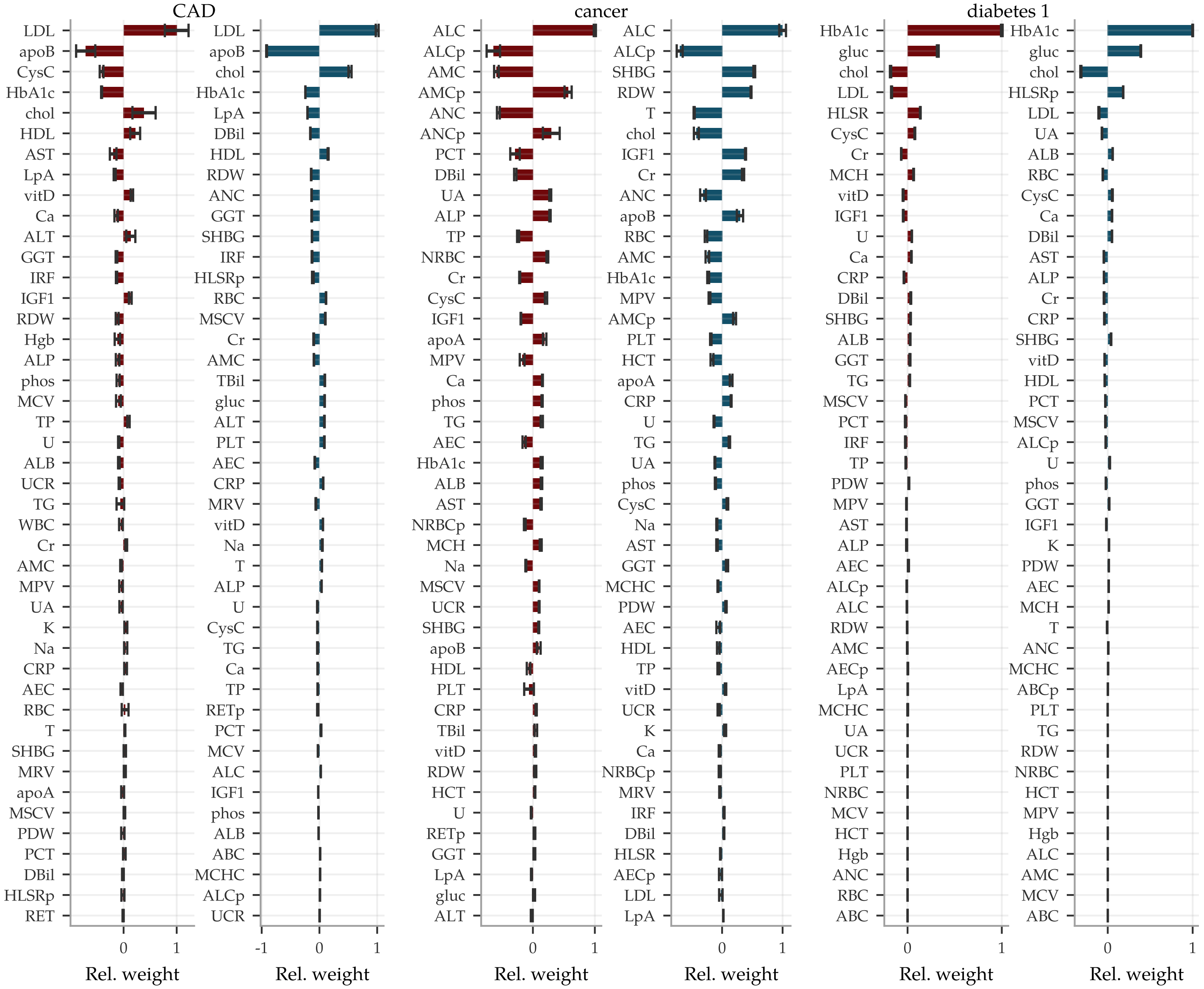

Figure S29: Coefficient sizes (1 of 3) for BMRS predictors, ■ women ■ men. The bars show means of 5 predictors with error bars indicating the standard deviations. See figures **Figure S30** and **Figure S31** for the other conditions.

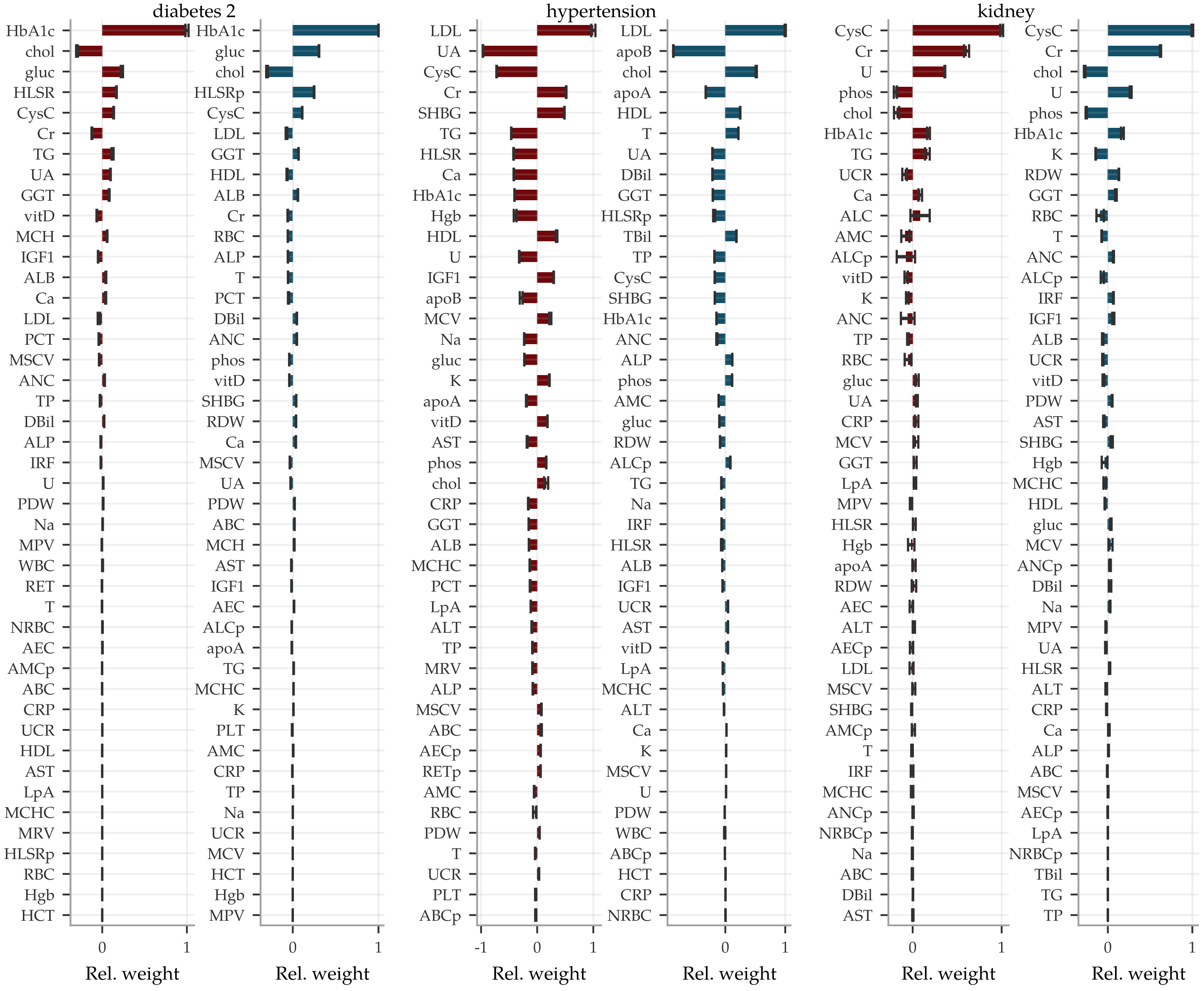

Figure S30: Coefficient sizes (2 of 3) for BMRS predictors, ■ women ■ men. The bars show means of 5 predictors with error bars indicating the standard deviations. See figures **Figure S29** and **Figure S31** for the other conditions.

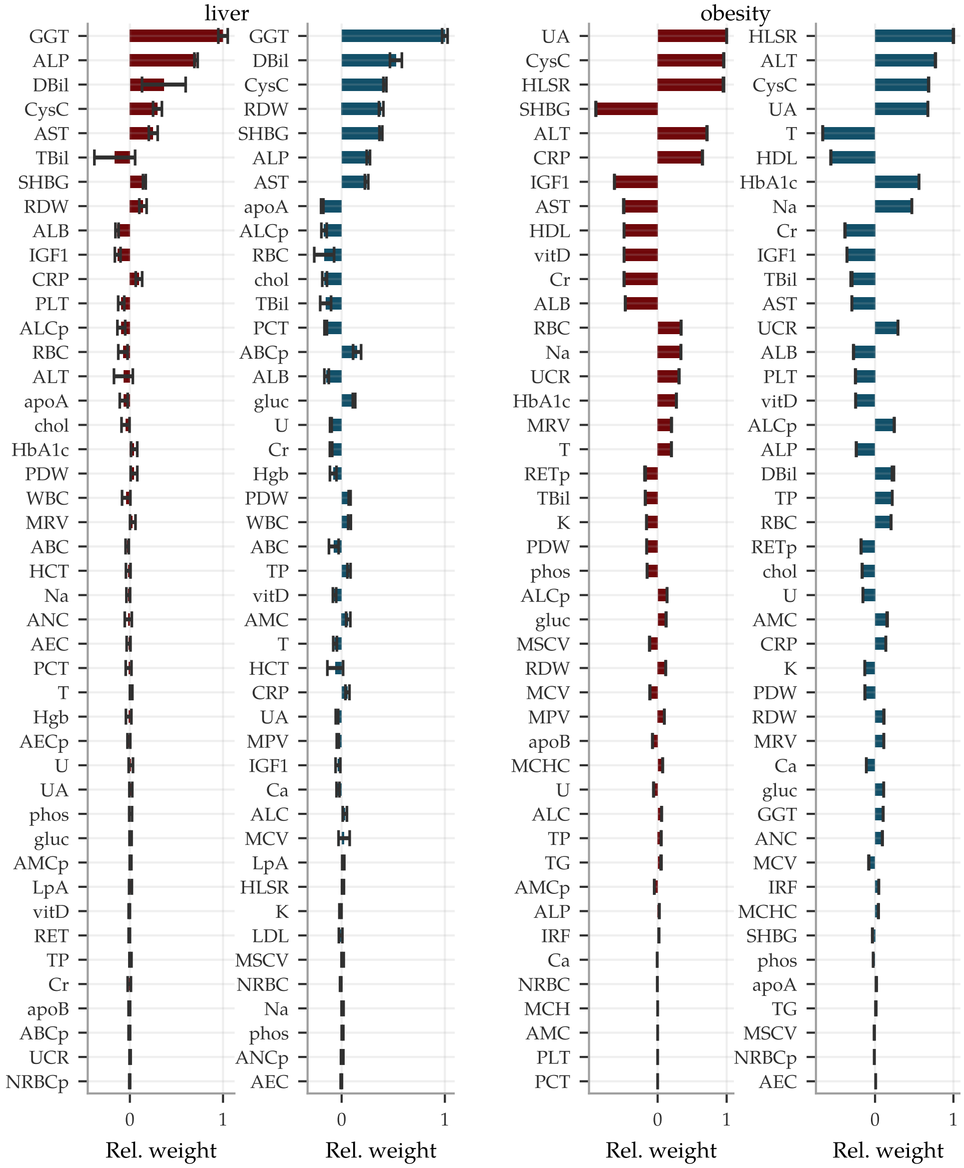

Figure S31: Coefficient sizes (3 of 3) for the BMRS predictors, ■ women ■ men. The bars show means of 5 predictors with error bars indicating the standard deviations. The other conditions are shown in figures **Figure S29** and **Figure S30**.

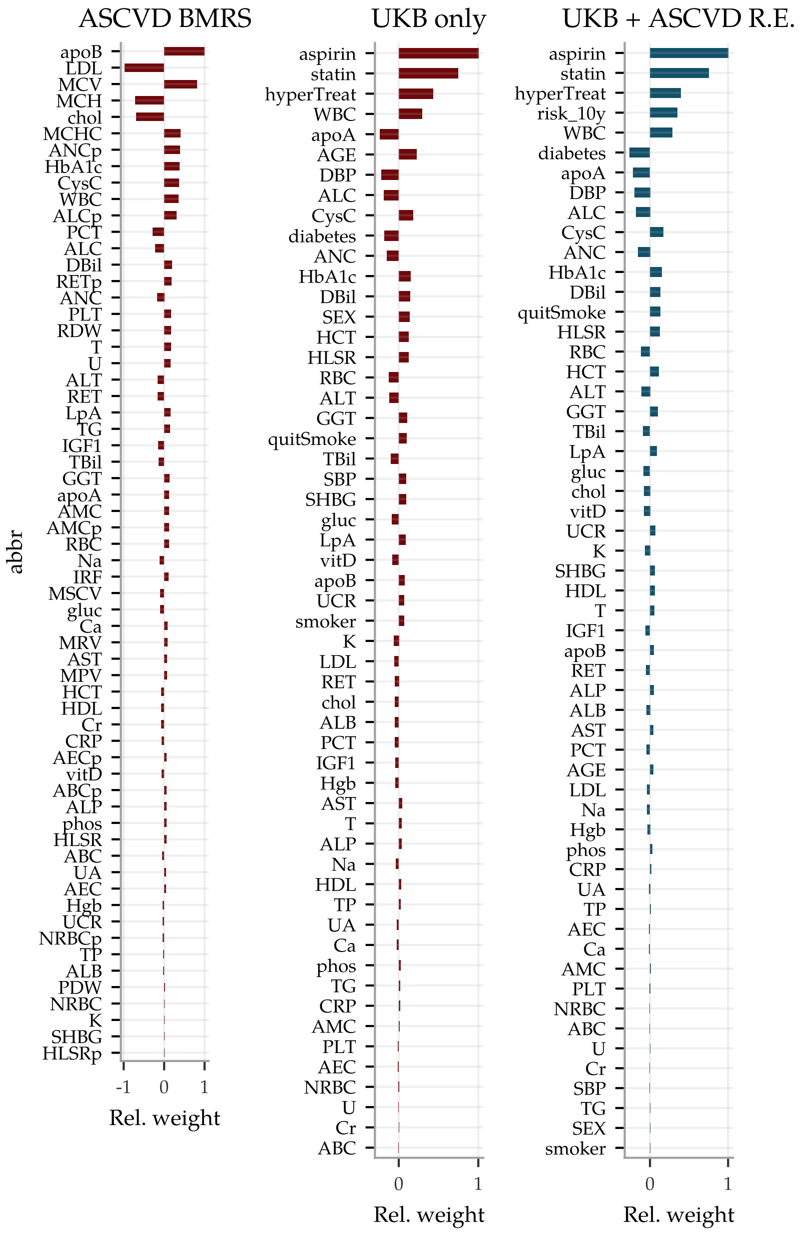

Figure S32: Coefficient sizes for the different ASCVD predictors. From left to right, BMRS predictor for ASCVD, risk predictor using UKB data only but both biomarkers and all input used in the ASCVD Risk Estimator, and to the right using the biomarkers and both input and output for the ASCVD Risk Estimator.

S4.6 AUCs and sample sizes

The AUCs and sample sizes for the BMRS predictors are shown in **Table S4**, while the sample sizes and numerical values for the AUCs of the gBMRS predictors are listed in **Table S5**.

Sample sizes

	women								men							
	European		South Asian		East Asian		African		European		South Asian		East Asian		African	
CC	ctrls	cases	ctrls	cases	ctrls	cases	ctrls	cases	ctrls	cases	ctrls	cases	ctrls	cases	ctrls	cases
CAD	8842	174	1369	30	443	9	1361	18	8745	861	2329	365	329	9	1496	47
cancer	8076	940	1352	47	433	19	1323	56	8897	709	2642	52	329	9	1485	58
diabetes 1	8785	232	1267	132	434	18	1280	99	9031	575	2212	482	320	18	1381	162
diabetes 2	8702	314	1223	176	425	27	1239	140	8907	699	2148	546	319	19	1372	171
hypertension	6432	2584	968	431	339	113	751	628	5859	3747	1553	1141	242	96	905	638
kidney	8745	271	1363	36	441	11	1338	41	9111	495	2538	156	327	11	1462	81
liver	8754	263	1367	32	437	15	1347	32	9315	291	2607	87	321	17	1486	57
obesity	7121	1895	1073	326	434	18	763	616	7367	2239	2218	476	320	18	1076	467

AUCs

	women				men			
ANCESTRY	European	South Asian	East Asian	African	European	South Asian	East Asian	African
AUC	AUC	AUC	AUC	AUC	AUC	AUC	AUC	AUC
CAD	0.743 ± 0.001	0.762 ± 0.003	0.819 ± 0.007	0.682 ± 0.004	0.734 ± 0.000	0.744 ± 0.000	0.779 ± 0.002	0.694 ± 0.000
cancer	0.561 ± 0.001	0.653 ± 0.002	0.696 ± 0.006	0.682 ± 0.002	0.597 ± 0.001	0.656 ± 0.002	0.646 ± 0.007	0.639 ± 0.002
diabetes 1	0.947 ± 0.000	0.936 ± 0.000	0.935 ± 0.001	0.919 ± 0.001	0.950 ± 0.000	0.931 ± 0.000	0.831 ± 0.001	0.909 ± 0.000
diabetes 2	0.937 ± 0.000	0.917 ± 0.000	0.955 ± 0.001	0.891 ± 0.000	0.921 ± 0.000	0.881 ± 0.000	0.794 ± 0.000	0.886 ± 0.000
hypertension	0.710 ± 0.000	0.722 ± 0.000	0.766 ± 0.000	0.706 ± 0.000	0.717 ± 0.000	0.749 ± 0.000	0.665 ± 0.000	0.666 ± 0.000
kidney	0.697 ± 0.002	0.795 ± 0.003	0.741 ± 0.005	0.690 ± 0.003	0.668 ± 0.000	0.763 ± 0.001	0.791 ± 0.002	0.714 ± 0.001
liver	0.644 ± 0.004	0.695 ± 0.004	0.591 ± 0.018	0.635 ± 0.011	0.643 ± 0.002	0.711 ± 0.002	0.634 ± 0.006	0.666 ± 0.002
obesity	0.877 ± 0.000	0.820 ± 0.000	0.822 ± 0.000	0.811 ± 0.000	0.832 ± 0.000	0.750 ± 0.000	0.880 ± 0.000	0.753 ± 0.000

Table S4: AUCs for BMRS predictors. Listed values are mean ± the standard deviation for 5 different predictors, all trained on European ancestry. These are the numerical values for figure 5 in the main document.

	women			men		
	AUC	ctrls	cases	AUC	ctrls	cases
CAD	0.493 ± 0.010	2751	40	0.492 ± 0.001	2680	230
cancer	0.505 ± 0.002	2506	285	0.488 ± 0.005	2668	242
diabetes 1	0.557 ± 0.003	2729	62	0.650 ± 0.002	2760	150
diabetes 2	0.624 ± 0.009	2709	82	0.637 ± 0.003	2738	172
hypertension	0.558 ± 0.002	2074	717	0.514 ± 0.001	1831	1079
kidney	0.527 ± 0.005	2713	78	0.522 ± 0.003	2769	141
liver	0.524 ± 0.008	2719	72	0.551 ± 0.008	2830	80
obesity	0.574 ± 0.003	2236	555	0.564 ± 0.007	2309	601

Table S5: AUCs and sample sizes for the gBMRS predictors. The AUCs are listed as means ± the standard deviations for 5 different predictors, trained and evaluated on European ancestry. These are the numerical values for figure 7 in the main document.

S4.7 Risk score distributions

The BMRS and gBMRS predictors were applied to evaluation sets of European ancestry with 9016/9606 and 2791/2910 women/men, respectively. The risk score distributions cases and controls for each sex are shown in **Figure S33** for BMRS and **Figure S34** for gBMRS. Overlaid are QQ-plots for which cases and controls are separately compared to normal distributions of the same means and standard deviations to illustrate non-normally distributed deviations.

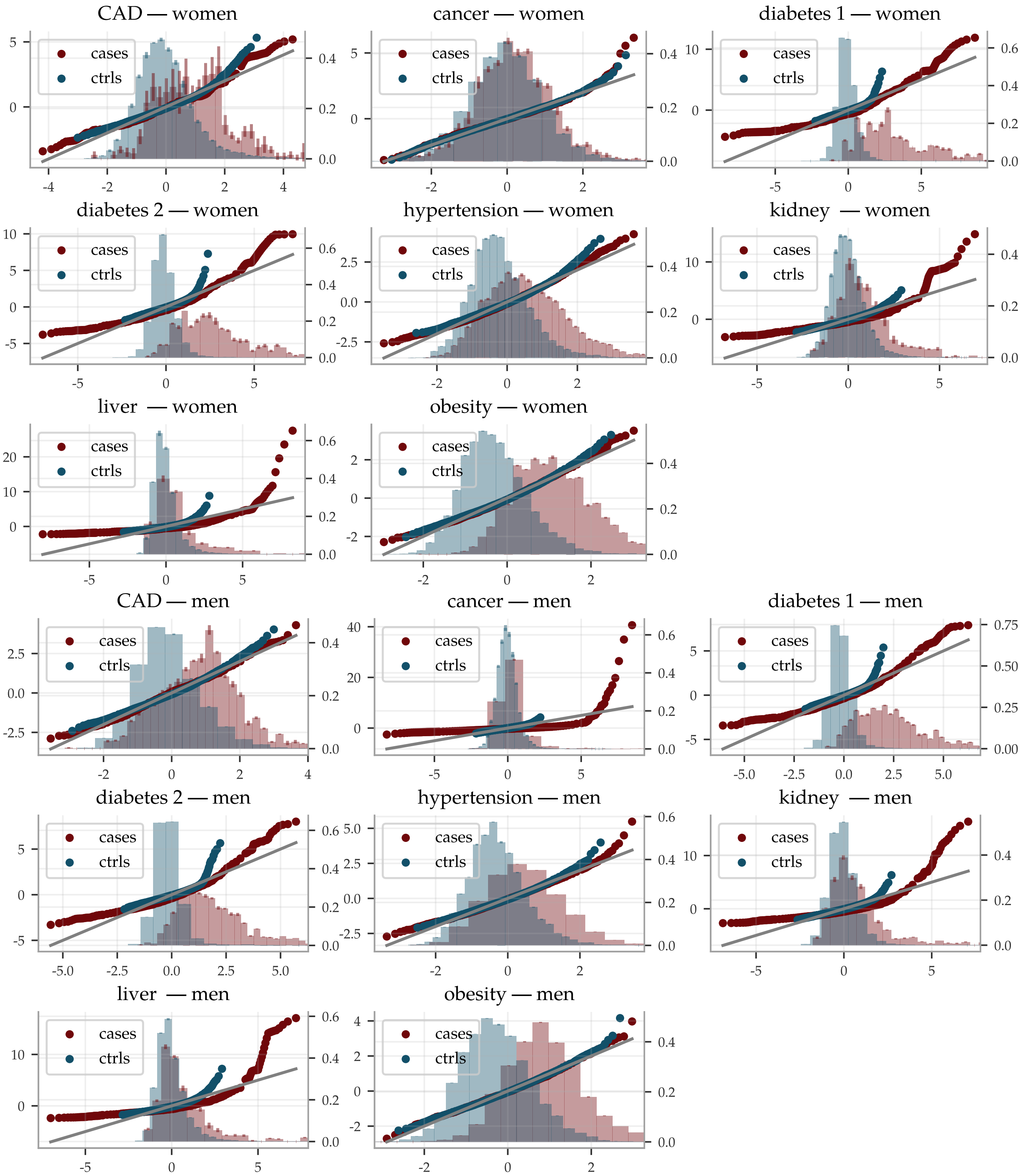

Figure S33: QQ-plots and distribution histograms for the BMRS predictors. The QQ-plots compare case and control distributions with normal distributions of the same mean and standard deviations and use the y-axis to the left. The histograms are normalized to unit area and use the y-axis to the right, with error bars indicating the standard deviation for 5 different predictors. The evaluations are on 9016 women and 9606 men of European ancestry not used in training.

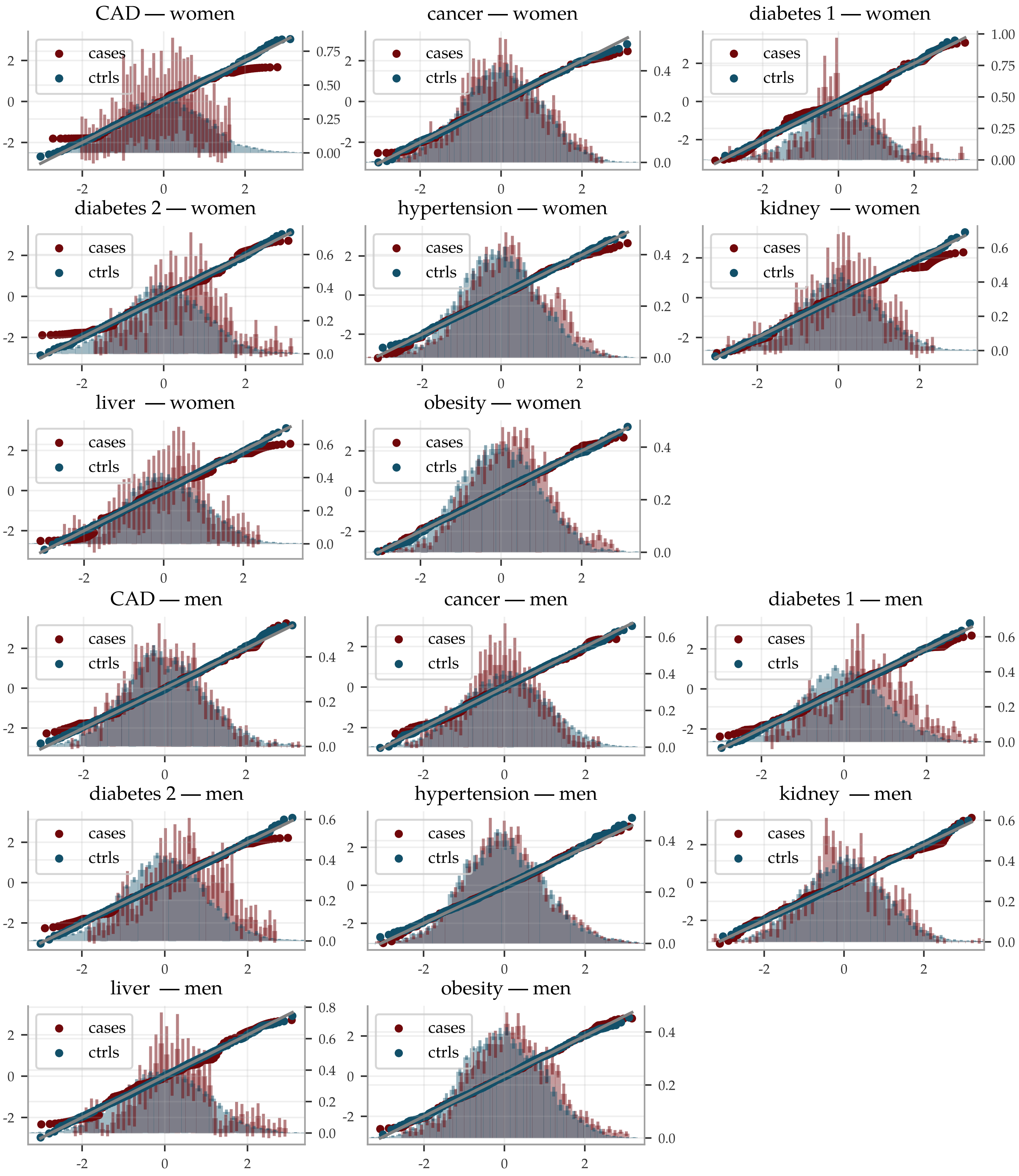

Figure S34: QQ-plots and distribution histograms for the gBMRS predictors. The QQ-plots compare case and control distributions with normal distributions of the same mean and standard deviations and use the y-axis to the left. The histograms are normalized to unit area and use the y-axis to the right, with error bars indicating the standard deviation for 5 different predictors applied to 5 different PGS. The evaluations are on 2791 women and 2910 men of European ancestry not used in training.
